# Efficient and Robust Semi-supervised Estimation of Average Treatment Effect with Partially Annotated Treatment and Response

**Published:** 2025

**Authors:** Jue Hou, Rajarshi Mukherjee, Tianxi Cai

**Affiliations:** Division of Biostatistics, University of Minnesota School of Public Health, Minneapolis, MN 55455, USA.; Department of Biostatistics, Harvard T.H. Chan School of Public Health, Boston, MA 02120, USA; Department of Biostatistics, Harvard T.H. Chan School of Public Health, Department of Biomedical Informatics, Harvard Medical School, Boston, MA 02120, USA

**Keywords:** semi-parametric efficiency, double robustness, high-dimensional regression, semi-supervised learning

## Abstract

A notable challenge of leveraging Electronic Health Records (EHR) for treatment effect assessment is the lack of precise information on important clinical variables, including the treatment received and the response. Both treatment information and response cannot be accurately captured by readily available EHR features in many studies and require labor-intensive manual chart review to precisely annotate, which limits the number of available gold standard labels on these key variables. We considered average treatment effect (ATE) estimation when 1) exact treatment and outcome variables are only observed together in a small labeled subset and 2) noisy surrogates of treatment and outcome, such as relevant prescription and diagnosis codes, along with potential confounders are observed for all subjects. We derived the efficient influence function for ATE and used it to construct a semi-supervised multiple machine learning (SMMAL) estimator. We justified that our SMMAL ATE estimator is semi-parametric efficient with B-spline regression under low-dimensional smooth models. We developed the adaptive sparsity/model doubly robust estimation under high-dimensional logistic propensity score and outcome regression models. Results from simulation studies demonstrated the validity of our SMMAL method and its superiority over supervised and unsupervised benchmarks. We applied SMMAL to the assessment of targeted therapies for metastatic colorectal cancer in comparison to chemotherapy.

## 1 Introduction

The 21st Century Cures Act and the Prescription Drug User Fee Act VII have shone a spotlight on the use of real-world evidence, generated from real-world data, to support regulatory-decision making on drug effectiveness. Large scale electronic health records (EHRs) data are being increasingly used for creating the real-world evidence on treatment effectiveness or efficacy (Franklin et al., 2021). In addition to the observational nature, another notable challenge in leveraging EHR for treatment effect assessment lies in the lack of readily available data for key clinical variables, including the treatment being investigated and the outcome of interest. Response variables such as disease progression may not be well represented by readily available EHR features (Bartlett et al., 2019). Treatment information can be partially captured but not always accurately reflected by procedure codes or medication prescription codes. New therapies may not be well coded in the introduction stage immediately after regulatory approval, and treatment initiation may be later than prescription date due to external factors such as insurance approval delay. For example in a real-world evidence study comparing chemotherapies and targeted therapies as first-line treatment for metastatic colorectal cancer, we discovered based on chart-review of 100 patients by a medical expert that 1) the progression-free-survival (PFS) outcomes were poorly structured in EHRs without clear indicators for progression or complete mortality data, and 2) the medication codes or natural language processing (NLP) identified mentions in notes could not accurately capture the use of targeted therapies (see Table 2).

Although it is possible to improve treatment or response definition by combining multiple EHR features through rule based or machine learning algorithms, these EHR derived features are at best good “surrogates” for approximating the true treatment or response information at patient level. Compared to the classic definition of surrogate, the notion of surrogate in retrospective EHR studies shares the availability trait but differs in the temporal order and causal pathway. In the advanced stage cancer trials or prospective studies, the progress-free-survival is often used as surrogate for overall survival because progression sometimes can be captured at an earlier time. In EHR studies, however, researchers do not have readily available progression data Y unless they perform the labor intensive manual chart review (Griffith et al., 2019), so it is natural to borrow information from the documentations about progression S like occurrence of diagnosis codes about secondary malignant neoplasm or NLP identified mention of metastasis at distant parts of body. These documentations S are considered as “surrogates” because 1) they can partially indicate progression, and 2) they are accessible earlier during the research process. Since the documentations S were recorded according to the true progression status Y, it is more reasonable to consider the true progression status temporally preceded and casually affected the documentation, Y→S in a causal diagram. Directly using these surrogates as true treatment and outcome which would potentially induce bias in the subsequent analysis (Beaulieu-Jones et al., 2020). On the other hand, annotating exact treatment and response variables via manual chart review by domain expert is resource intensive, leading to limited sample size for gold standard labels on these key data. It is thus of great practical significance to leverage both the small number of gold standard labels and the vast unlabeled data to derive unbiased and efficient inference about the average treatment effect (ATE), fundamentally a nested problem with both missing data and causal inference components. When the labeling proportion is too small for standard (missing data) positivity assumption, the setting is often referred to as the semi-supervised learning (SSL).

Additional challenges arise from the high dimensionality of potential confounders. Unlike traditional cohort studies with a pre-specified number of clinical variables, EHRs provide rich data on a broader range and larger number of confounding factors (Hou et al., 2021c). Furthermore, multiple EHR features may be necessary to represent one specific clinical variable, further amplifying the dimensionality of features necessary to capture the underlying confounding factors. The complexity of the models from the high-dimensionality also increases the risk of model mis-specification for the propensity score (PS) and the outcome regression (OR). To the best of our knowledge, no method currently exists to estimate ATE under the SSL setting when both the treatment group, denoted by A, and the response, denoted by Y, are only observed in a small subset of the full data. We focus on the missing data patterns resulting from the lack of readily available data on the exact clinical information like treatment A and outcomes Y. For small subset, manual annotations can be created to recover the exact Y and A, but researchers have to rely on scalable yet imperfect computational tools to extract treatment outcome information over the majority of the vast EHR cohort, producing the surrogates S for Y and A. For conciseness, we refer to this specific SSL setting as *double missing* SSL. In this paper, we address the methodology gap by proposing Semi-supervised **M**ultiple **MA**chine **L**earning (SMMAL) estimators for ATE that leverage both the fully observed surrogates for Y and A, denoted by S, and the partially observed gold standard labels on Y and A.

Under the supervised setting where both A and Y are observed, much progress has been made in recent years on estimation of ATE with confounding adjustment from machine-learning and/or high-dimensional regression. In the low-dimensional setting, the estimation of ATE is a well studied problem including procedures that achieve semi-parametric efficiency and double robustness (Robins et al., 1994; Bang and Robins, 2005). Extension to the high-dimensional setting, however, is not straightforward due to the slower convergence rates in the estimated model parameters and the difficulty posed not only by the bias and variance trade-off in the process of regularization but also by the inherent information theoretic barriers to obtaining fast enough estimation rates in high dimensional problems. Similar challenges arise when incorporating more flexible machine-learning models to overcome model mis-specifications. Following intuitions parallel to the low-dimensional setting, flexible approaches for confounder adjustments have been proposed via modeling of PS and OR, including $L_{1}$ regularized regression (Farrell, 2015), neural network (Farrell et al., 2021), and a general machine learning framework (Chernozhukov et al., 2018). Several methods accommodated the high dimensional confounder and achieved statistical inference on ATE based on consistent estimation for PS and OR, which translated to proper model specification and sparsity for high-dimensional regressions (Belloni et al., 2013; Liu et al., 2021; Hou et al., 2021a; Belloni et al., 2017, e.g.). Tan (2020) proposed a calibrated estimation that leads to valid inference for the average treatment effect even if one of the high-dimensional logistic PS or linear OR model is mis-specified. Smucler et al. (2019) formalized the concept of double robustness in high-dimensional setting by defining the sparsity double robustness and model double robustness properties; and also generalized the idea of Tan (2020) to a wide range of PS and OR models. For data with sample size n and dimension of covariate p, Smucler et al. (2019) defined the sparsity double robustness as producing $\sqrt{n}$-asymptotic normal estimator for ATE from consistently estimated PS and OR models as long as the *product* of sparsities for PS and OR models grow slower than nlog(p). The model double robustness further allows the estimation of either PS or OR model to be inconsistent while still achieving $\sqrt{n}$-asymptotic normal estimation of ATE. Bradic et al. (2019) established a sharper sparsity double robustness property of the calibrated estimation. Unlike the two-model approaches (PS and OR) listed above, Wang and Shah (2020) considered a single model approach in which they debiased the regularized PS model in the inverse probability of treatment weighting estimator to achieve $\sqrt{n}$-inference.

**Figure 1:**
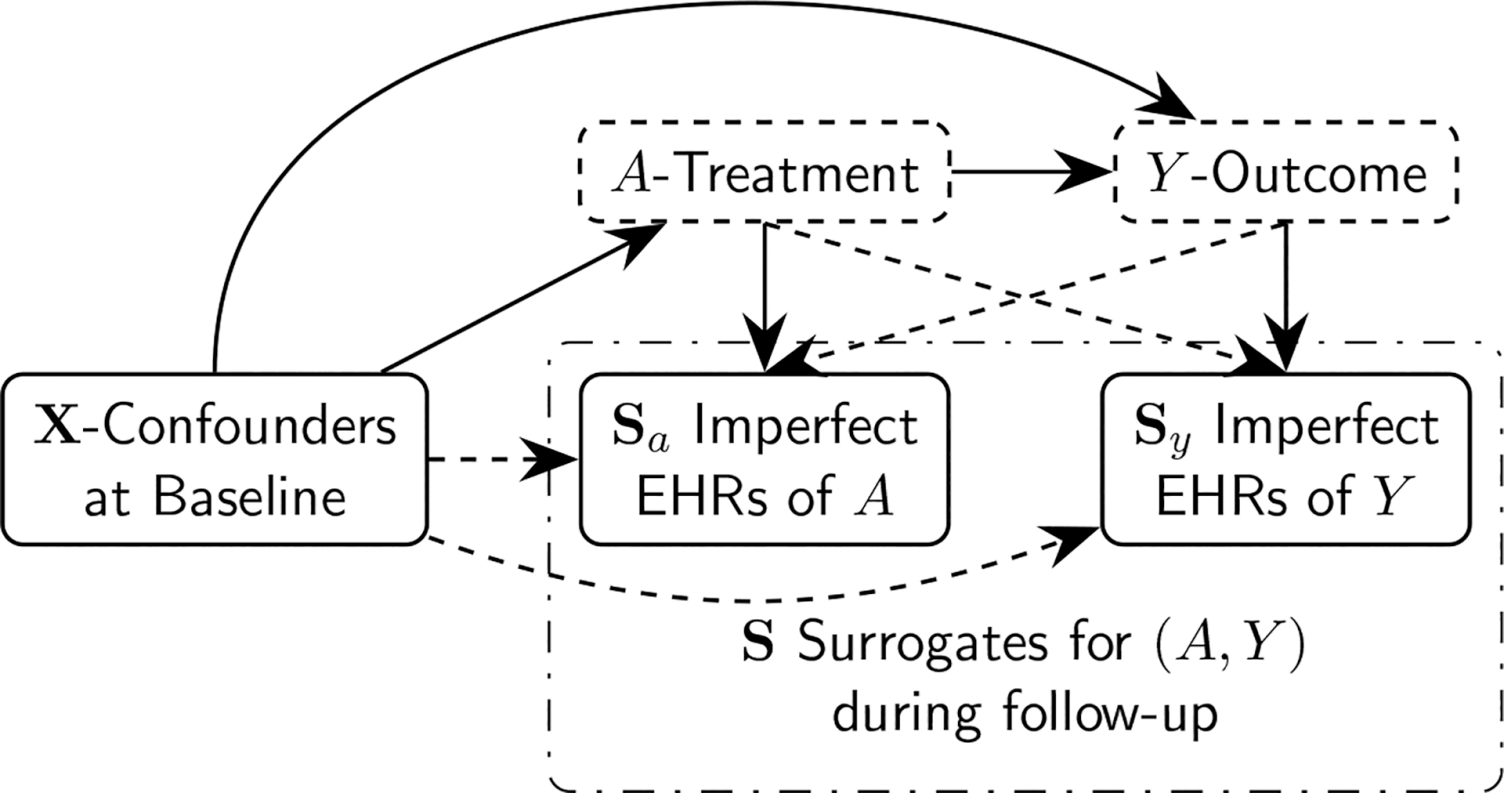
Causal diagrams of double missing SSL setting with missing treatment and outcome. The surrogates S represent the imprecise documentation of A and Y, which should be predictive for A and Y but not affecting the causal identification based on perfect data (Y,A,X).

Semi-supervised estimation for ATE is less studied. Existing literatures focused almost entirely on the setting where Y is observed for patients in the small labeled set of size n while A and surrogates/proxies of Y along with confounders X are observed for all subjects of size N. The semi-supervised learning (SSL) setting refers to the missing data proportion (N-n)/N for Y tending to 1 along an asymptotic sequence where both number of labels and total sample size tend to infinity, n,N→∞. The SSL setting is distinguished from classical missing data problems as the standard (missing data) positivity assumption on observation rate is violated. SSL estimators for the ATE have been proposed by Cheng et al. (2021) when Y is missing-completely-at-random (MCAR) and by Zhang et al. (2023) and Kallus and Mao (2024) when Y is missing-at-random (MAR). However, these methods cannot be easily adapted to the setting where both Y and A are missing. The missingness in A is fundamentally different from the missingness in Y since treatment is an internal node in the causal pathway “confounder $\left(X\right)\to \text{treatment}(A)\to \text{outcome}(Y)\to \text{surrogates}(S)$ ” (Figure 1), introducing technical challenges on the projection by conditional expectation in the semi-parametric analysis.

In this paper, we propose an efficient and robust SSL estimator for ATE when both Y and A are only observed for a small labeled subset but the confounders X and surrogates S for Y and A are observed for all N patients. We derived the SMMAL estimator by first deriving the efficient influence function for the ATE under this double missing SSL setting and then constructing a cross-fitted multiple machine learning estimator. We subsequently provided a formal characterization of semi-parametric efficiency under the double missing SSL setting with the SMMAL estimator coupled to B-spline regressions over low-dimensional space. We also designed a doubly robust estimator with a two-layer cross-fitted calibrated estimation for high-dimensional logistic PS and OR models. Via cross-fitting and a truncation in initial OR/PS predictions, we relaxed the sparsity assumptions in the initial estimation for PS and OR, previously required for $\sqrt{n}$-inference of ATE (Tan, 2020; Smucler et al., 2019). We further showed that our doubly robust SMMAL estimator attains 1) the rate double robustness when both PS and OR models are correct and 2) model double robustness when one of them is correct, as defined by Smucler et al. (2019). The SMMAL estimator also does not require correct specifications of the imputation models for A or Y for proper inference under MCAR assumption. We summarize our key contributions herein:

1. We formalized the efficient estimation under a general SSL setting (including specifically the double missing SSL setting) with a decaying observation rate that violates the classical (missing data) positivity assumption. Our theory justified the efficiency claims of existing works and can provide benchmark for future work in this direction. A discussion regarding the subtleties and challenges involved in this formalization and subsequent analyses can be found in Remark 6.
2. We laid out a general approach for efficient SSL with a complex missing data structure. Our general approach is particularly convenient when the missing data and dependence patterns render typical projection approach difficult for the semi-parametric theory. A explanation of the challenge from missing treatment under double missing SSL setting can be found in Remark 4, and the general framework is given in Section 7.
3. We made progress in statistical inference on ATE based on the doubly robust estimation with high-dimensional confounders achieving the sparsity/model double robustness. We generalized to the SSL setting the techniques in the existing literatures on calibrated estimation of PS and OR models so that the final ATE estimator has weak dependence on estimation of these models, characterized by small derivatives, also known as the Neyman Orthogonality (Chernozhukov et al., 2018). Using a truncation of initial model prediction, we removed the sparsity requirement in initial estimation. In addition, we demonstrated that the SSL estimation derived from our modified semi-parametric theory contributed to robustness toward estimation of the imputation models. The comparison with related work can be found in Remark 9.

The paper is organized as follows. In Section 2, we introduce our causal inference structure under the double missing SSL setting along with the notations. In Section 3, we first present the efficient influence function, followed by the multiple machine learning estimator and the model multiply robust estimator derived from the efficient influence function. In Section 4, we state the theoretical guarantees of the $\sqrt{n}$-inference on the ATE from our methods, whose proofs are provided in the Supplementary Materials. We also provide the semi-parametric efficiency lower bound for average treatment effect under double missing SSL setting in low-dimensional space. In Section 5, we assess the finite sample performance of our SSL methods and compare them to supervised benchmarks. In Section 6, we apply SMMAL to the real-world evidence study on targeted therapies for metastatic colorectal cancer in comparison with chemotherapy. In Section 7, we offer the efficiency lower bound for general low-dimensional parameters under broader SSL settings with flexible missing data components. In Section 8, we conclude with a brief discussion.

## 2 Setting and notation

For the i-th observation in a study of N subjects, $Y_{i}\in R$ denotes the outcome variable, $A_{i}\in \{0, 1\}$ denotes the treatment group indicator, $R_{i}\in \{0, 1\}$ indicates whether $\left(Y_{i},A_{i}\right)$ is annotated, $S_{i}\in R^{q}$ denotes the surrogates for $Y_{i}$ and $A_{i}$, and $X_{i}\in R^{p+1}$ denotes the vector of potential confounders including 1 as the first element. We use the notations without the subscript indices Y,A,R,S,X to denote the generic versions of these random variables. In EHR studies, routine documentations on treatments and outcomes in the form of digital codes and mentions in narrative notes are often prone to errors (Zhang et al., 2019) and hence can only serve as surrogates S. To ascertain Y and A, researchers may design the sampling scheme for a representative labeled subset, $\left\{i\in [N]:R_{i}=1\right\}$, over which the exact data $\left(Y_{i},A_{i}\right)$ are annotated by medical experts, where [N]={1,…,N}. For those with $R_{i}=0$, exact values of the pair $\left(Y_{i},A_{i}\right)$ are not ascertained, creating the joint missingness of $\left(Y_{i},A_{i}\right)$. The observed data consist of N independent and identically distributed (i.i.d.) random vectors, $𝒟=\left\{D_{i}={\left(R_{i},R_{i}Y_{i},R_{i}A_{i},W_{i}^{⊤}\right)}^{⊤},i=1,\ldots ,N\right\}$, where $W_{i}={\left(X_{i}^{⊤},S_{i}^{⊤}\right)}^{⊤}$.

We assume the MCAR mechanism for the sampling process with

(1) R⫫(Y,A,X,S),

and the number of labelled sample is $n=\sum_{i=1}^{N}R_{i}$ with the proportion of labeled observation being $\rho_{N}=E(R)\in (0, 1)$ with $\rho_{N}\to 0$ as N→∞ while the expected number of labels also grow asymptotically to infinity $\rho_{N}N\to \infty$. Under MCAR formulation, the size of labeled subset n is a random variable asymptotically equivalent to $\rho_{N}N$, as $n/\left(\rho_{N}N\right)=1+o_{p}(1)$. We use a simplified notation “$V_{N}≍U_{N}$ ”, e.g. $n≍\rho_{N}N$, to describe the equivalence in stochastic order, $V_{N}/U_{N}=O_{p}(1)$ and $U_{N}/V_{N}=O_{p}(1)$. To better reflect the dependence on labeled set and compare with supervised benchmarks, we use n instead of $\rho_{N}N$ when describing the asymptotic orders. As the exception, we use $\rho_{N}N$ in the derivation of efficiency lower bound. Extension to MAR is plausible through modeling and estimating the missing data pattern P(R=1∣W) under classical semi-parametric theory, but a few technical and practical challenges exist as we listed in the Section 8. Thorough investigation of the MCAR setting would already provide methodological guidance to the rapidly growing real-world evidence studies in which random subsets are selected for gold-standard validation of intervention and outcome data (Hou et al., 2023). Our MCAR formulation, as opposed to the two sample formulations in existing statistical semi-supervised learning literatures (Chakrabortty et al., 2019; Zhang et al., 2023; Hou et al., 2021b), connects better with existing literature on semiparametric estimation and missing data. The results under MCAR, with minor modification in theoretical derivations, are largely applicable to the other similar formulation like first n samples $R_{i}=I(i\leq n)$ or sampling without replacement for a deterministic sequence n.

To properly define the causally interpretable ATE, we adopt the typical counterfactual outcome framework and its standard assumptions (Imbens and Rubin, 2015; Hernan and Robins, 2023). Let $Y^{\left(a\right)}$ be the counterfactual outcome with treatment set as a, for a∈{0, 1}. The ATE is defined as

(2) $$\Delta_{*}=E\;\left(Y^{\left(1\right)}-Y^{\left(0\right)}\right).$$

We make the following standard assumptions regarding the triplet (Y,A,X),

**Assumption 1** (*a*) *Consistency:* $Y=Y^{\left(A\right)}$;

(*b*) *(Causal inference) Positivity of treatment assignment*: 1/M≤P(A=1∣X)≤ 1-1/M *almost surely for an absolute constant* M<∞;

(*c*) *Ignorability*: $\left(Y^{\left(1\right)},Y^{\left(0\right)}\right)⫫A|X$.

The (causal inference) positivity in Assumption 1 is imposed on the treatment assignment A, which should be distinguished from the (missing data) positivity regarding the observation indicator R. Under the Assumption 1, the ATE can be alternatively expressed as

(3) $$\Delta_{*}=E\;\{E(Y|X,A=1)-E(Y|X,A=0)\}.$$

In the motivating EHR studies, $S_{i}$ represents the documentations and retrospective data curation of $\left(Y_{i},A_{i}\right)$, such as the presence of diagnosis code in follow-up for outcomes and medication codes at baseline for treatments, that are conceivably determined by the underlying truth $\left(Y_{i},A_{i}\right)$. In Figure 1, we present a setting such that the surrogates can be classified into those for $A_{i}$ and those for $Y_{i},S_{i}={\left(S_{i,a}^{⊤},S_{i,y}^{⊤}\right)}^{⊤}$. The causal identification (3) still holds with the introduction of additional variable $S_{i}$. Sometimes $S_{i}$ may contain colliders that are affected by both treatment $A_{i}$ and outcome $Y_{i},A_{i}\to S_{i}\leftarrow Y_{i}$, e.g. increased code counts from frequent healthcare visits as part of intense treatment or caused by poor outcome. Adjustment of colliders would distort causal relationship $A_{i}\to Y_{i}$ and should be excluded from causal identification (Hernan and Robins, 2023, Chapter 6.4). Throughout the paper, we assume MCAR (1) and Assumption 1.

**Remark 1** *We herein summarize the setting of our study*.

- Over a small randomly sampled labeled subset, we can causally identify ATE with outcome $Y_{i}$, binary treatment $A_{i}$ and confounders $X_{i}$ under standard consistency, (causal inference) positivity and ignorability assumptions.
- We seek to robustly enhance the efficiency of estimating ATE by incorporating the large unlabeled data containing confounders $X_{i}$ and surrogates $S_{i}$ without stringent model assumptions on $S_{i}$. The method should adaptively achieve
  - better efficiency if $S_{i}|X_{i}$ can effectively inform $\left(Y_{i},A_{i}\right)|X_{i}$;
  - the same property as the ATE estimated from labeled subset if $S_{i}|X_{i}$ cannot inform $\left(Y_{i},A_{i}\right)|X_{i}$.

## 3 SMMAL Estimation

We start by presenting in Section 3.1 the efficient influence function under the double missing SSL setting without assuming known model for unlabeled data. Deviating from the classical missing data setting, the derived efficient influence functions under double missing SSL setting have diverging variances, which requires a formal justification of its connection with efficiency lower bound in Sections 4.2. Our approach is hence distinguished from existing SSL literatures (Cheng et al., 2021; Kallus and Mao, 2024) that considered a simplified theoretical formulation to define the efficient influence function assuming known model for large unlabeled data. Then, we discuss the estimation of ATE with different ways of estimating the nuisance models involved in the efficient influence function in Section 3.2 for low-dimensional X and in Section 3.3 for high-dimensional X. As the standard tool to control over-fitting from using estimated models in subsequent estimation procedures (Lin and Ying, 1994; Chernozhukov et al., 2018; Newey and Robins, 2018; Hou et al., 2021b), cross-fitting is adopted for both settings, where we split the data into K (e.g. K=5) folds of approximately equal size. For k=1,…,K, we let ${ℐ}_{k}$ denote the index set for the kth fold of the data with size $N_{k}=\left|{ℐ}_{k}\right|$ and let ${ℐ}_{k}^{c}=\{1,\ldots ,N\}\setminus {ℐ}_{k}$, where |ℐ| denotes the cardinality of ℐ. Here, we do not split the folds separately for labeled and the unlabeled data because the label indicator $R_{i}$ is random under the MCAR formulation (1).

### 3.1 The efficient influence function

We define the following nuisance models:

$$\begin{array}{cccc} \text{PS}: & P\left(A=a|X\right)=\pi \left(a,X\right), & \text{OR}: & E\left(Y|A=a,X\right)=\mu \left(a,X\right),\\ \text{Imputations}: & P\left(A=a|W\right)=\Pi \left(a,W\right), & & E\left(Y|A=a,W\right)=m\left(a,W\right). \end{array}$$

We use the subscript star to indicate the true models, $\pi_{*},\mu_{*},\Pi_{*},m_{*}$. Starting from the efficient influence function with complete (cmp) observation of treatment and outcome (Robins et al., 1994; Kallus and Mao, 2024),

$$\phi_{\text{cmp}}\left(Y,A,X\right)=\mu_{*}\left(1,X\right)-\mu_{*}\left(0,X\right)+\frac{\text{I}\left(A=1\right)}{\pi_{*}\left(1,X\right)}\left\{Y-\mu_{*}\left(1,X\right)\right\}\;-\frac{\text{I}\left(A=0\right)}{\pi_{*}\left(0,X\right)}\left\{Y-\mu_{*}\left(0,X\right)\right\}-\Delta_{*},$$

we produced the efficient influence function through the following mapping

(4) $$\phi_{\text{SSL}}(RY,RA,W,R)\;=E\left\{\phi_{\text{cmp}}(Y,A,X)|W\right\}+\frac{R}{\rho_{N}}\left[\phi_{\text{cmp}}(Y,A,X)-E\left\{\phi_{\text{cmp}}(Y,A,X)|W\right\}\right]$$

(5) $$=\mu_{*}\left(1,X\right)+\frac{\Pi_{*}\left(1,W\right)}{\pi_{*}\left(1,X\right)}\left\{m_{*}\left(1,W\right)-\mu_{*}\left(1,X\right)\right\}\;-\mu_{*}\left(0,X\right)-\frac{\Pi_{*}\left(0,W\right)}{\pi_{*}\left(0,X\right)}\left\{m_{*}\left(0,W\right)-\mu_{*}\left(0,X\right)\right\}-\Delta_{*}\;+\frac{R\left\{\text{I}\left(A=1\right)Y-\text{I}\left(A=1\right)\mu_{*}\left(1,X\right)-\Pi_{*}\left(1,W\right)m_{*}\left(1,W\right)+\Pi_{*}\left(1,W\right)\mu_{*}\left(1,X\right)\right\}}{\rho_{\text{N}}\pi_{*}\left(1,X\right)}\;-\frac{R\left\{\text{I}\left(A=0\right)Y-\text{I}\left(A=0\right)\mu_{*}\left(0,X\right)-\Pi_{*}\left(0,W\right)m_{*}\left(0,W\right)+\Pi_{*}\left(0,W\right)\mu_{*}\left(0,X\right)\right\}}{\rho_{\text{N}}\pi_{*}\left(0,X\right)}.$$

In the formula (4) that produces $\phi_{\text{SSL}}$ from $\phi_{\text{cmp}},E\left\{\phi_{\text{cmp}}(Y,A,X)|W\right\}$ is the maximal information on ATE from the unlabeled data with a known imputation model, and the second term is the price for training the best imputation model over the labeled data. We provide the rigorous justification of this procedure in Section 4.

The efficient influence function in the missing data context is usually derived by projecting an arbitrary initial influence function to the nuisance tangent space (Tsiatis, 2007). The approach has been applied to the SSL setting with missing outcome by first deriving the efficient influence function under missing data setting and then setting $n/N≍\rho_{N}=0$ for the SSL setting with very large unlabeled data (Kallus and Mao, 2024). No formal justification of efficiency has been given in exiting literatures under the semi-supervised setting with $\rho_{N}\to 0$ yet $\rho_{N}>0$. Moreover, such standard procedure for deriving efficient influence function under missing data or causal inference settings is usually specific for the assumed dependence structure among variables, reflected by the correspondent chain-rule decomposition of nuisance model tangent space (Robins et al., 1994; Tsiatis, 2007; Kallus and Mao, 2024; Cheng et al., 2021). For estimating of ATE under SSL setting, existing formulation focused on the surrogates S that are defined as short-term markers for long-term outcomes Y, represented by the S→Y dependence pattern in causal diagram (Kallus and Mao, 2024; Cheng et al., 2021). The generalization to other types of surrogates S is currently absent. For example, surrogates S in our motivating EHR studies were imperfect documentations for treatment and outcome variables (A,Y), represented by the (A,Y)→S dependence pattern (Figure 1). The shift from S→Y to (A,Y)→S also creates the technical challenges in deriving projections to nuisance model tangent space defined according to the dependence pattern (see Section E2 of the Supplementary Materials).

While our derivation of efficient $\phi_{\text{SSL}}$ also involved projecting an inefficient $\left(R/\rho_{N}\right)\phi_{\text{cmp}}$, a common approach among existing literatures (Robins et al., 1994; Kallus and Mao, 2024), our approach did not impose stringent assumptions on surrogates S. To provide a general theoretical basis consistent across various surrogate mechanism, we established the connection between efficiency lower bound of complete data setting and that of double missing SSL setting through asymptotic local minimax result similar to Begun et al. (1983) in Section 4.2. Our efficiency lower bound justified projecting complete data efficient influence function $\phi_{\text{cmp}}$ to derive the SSL efficient influence function $\phi_{\text{SSL}}$. We further generalized the efficiency theory to other parameters with missing data under SSL setting in Section 7. Our alternative justification only requires 1) the target ATE parameter Δ can be identified by (Y,A,X) through $\phi_{\text{cmp}}$ and 2) S can provide information on (Y,A) when they are not observed over the unlabeled set. Hence, our framework covered a broad range of surrogate mechanism including both the setting considered by Kallus and Mao (2024) and the causal diagram in Figure 1.

### 3.2 SMMAL Procedure

Inspired by the double machine learning estimation (Chernozhukov et al., 2018) based on $\phi_{\text{cmp}}$, we propose the following SMMAL estimator for ATE:

1. For each labelled fold k, we estimate the nuisance models by the out-of-fold data ${ℐ}_{k}^{c}$, obtaining ${\hat{\pi }}^{\left(k\right)},{\hat{\mu }}^{\left(k\right)},{\hat{\Pi }}^{\left(k\right)},{\hat{m}}^{\left(k\right)}$;
2. Construct the estimated influence functions
$${\hat{𝒱}}_{\text{ik}}={\hat{\mu }}^{\left(k\right)}\left(1,X_{i}\right)+\frac{{\hat{\Pi }}^{\left(k\right)}\left(1,W_{i}\right)}{{\hat{\pi }}^{\left(k\right)}\left(1,X_{i}\right)}\left\{{\hat{m}}^{\left(k\right)}\left(1,W_{i}\right)-{\hat{\mu }}^{\left(k\right)}\left(1,X_{i}\right)\right\}\;-{\hat{\mu }}^{\left(k\right)}\left(0,X_{i}\right)-\frac{{\hat{\Pi }}^{\left(k\right)}\left(0,W_{i}\right)}{{\hat{\pi }}^{\left(k\right)}\left(0,X_{i}\right)}\left\{{\hat{m}}^{\left(k\right)}\left(0,W_{i}\right)-{\hat{\mu }}^{\left(k\right)}\left(0,X_{i}\right)\right\}\;+\frac{R_{i}\left\{A_{i}Y_{i}-A_{i}{\hat{\mu }}^{\left(k\right)}\left(1,X_{i}\right)\right\}}{\rho_{N}{\hat{\pi }}^{\left(k\right)}\left(1,X_{i}\right)}-\frac{R_{i}\left\{\left(1-A_{i}\right)Y_{i}-\left(1-A_{i}\right){\hat{\mu }}^{\left(k\right)}\left(0,X_{i}\right)\right\}}{\rho_{N}{\hat{\pi }}^{\left(k\right)}\left(0,X_{i}\right)}\;-\frac{R_{i}\left\{{\hat{\Pi }}^{\left(k\right)}\left(1,W_{i}\right){\hat{m}}^{\left(k\right)}\left(1,W_{i}\right)-{\hat{\Pi }}^{\left(k\right)}\left(1,W_{i}\right){\hat{\mu }}^{\left(k\right)}\left(1,X_{i}\right)\right\}}{\rho_{N}{\hat{\pi }}^{\left(k\right)}\left(1,X_{i}\right)}\;+\frac{R_{i}\left\{{\hat{\Pi }}^{\left(k\right)}\left(0,W_{i}\right){\hat{m}}^{\left(k\right)}\left(0,W_{i}\right)-{\hat{\Pi }}^{\left(k\right)}\left(0,W_{i}\right){\hat{\mu }}^{\left(k\right)}\left(0,X_{i}\right)\right\}}{\rho_{N}{\hat{\pi }}^{\left(k\right)}\left(0,X_{i}\right)}.$$
  and estimate the ATE by
(6) $${\hat{\Delta }}_{\text{SMMAL}}=\frac{1}{N}\sum_{k=1}^{K}\sum_{i\in {ℐ}_{k}}{\hat{𝒱}}_{\text{ik}}.$$
3. Estimate the asymptotic variance of $\sqrt{n}\left({\hat{\Delta }}_{\text{SMMAL}}-\Delta_{*}\right)$ by
(7) $${\hat{𝒱}}_{\text{SMMAL}}=\frac{\rho_{N}}{N}\sum_{k=1}^{K}\sum_{i\in {ℐ}_{k}}{\left({\hat{𝒱}}_{\text{ik}}-{\hat{\Delta }}_{\text{SMMAL}}\right)}^{2}.$$

Here we considered the $\sqrt{n}$ standardized estimation error $\sqrt{n}\left({\hat{\Delta }}_{\text{SMMAL}}-\Delta_{*}\right)$ instead of the $\sqrt{N}$ standardized estimation error $\sqrt{N}\left({\hat{\Delta }}_{\text{SMMAL}}-\Delta_{*}\right)$ because the latter is diverging at $\sqrt{N/n}≍\rho_{N}^{-1/2}$ rate due to the unbounded variance of $R_{i}/\rho_{N}$ as $\rho_{N}\to 0$. The (1-α)×100% confidence interval for ATE can be constructed with ${\hat{\Delta }}_{\text{SMMAL}}$ and ${\hat{𝒱}}_{\text{SMMAL}}$,

$$\left[{\hat{\Delta }}_{\text{SMMAL}}-{𝒵}_{\alpha /2}\sqrt{{\hat{𝒱}}_{\text{SMMAL}}/n},\;{\hat{\Delta }}_{\text{SMMAL}}+{𝒵}_{\alpha /2}\sqrt{{\hat{𝒱}}_{\text{SMMAL}}/n}\right]$$

where ${𝒵}_{\alpha /2}$ is the 1-α/2 quantile of standard normal distribution.

Similar to existing results in double machine learning literature, any estimators for the nuisance models with suitable rates of consistency can be used in our proposal as well. For low-dimensional W and smooth nuisance models, we can choose B-spline regression with proper order and degrees. Precise discussions on these rates, related conditions for general estimators and relevant smoothness classes for B-spline regression are listed in Section 4.2.

### 3.3 Doubly Robust SMMAL Construction in high-dimensions

We next discuss a specific construction of the SMMAL estimator when the dimensions p and q grow with n and p may be larger than n. In real-world evidence studies using EHRs, confounding adjustment often involves selection of the few determinants of treatment and risk factors for outcomes from a large number of candidate variables (Hou et al., 2021c, 2022). We focus on the binary Y and put the high-dimensional logistic regression models with link function $g(x)=1/\left(1+e^{-x}\right)$ on the nuisance models

(8) $$\pi \left(1,X\right)=g\left(\alpha^{⊤}X\right);\;\mu \left(a,X\right)=g\left(\beta_{a}^{⊤}X\right),\;a=0, 1;\;\Pi (1,W)=g\left(\xi^{⊤}W\right);\;m(a,W)=g\left(\zeta_{a}^{⊤}W\right),\;a=0, 1.$$

We denote the derivatives of the link g as $\dot{g}(x)=e^{x}/{\left(1+e^{x}\right)}^{2}$ and the corresponding loss function as $\ell (y,x)=\text{log}\left(1+e^{x}\right)-yx$. Other types of generalized linear models for OR model μ(a,X) may also be considered and derived similarly. To enhance the robustness against model mis-specification in π and μ, we propose a bias-reducing calibration after an initial estimation (Smucler et al., 2019). We added another layer of cross-fitting to reduce the overfitting bias when using initial estimators in the bias-reducing calibration. Compare with the general SMMAL algorithm in Section 3.2, the generic estimation process for nuisance models (Step 1 in Section 3.2) is expanded into the Step 1–4 of the following SMMAL algorithm for high-dimensional logistic regression. To ensure that estimated PS and OR are bounded away from zero and one, we propose to truncate linear predictors according to a predetermined constant M corresponding to a reasonable range for PS and OR probabilities, e.g. M=2.2 for range [0.1, 0.9]. Our algorithm for doubly robust SMMAL estimator ${\hat{\Delta }}_{\text{DR}}$ has the following steps:

1. For each labelled fold k, we estimate the imputation models by the Lasso over out-of-fold data ${ℐ}_{k}^{c}$,
(9) $${\hat{\xi }}^{\left(k\right)}=\underset{\xi \in R^{p+q+1}}{\text{argmin}}\frac{\sum_{i\in {ℐ}_{k}^{c}}R_{i}\ell \left(A_{i},\xi^{⊤}W_{i}\right)}{\sum_{i\in {ℐ}_{k}^{c}}R_{i}}+\lambda_{\eta }\|\xi {\|}_{1},\;\lambda_{\zeta }≍\sqrt{\text{log}(p+q)/n},\;{\hat{\zeta }}_{a}^{\left(k\right)}=\underset{\zeta \in R^{p+q+1}}{\text{argmin}}\frac{\sum_{i\in {ℐ}_{k}^{c}}I\left(A_{i}=a\right)R_{i}\ell \left(Y_{i},\zeta^{⊤}W_{i}\right)}{\sum_{i\in {ℐ}_{k}^{c}}I\left(A_{i}=a\right)R_{i}}+\lambda_{\zeta }\|\zeta {\|}_{1},\lambda_{\zeta }≍\sqrt{\text{log}(p+q)/n};$$
  Choice of the imputation method is flexible (See Remark 10).
2. For each labelled fold pair $\left(k_{1},k_{2}\right)$, we estimate the initial PS and OR models by the Lasso over out-of-two-folds data ${ℐ}_{k_{1},k_{2}}^{c}={\left({ℐ}_{k_{1}}\cup {ℐ}_{k_{2}}\right)}^{c}$,
(10) $${\hat{\alpha }}_{\text{init}}^{\left(k_{1},k_{2}\right)}=\underset{\alpha \in R^{p+1}}{\text{argmin}}\frac{\sum_{i\in {ℐ}_{k_{1},k_{2}}^{c}}R_{i}\ell \left(A_{i},\alpha^{⊤}X_{i}\right)}{\sum_{i\in {ℐ}_{k_{1},k_{2}}^{c}}R_{i}}+\lambda_{\alpha ,\text{init}}\|\alpha {\|}_{1},\;{\hat{\beta }}_{a,\text{init}}^{\left(k_{1},k_{2}\right)}=\underset{\beta \in R^{p+1}}{\text{argmin}}\frac{\sum_{i\in {ℐ}_{k_{1},k_{2}}^{c}}I\left(A_{i}=a\right)R_{i}\ell \left(Y_{i},\beta^{⊤}X_{i}\right)}{\sum_{i\in {ℐ}_{k_{1},k_{2}}^{c}}I\left(A_{i}=a\right)R_{i}}+\lambda_{\beta ,a,\text{init}}\|\beta {\|}_{1},$$
  with $\lambda_{\alpha ,\text{init}},\lambda_{\beta ,\text{a},\text{init}}≍\sqrt{\text{log}(p)/n}$;
3. Define the truncation at $2M,\tau \left(x\right)=\text{sign}\left(x\right)\text{min}\{|x|,2M\}$, and its composition with functions ${\dot{g}}_{\tau }(x)=\dot{g}(\tau (x))$ and ${e\text{xp}}_{\tau }(x)=\text{exp}\;(\tau (x))$. For each labelled fold $k_{1}$, we construct the calibrated losses,
(11) $$\ell_{\alpha ,a}\left(A,\alpha^{⊤}X;\beta \right)={\dot{g}}_{\tau }\left(X^{⊤}\beta \right)\left\{(a-A)\alpha^{⊤}X+I(A=a)e^{(-1{)}^{a}\alpha^{⊤}X}\right\},\;\ell_{\beta ,a}\left(Y,\beta^{⊤}X;\alpha \right)={\text{exp}}_{\tau }\left\{(-1{)}^{a}\alpha^{⊤}X\right\}\ell \left(Y_{i},\beta^{⊤}X_{i}\right),$$
  and estimate the PS and OR models by cross-fitting within out-of-fold data ${ℐ}_{k_{1}}^{c}$,
(12) $${\hat{\alpha }}_{a}^{\left(k_{1}\right)}=\underset{\alpha \in R^{p+1}}{\text{argmin}}\sum_{k_{2}\neq k_{1}}\sum_{i\in {ℐ}_{k_{2}}}\frac{R_{i}}{n}\ell_{\alpha ,a}\left(A,\alpha^{⊤}X_{i};{\hat{\beta }}_{a,\text{init}}^{\left(k_{1},k_{2}\right)}\right)+\lambda_{\alpha ,a}\|\alpha {\|}_{1},\;{\hat{\beta }}_{a}^{\left(k_{1}\right)}=\underset{\beta \in R^{p+1}}{\text{argmin}}\frac{\sum_{k_{2}\neq k_{1}}\sum_{i\in {ℐ}_{k_{2}}}I\left(A_{i}=a\right)R_{i}\ell_{\beta ,a}\left(Y_{i},\beta^{⊤}X_{i};{\hat{\alpha }}_{\text{init}}^{\left(k_{1},k_{2}\right)}\right)}{\sum_{i\in {ℐ}_{k_{1}}^{c}}I\left(A_{i}=a\right)R_{i}}+\lambda_{\beta ,a}\|\beta {\|}_{1},$$
  with $\lambda_{\alpha ,a},\lambda_{\beta ,a}≍\sqrt{\text{log}(p)/n}$.
4. Construct the nuisance model estimators:
(13) $${\hat{\pi }}^{\left(k\right)}\left(1,X_{i}\right)=g_{\tau }\left(X_{i}^{⊤}{\hat{\alpha }}_{1}^{\left(k\right)}\right),\;{\hat{\pi }}^{\left(k\right)}\left(0,X_{i}\right)=g_{\tau }\left(-X_{i}^{⊤}{\hat{\alpha }}_{0}^{\left(k\right)}\right),\;{\hat{\mu }}^{\left(k\right)}\left(a,X_{i}\right)=g\left(X_{i}^{⊤}{\hat{\beta }}_{a}^{\left(k\right)}\right),\;{\hat{\Pi }}^{\left(k\right)}\left(a,W_{i}\right)=g\left(W_{i}^{⊤}{\hat{\xi }}^{\left(k\right)}\right),\;{\hat{m}}^{\left(k\right)}\left(a,W_{i}\right)=g\left(W_{i}^{⊤}{\hat{\zeta }}_{a}^{\left(k\right)}\right);$$
5. Estimate the ATE by sending (13) to (6), producing ${\hat{\Delta }}_{\text{DR}}$.
6. Estimate the variance by sending (13) and ${\hat{\Delta }}_{\text{DR}}$ to (7), producing ${\hat{𝒱}}_{\text{DR}}$.

The (1-α)×100% confidence interval for ATE can be constructed with ${\hat{\Delta }}_{\text{DR}}$ and ${\hat{𝒱}}_{\text{DR}}$,

$$\left[{\hat{\Delta }}_{\text{DR}}-{𝒵}_{\alpha /2}\sqrt{{\hat{𝒱}}_{\text{DR}}/n},\;{\hat{\Delta }}_{\text{DR}}+{𝒵}_{\alpha /2}\sqrt{{\hat{𝒱}}_{\text{DR}}/n}\right]$$

where ${𝒵}_{\alpha /2}$ is the 1-α/2 quantile of standard normal distribution.

The calibrated losses (11) aim to estimate OR and PS models by approximately solving the equations of the partial derivatives of ${\hat{\Delta }}_{\text{DR}}$ with respect to PS and OR models being zero (Tan, 2020; Smucler et al., 2019). The correctly specified model will be recovered as it can be identified by the same equation. Even with mis-specified model, Δ will be insensitive to estimation errors in OR and PS models, guaranteed by the small partial derivatives. The property is referred to as the *Neyman orthogonality* (Chernozhukov et al., 2018), which produces $\sqrt{n}$ asymptotic normal estimator with sub $\sqrt{n}$ rate nuisance model estimations. We didn’t use imputations to improve estimation of OR and PS models because there is no asymptotic efficiency gain due to Neyman orthogonality but potential risk of introducing bias.

To control the overfitting bias from the sequential estimation process with 3 steps $\left({\hat{\alpha }}_{\text{init}},{\hat{\beta }}_{\text{a,init}}\right)\to \left({\hat{\alpha }}_{a},{\hat{\beta }}_{a}\right)\to {\hat{\Delta }}_{\text{DR}}$, we propose the two-level cross-fitting for learning ATE in (10) and (12), previously considered for semi-supervised learning of high-dimensional regression in (Hou et al., 2021b). The two-level cross-fitting has the advantage of having larger training set for each Lasso (using k-2 folds) compared to the averaging after data splitting (using (k-1)/2 folds) in Smucler et al. (2019). If we choose K=10, we are able to use at least 80% data while the data splitting in Smucler et al. (2019) may only use 45% data. Larger training sample typically allows the choice of smaller penalty factor thus reducing the bias. Taking averaging after data splitting, however, cannot reduce bias.

The truncation τ at M in (12) secured the (causal inference) positivity property of the initially estimated models with no compromise in estimation accuracy. Truncation of PS has been commonly invoked in practice when (causal inference) positivity holds in principle but is violated practically by estimated PS (Petersen et al., 2012; Ju et al., 2019). Our method generalized the truncation to OR prediction for binary outcome and identified a novel theoretical property of relaxing sparsity requirement for initial Lasso with the truncation. When the initial estimated model $X_{i}^{⊤}{\hat{\alpha }}_{\text{init}}$ is consistent for true models satisfying the (causal inference) positivity conditions, i. e. $X_{i}^{⊤}\alpha_{*}$ such that $0<g(-M)\leq g\left(X_{i}^{⊤}\alpha_{*}\right)\leq g(M)<\infty$, truncation at M brings the $X_{i}^{⊤}{\hat{\alpha }}_{\text{init}}$ closer to $X_{i}^{⊤}\alpha_{*}$ (See Lemma A20 in Supplementary Materials). Otherwise, the truncation always ensure $e\text{xp}\;(-2M)\leq {\text{exp}}_{\tau }\left(-X_{i}^{⊤}{\hat{\alpha }}_{\text{init}}\right)\leq \text{exp}\;(2M)$. Then, estimating calibrated OR coefficients ${\hat{\beta }}_{a}^{\left(k_{1}\right)}$ is a weighted $L_{1}$ penalized regression with bound weights independent of the responses, which we have shown to be consistent under mild assumptions (see Section D1 in Supplementary Materials). Same argument applies to truncation of $X_{i}^{⊤}{\hat{\beta }}_{a,\text{init}}$. Besides numerical stability, we can remove the sparsity condition associated with the initial estimator of the mis-specified model.

## 4 Theoretical Properties of the SMMAL

We established the $\sqrt{n}$-consistency of ${\hat{\Delta }}_{\text{SMMAL}}$ and the honest asymptotic coverage of the confidence intervals with consistent estimation of PS and OR models in Section 4.1. In Section 4.2, we derived the asymptotic distribution of the SMMAL estimator ${\hat{\Delta }}_{\text{SMMAL}}$ and the subsequent matching lower bound to show its semi-parametric efficiency in the low-dimensional W case while using B -spline series estimators for nuisance regression models. For high-dimensional sub-Gaussian X and W and sparse nuisance models, we demonstrated in Section 4.3 that ${\hat{\Delta }}_{\text{DR}}$ is *adaptively* sparsity/model doubly robust with sparse nuisance models (Rotnitzky et al., 2020; Smucler et al., 2019): sparsity doubly robust when both OR and PS are correctly specified; model doubly robust when one of OR or PS is correctly specified.

### 4.1 $\sqrt{n}$-inference

We require the following assumptions for nuisance models and the machine-learning estimators. We denote the true propensity score as $\pi_{*}(X)=E(A|X)$ and outcome regression as $\mu_{*}(a,X)=E(Y|X,A=a)$. As we do not require consistency of imputation models, we denote $\overset{‾}{\Pi }$ and $\overset{‾}{m}$ as the potentially biased asymptotic limits of the estimated imputation models.

**Assumption 2** *For a fixed constant* M, *we assume*

- *(Bounded response) almost surely* ${\text{sup}}_{i=1,\ldots ,N}\left|Y_{i}\right|\leq M$;
- *(Causal Inference Positivity) almost surely* ${\text{sup}}_{i=1,\ldots ,N}{\text{sup}}_{a=0, 1}\;1/\pi_{*}\left(a,X_{i}\right)\leq M$;
- *(Bounded estimators) almost surely*
$$\underset{k=1,\ldots ,K}{\text{sup}}\;\underset{i\in {ℐ}_{k}}{\text{sup}}\;\underset{a=0, 1}{\text{sup}}\text{max}\;\left\{\left|1/{\hat{\pi }}^{\left(k\right)}\left(a,X_{i}\right)\right|,\left|{\hat{\mu }}^{\left(k\right)}\left(a,X_{i}\right)\right|,\left|{\hat{\Pi }}^{\left(k\right)}\left(a,W_{i}\right)\right|,\left|{\hat{m}}^{\left(k\right)}\left(a,W_{i}\right)\right|\right\}\leq M;$$
- *(Rate of estimation)*
$$\underset{k=1,\ldots ,K}{\text{sup}}{\left\|{\hat{\pi }}^{\left(k\right)}-\pi_{*}\right\|}_{2}+{\left\|{\hat{\mu }}^{\left(k\right)}-\mu_{*}\right\|}_{2}+{\left\|{\hat{\Pi }}^{\left(k\right)}-\overset{‾}{\Pi }\right\|}_{2}+{\left\|{\hat{m}}^{\left(k\right)}-\overset{‾}{m}\right\|}_{2}\;+\sqrt{n}{\left\|{\hat{\pi }}^{\left(k\right)}-\pi_{*}\right\|}_{2}{\left\|{\hat{\mu }}^{\left(k\right)}-\mu_{*}\right\|}_{2}=o_{p}(1)$$
  *for some* $\overset{‾}{\Pi }$ *and* $\overset{‾}{m}$ *satisfying* ${\text{sup}}_{i=1,\ldots ,N}{\text{sup}}_{a=0, 1}\;\text{max}\left\{\overset{‾}{\Pi }\left(a,W_{i}\right),\left|\overset{‾}{m}\left(a,W_{i}\right)\right|\right\}\leq M$, *where for two models* $h_{1}(a,W)$ *and* $h_{2}(a,W)$, *we define*
(14) $${\left\|h_{1}-h_{2}\right\|}_{2}=\underset{a\in \{0, 1\}}{\text{max}}\;\sqrt{E\left[{\left\{h_{1}(a,W)-h_{2}(a,W)\right\}}^{2}\right]}.$$
  *Here we use the* $\ell_{2}$-*norm notation because the mean squared error (MSE) thus defined correspond to the* $\ell_{2}$-*estimation error for model coefficients under parametric models*.
- *(Stable variance)*
$${𝒱}_{*}=\text{Var}\left[\frac{AY-A\mu_{*}(1,X)}{\pi_{*}(1,X)}-\frac{\left(1-A)Y-(1-A)\mu_{*}(0,X\right)}{\pi_{*}(0,X)}\right.\;-\frac{\left\{\overset{‾}{\Pi }(1,W)\overset{‾}{m}(1,W)-\overset{‾}{\Pi }(1,W)\mu_{*}(1,X)\right\}}{\pi_{*}(1,X)}\;\left.+\frac{\left\{\overset{‾}{\Pi }(0,W)\overset{‾}{m}(0,W)-\overset{‾}{\Pi }(0,W)\mu_{*}(0,W)\right\}}{\pi_{*}(0,X)}\right]\in [1/M,M].$$

We established the validity and asymptotic distribution of ${\hat{\Delta }}_{\text{SMMAL}}$ in the following theorem.

**Theorem 2** *Under Assumption 2*,

$$\sqrt{n/{\hat{𝒱}}_{\text{SMMAL}}}\left({\hat{\Delta }}_{\text{SMMAL}}-\Delta_{*}\right)⇝N\left(0, 1\right),$$

*where* “ ⇝ ” *denotes convergence in distribution*.

Assumption 2 a guarantees the boundedness of all nuisance models. When Y is binary, the models $\pi_{*},\mu_{*},\Pi_{*}$ and $m_{*}$ are all bounded by one. Assumption 2b is equivalent to the standard (causal inference) positivity condition $\pi_{*}\left(1,X_{i}\right)\in [1/M,1-1/M]$ as in Assumption 1b. Assumption 2c can be guaranteed by truncation of nuisance model estimators at M, which would not compromise the estimation accuracy under Assumptions 2a and 2b. Assumption 2e ensures the proper scaling of the asymptotic variance of ${\hat{\Delta }}_{\text{SMMAL}}$. As noted following (7), the term with the $R/\rho_{N}$ factor from labeled data in $\phi_{\text{SSL}}$ dominates its variance if $\rho_{N}\to 0$. The rate condition for the PS and OR models in Assumption 2d matches those for the double machine-learning estimator proposed in Chernozhukov et al. (2018) if applied to the complete data subset of size n. Under MCAR by design, the missing data mechanism is known a priori, which we utilized to accommodate the mis-specified imputation models estimated at an arbitrarily slow rate.

**Remark 3** *Compared to existing work on semi-supervised estimation of ATE* (Cheng et al., 2021; Kallus and Mao, 2024) *approximating the* $\rho_{N}\to 0$ *setting by the* $\rho_{N}=0$ *setting, our SMMAL incorporates additionally the uncertainty from large yet finite unlabeled data through* (5)–(7). *As the result, the inference from SMMAL has two methodological advantages. First, by harmonizing the* n≪N *and* n≍N *settings, users may use the same SMMAL procedure without choosing from two setting-specific approaches* (Kallus and Mao, 2024). *Especially, it seems implausible to decide the asymptotic limit of* $\rho_{N}$ *by a single realization of the data. Second, the uncertainty of* ${\hat{\Delta }}_{\text{SMMAL}}$ *consists of the uncertainty from labeled data and the uncertainty from large but finite unlabeled data*,

$${𝒱}_{\text{SMMAL}}=\underset{{𝒱}_{L}\;\text{from}\;\text{labeled}\;\text{set}}{\underbrace{\text{Var}\left[\phi_{\text{cmp}}(Y,A,X)-\left\{\phi_{\text{cmp}}(Y,A,X)|W\right\}\right]}}+\underset{{𝒱}_{U}\;\text{from}\;\text{labeled}\;\text{set}}{\underbrace{\rho_{N}\text{Var}\left\{\phi_{\text{cmp}}(Y,A,X)|W\right\}}}.$$

*Unlike existing work* (Cheng et al., 2021; Kallus and Mao, 2024) *that only considered* ${𝒱}_{L}$ *from labeled set, our SMMAL variance estimation captures both* ${𝒱}_{L}$ *and* ${𝒱}_{U}$ *by involving estimated influence functions* ${\hat{𝒱}}_{\text{ik}}$ *for all observations so that SMMAL is expected to have less issues in underestimation of uncertainty particularly from unlabeled data with a moderately small* $\rho_{N}$ *in practice*.

### 4.2 Semi-parametric efficiency with low-dimensional confounder

We next formally establish the semi-parametric efficiency lower bound under the double missing SSL setting. Consider the non-parametric model for (W,RA,RY,R)

$${𝒮}_{\text{SSL}}=\left\{d{\text{P}}_{f}\left(w,a,y,r\right)={\left\{\rho_{\text{N}}f\left(w,a,y\right)\right\}}^{r}{\left\{\left(1-\rho_{\text{N}}\right)f_{w}\left(w\right)\right\}}^{\left(1-r\right)}d\nu_{\text{SSL}}\left(w,a,y,r\right):\right.\;\left.f\;\text{is}\;\text{density}\;\text{over}\;𝒲⊗\left\{0, 1\right\}⊗𝒴,\;\text{and}\;f_{w}\left(w\right)=\sum_{a\in \left\{0, 1\right\}}\int_{y\in 𝒴}f\left(w,a,y\right)d\nu_{y}\left(y\right)\right\}$$

for some measures $\nu_{y}$ over $𝒴,\nu_{w}$ over 𝒲 and

$$\nu_{\text{SSL}}(w,a,y,r)=\left(\nu_{w}\times \delta_{\left\{0, 1\right\}}\times \nu_{y}\right)(w,a,y)\times \delta_{1}(r)+\nu_{w}(w)\times \delta_{0}(r)$$

where $\delta_{𝒜}$ is the counting/Dirac measure over the set 𝒜. Elements in ${𝒮}_{\text{SSL}}$ can be indexed by the density f, and we denote the true density as $f_{*}$ and the true model $P_{f_{*}}$.

**Remark 4** *In existing work on ATE* (Robins et al., 1994; Kallus and Mao, 2024), *the model* A∣X *provides no information on the* Y∣A,X, *and thus would not be included in the nuisance tangent space. In our setting, however, the surrogates* S *induced a correlation between subspaces corresponding to* A∣X *and* Y∣A,X *in the nuisance tangent space, which indicates that* A∣X *provides information on the* Y∣A,X *through the unlabelled data. As the result, the geometry of the model tangent space is more complex, and the projection can no longer be obtained through simple conditional expectation. See* Section E2 *of the* Supplementary Materials *for details*.

We denote the total variation norm as $\|\cdot {\|}_{\text{TV}}$. In the following theorem, we establish the semi-parametric efficiency lower bound for Δ under ${𝒮}_{\text{SSL}}$ in the form of a local minimax theorem obtained in the spirit of Begun et al. (1983).

**Theorem 5** *Under Assumptions 2a, 2c and 2e, we have*

$$\underset{c\to \infty }{\text{lim}\;\text{inf}}\underset{N\to \infty }{\text{lim}\;\text{inf}}\underset{\hat{\Delta }}{\text{inf}}\underset{{\left\|f-f_{*}\right\|}_{\text{TV}}\leq c/\sqrt{\rho_{N}N}}{\text{sup}}\frac{\int N{\left(\hat{\Delta }-\Delta_{*}\right)}^{2}d\prod_{i=1}^{N}P_{f}\left(w_{i},a_{i},y_{i},r_{i}\right)}{\text{Var}\left\{\phi_{\text{SSL}}(\text{RY},\text{RA},W,R)\right\}}\geq 1.$$

**Remark 6** *Theorem 5 offers one example that the semi-parametric efficiency bound (SEB) derived under the classical missing data setting can be generalized to the double missing SSL setting with* $\rho_{N}\to 0$ *while* $\rho_{N}N\to \infty$. *Later in* Section 7, *we present Theorem 13 for general SSL setting (including specifically the double missing SSL setting). Previous attempts to formalize semi-parametric efficiency in a the SSL settings have assumed that the entire distribution of* W *is known, i.e*. N=∞ *and* $\rho_{N}=0$. *Under the simplified SSL setting with* N=∞, *the SEB can be derived by straightforward applications of standard results in classical semiparametric literature – see e.g.* van der Vaart (1998). *Indeed, another possible consideration for choosing this simplified formulation version is the ambiguity of defining regular estimators without (missing data) positivity assumption and thereby formalizing efficiency through the calibration of the best regular estimator. We bypassed this conceptual difficulty by providing the alternative characterization based on local asymptotic minimax theory – which may operate on all possible estimators instead of restricting to the class of regular procedures*.

Utilizing the correlation structure induced by the projection

$$\text{Cov}\left(E\left\{\phi_{\text{cmp}}(Y,A,X)|W\right\},R/\rho_{N}\left[\phi_{\text{cmp}}(Y,A,X)-E\left\{\phi_{\text{cmp}}(Y,A,X)|W\right\}\right]\right)=0,\;\text{Cov}\left[\phi_{\text{cmp}}(Y,A,X),E\left\{\phi_{\text{cmp}}(Y,A,X)|W\right\}\right]=\text{Var}\left[E\left\{\phi_{\text{cmp}}(Y,A,X)|W\right\}\right],$$

we obtain the limiting lower bound in Theorem 5 when $\rho_{N}\to 0$ that matches the asymptotic variance of the labeled data component in $\phi_{\text{SSL}}$,

$$\underset{\rho_{N}\to 0}{\text{lim}}\rho_{N}\text{Var}\left\{\phi_{\text{SSL}}(\text{RY},\text{RA},W,R)\right\}\;=\underset{\rho_{N}\to 0}{\text{lim}}\rho_{N}\text{Var}\left(E\left\{\phi_{\text{cmp}}(Y,A,X)|W\right\}+R/\rho_{N}\left[\phi_{\text{cmp}}(Y,A,X)-E\left\{\phi_{\text{cmp}}(Y,A,X)|W\right\}\right]\right)\;=\underset{\rho_{N}\to 0}{\text{lim}}\underset{\to 0}{\underbrace{\rho_{N}\text{Var}\left[E\left\{\phi_{\text{cmp}}(Y,A,X)|W\right\}\right]}}+\rho_{N}\text{Var}\left(R/\rho_{N}\left[\phi_{\text{cmp}}(Y,A,X)-E\left\{\phi_{\text{cmp}}(Y,A,X)|W\right\}\right]\right)\;+2\rho_{N}\underset{=0}{\underbrace{\text{Cov}\left(E\left\{\phi_{\text{cmp}}(Y,A,X)|W\right\},R/\rho_{N}\left[\phi_{\text{cmp}}(Y,A,X)-E\left\{\phi_{\text{cmp}}(Y,A,X)|W\right\}\right]\right)}}\;=\text{Var}\left[\phi_{\text{cmp}}(Y,A,X)-E\left\{\phi_{\text{cmp}}(Y,A,X)|W\right\}\right]\;=\text{Var}\left\{\phi_{\text{cmp}}(Y,A,X)\right\}+\text{Var}\left[E\left\{\phi_{\text{cmp}}(Y,A,X)|W\right\}\right]\;-2\underset{=\text{Var}\left[E\left\{\phi_{\text{cmp}}(Y,A,X)|W\right\}\right]}{\underbrace{\text{Cov}\left[\phi_{\text{cmp}}(Y,A,X),E\left\{\phi_{\text{cmp}}(Y,A,X)|W\right\}\right]}}\;=\text{Var}\left\{\phi_{\text{cmp}}(Y,A,X)\right\}-\text{Var}\left[E\left\{\phi_{\text{cmp}}\left(Y,A,X\right)|W\right\}\right].$$

From the representation above, we showed that the efficiency gain from the unlabeled data with surrogates is given by the variance of the $\phi_{\text{cmp}}$ explained by the surrogates and confounders. The efficiency gain based on semi-parametric efficiency theory typically requires consistent estimation of nuisance models. Under mis-specified imputation models, there is no general guarantee on efficiency gain. In Discussion (Section 8), we offered efficient linear combination as the backup plan when quality of estimated nuisance models is in doubt.

The key idea of the proof is to construct the two-dimensional least favorable perturbation in an asymmetric neighborhood with different size in two directions. The first direction is proportional to $\phi_{\text{cmp}}(Y,A,X)-E\left\{\phi_{\text{cmp}}(Y,A,X)|W\right\}$ and of $\text{size}≍1/\sqrt{N\rho_{N}}≍1/\sqrt{n}$, which reflects the level of the information on $\Delta_{*}$ from the labels and should naturally scale with the number of expected labels. The second direction is proportional to $E\left\{\phi_{\text{cmp}}(Y,A,X)|W\right\}$ and of $\text{size}≍1/\sqrt{N}$, which reflects the level of the information on $\Delta_{*}$ from the unlabelled data and should scale with the total sample size. The design of the different scales ensured the tightness of log-likelihood ratio between the perturbed and the true models, which would otherwise be degenerating or diverging.

We next show that the lower bound is attained under low-dimensional smoothness class models for the nuisance functions and can be operationalized by feeding B-spline regressions to ${\hat{\Delta }}_{\text{SMMAL}}$. Suppose the confounders and surrogates are bounded continuous variables of fixed dimension, $W\in [-M,M{]}^{p+q},p+q<d≍1$. We measure the smoothness of the models by ℋ(f(·)) the Hölder class defined in Definition A22, Section E of the Supplementary Materials.

**Assumption 3** *For a fixed constant* M, *we assume*

- *(Bounded density) the density functions for* X *and* $W,f_{X}(x)$ *and* $f_{W}(w)$, *are bounded and bounded from zero*,
$$f_{X}(x)\in [1/M,M],\forall x\in [-M,M{]}^{p},\;f_{W}(w)\in [1/M,M],\forall w\in [-M,M{]}^{p+q};$$
- *(Smooth models) the smoothness of the nuisance models observe*
$$\frac{1}{1+ℋ\left(\pi_{*}(a,\cdot )\right)/p}+\frac{1}{1+ℋ\left(\mu_{*}(a,\cdot )\right)/p}<1,\;ℋ\left(\Pi_{*}(a,\cdot )\right)>0,\;ℋ\left(m_{*}(a,\cdot )\right)>0,$$
  *for* a=0, 1.

**Corollary 7** *Under Assumptions 2a, 2b, 2e and 3, we may choose B-spline regressions with order*

$$\kappa \geq \text{max}\left\{ℋ\left(\pi_{*}(a,\cdot )\right),\;ℋ\left(\mu_{*}(a,\cdot )\right),\;ℋ\left(\Pi_{*}(a,\cdot )\right),\;ℋ\left(m_{*}(a,\cdot )\right)\;:\;a=0, 1\right\}-1,$$

*degrees*

$$1/\pi (a,\cdot )\;:\;n^{\frac{1}{1+ℋ\left(\pi_{*}(a,\cdot )\right)/p}},\;\mu (a,\cdot )\;:\;n^{\frac{1}{1+ℋ\left(\mu_{*}(a,\cdot )\right)/p}},\;\Pi (a,\cdot )\;:\;n^{\frac{1}{1+ℋ\left(\Pi_{*}(a,\cdot )\right)/(p+q)}},\;m(a,\cdot )\;:\;n^{\frac{1}{1+ℋ\left(m_{*}(a,\cdot )\right)/(p+q)}}$$

*and truncation at* M *for* ${\hat{\Delta }}_{\text{SMMAL}}$ *to achieve*

$$\sqrt{n}\left({\hat{\Delta }}_{\text{SMMAL}}-\Delta_{*}\right)/\sqrt{\rho_{N}\text{Var}\left\{\phi_{\text{SSL}}(\text{RY},\text{RA},W,R)\right\}}⇝N(0, 1).$$

Corollary 7 is special case of Theorem 2 under smooth models estimated by standard non-parametric estimation. By Corollary 7, the asymptotic MSE of ${\hat{\Delta }}_{\text{SMMAL}}$ is $\rho_{\text{N}}\text{Var}\left\{\phi_{\text{SSL}}\right\}/n≍\text{Var}\left\{\phi_{\text{SSL}}\right\}/N$, matching the lower bound established in Theorem 5. Therefore, we have justified the semi-parametric efficiency of ${\hat{\Delta }}_{\text{SMMAL}}$. At the same time, the lower bound in Theorem 5 is the sharp semi-parametric efficiency bound for $\Delta_{*}$ under double missing SSL setting ${𝒮}_{\text{SSL}}$.

### 4.3 Doubly robustness with high-dimensional confounder

To describe the sparsity/model double robustness of ${\hat{\Delta }}_{\text{DR}}$, we define the asymptotic limits for Lasso estimators in (9)–(12) under potentially mis-specified models.

(15) $$\bar{\xi }=\underset{\xi \in R^{p+q+1}}{\text{argmin}}E\left\{\ell \left(A_{i},\xi^{⊤}W_{i}\right)\right\},\;{\bar{\zeta }}_{a}=\underset{\zeta \in R^{p+q+1}}{\text{argmin}}E\left\{I\left(A_{i}=a\right)\ell \left(Y_{i},\zeta^{⊤}W_{i}\right)\right\},\;{\bar{\alpha }}_{\text{init}}=\underset{\alpha \in R^{p+1}}{\text{argmin}}E\left\{\ell \left(A_{i},\alpha^{⊤}X_{i}\right)\right\},\;{\bar{\beta }}_{a,\text{init}}=\underset{\beta \in R^{p+1}}{\text{argmin}}E\left\{I\left(A_{i}=a\right)\ell \left(Y_{i},\beta^{⊤}X_{i}\right)\right\},\;{\bar{\alpha }}_{a}=\underset{\alpha \in R^{p+1}}{\text{argmin}}E\left[\dot{g}\left(X_{i}^{⊤}{\bar{\beta }}_{a,\text{init}}\right)\left\{\left(a-A_{i}\right)\alpha^{⊤}X_{i}+I\left(A_{i}=a\right)e^{(-1{)}^{a}\alpha^{⊤}X_{i}}\right\}\right],\;{\bar{\beta }}_{a}=\underset{\beta \in R^{p+1}}{\text{argmin}}E\left[e\text{xp}\left\{(-1{)}^{a}X_{i}^{⊤}{\bar{\alpha }}_{\text{init}}\right\}I\left(A_{i}=a\right)\ell \left(Y_{i},\beta^{⊤}X_{i}\right)\right],$$

We use $\|\cdot {\|}_{0}$ to denote the sparsity of a vector and $\|\cdot {\|}_{\psi_{2}}$ denote the sub-Gaussian norm for random variables or vectors. The sparsities of coefficients for OR ${\left\|\beta_{a}\right\|}_{0}$ and PS $\|\alpha {\|}_{0}$ models reflect the numbers of true determinants for the treatment and outcomes, including the true confounders that must be adjusted for. The detailed definition is given in Definition A23, Section E of the Supplementary Materials.

**Assumption 4** *For constant* M *independent of dimensions* n,N,p,q,

- *(Sub-Gaussian and bounded covariates) the vector of confounders and surrogates is sub-Gaussian,* ${\text{sup}}_{\|v{\|}_{2}=1}\;{\left\|v^{⊤}W\right\|}_{\psi_{2}}\leq M$, *and coordinate-wisely bounded* $\|W{\|}_{\infty }\leq M$ *almost surely*;
- *(Identifiability) the variance of* W *is invertible* ${\text{inf}}_{\|v{\|}_{2}=1}\;v^{⊤}\text{Var}(W)v\geq 1/M$;
- *(Causal Inference positivity) the true propensity scores and the asymptotic predictions of all models are bounded away from zero and one, almost surely*,
$$\pi_{*}(a,X)\in [1/M,1-1/M],\;\text{max}\;\left\{\left|{\bar{\xi }}^{⊤}W\right|,\left|{\bar{\zeta }}_{a}^{⊤}W\right|,\left|{\bar{\alpha }}_{a}^{⊤}X\right|,\left|{\bar{\beta }}_{a}^{⊤}X\right|\;:\;a=0, 1\right\}\leq M;$$
- *and one of the following:*
  - *(PS correct) the propensity model is correct*, $E(A|X)=g\left(\alpha_{*}^{⊤}X\right)$ *and the dimensions satisfy*
(16) $$\frac{\left({\left\|{\bar{\beta }}_{1}\right\|}_{0}+{\left\|{\bar{\beta }}_{0}\right\|}_{0}\right)\;\text{log}(p)+\left(\|\bar{\xi }{\|}_{0}+{\left\|{\bar{\zeta }}_{1}\right\|}_{0}+{\left\|{\bar{\zeta }}_{0}\right\|}_{0}\right)\;\text{log}(p+q)}{n}\;+{\left\|\alpha_{*}\right\|}_{0}\left({\left\|\alpha_{*}\right\|}_{0}+{\left\|{\bar{\beta }}_{1}\right\|}_{0}+{\left\|{\bar{\beta }}_{0}\right\|}_{0}\right)\;\text{log}(p{)}^{2}/n=o_{p}(1);$$
  - *(OR correct) the OR model is correct*, $E(Y|A=a,X)=g\left(\beta_{*,a}^{⊤}X\right)$ *and the dimensions satisfy*
(17) $$\frac{\left({\left\|{\bar{\alpha }}_{1}\right\|}_{0}+{\left\|{\bar{\alpha }}_{0}\right\|}_{0}\right)\;\text{log}(p)+\;\left(\|\bar{\xi }{\|}_{0}+{\left\|{\bar{\zeta }}_{1}\right\|}_{0}+{\left\|{\bar{\zeta }}_{0}\right\|}_{0}\right)\;\text{log}(p+q)}{n}\;+\sum_{a=0, 1}{\left\|\beta_{*,a}\right\|}_{0}\left({\left\|\beta_{*,a}\right\|}_{0}+{\left\|{\bar{\alpha }}_{a}\right\|}_{0}\right)\;\text{log}(p{)}^{2}/n=o_{p}(1);$$
  - *(both correct) both models are correct*, $E(A|X)=g\left(\alpha_{*}^{⊤}X\right)$ and E(Y∣A= $a,X)=g\left(\beta_{*,a}^{⊤}X\right)$ *and the dimensions satisfy*
(18) $$\frac{\left({\left\|\alpha_{*}\right\|}_{0}+{\left\|\beta_{*,1}\right\|}_{0}+{\left\|\beta_{*,0}\right\|}_{0}\right)\;\text{log}(p)+\left(\|\bar{\xi }{\|}_{0}+{\left\|{\bar{\zeta }}_{1}\right\|}_{0}+{\left\|{\bar{\zeta }}_{0}\right\|}_{0}\right)\;\text{log}(p+q)}{n}\;+{\left\|\alpha_{*}\right\|}_{0}\left({\left\|\beta_{*,1}\right\|}_{0}+{\left\|\beta_{*,0}\right\|}_{0}\right)\;\text{log}(p{)}^{2}/n=o_{p}(1);$$

**Theorem 8** *Under Assumption 4,* ${\hat{\Delta }}_{\text{DR}}$ *converges in distribution to a normal random variable at* $\sqrt{n}$-*rate*,

$$\sqrt{n/{\hat{𝒱}}_{\text{DR}}}\left({\hat{\Delta }}_{\text{DR}}-\Delta_{*}\right)⇝N(0, 1),$$

*where* “ ⇝ ” *denotes convergence in the distribution*.

Besides the double robustness toward PS and OR, ${\hat{\Delta }}_{\text{DR}}$ is additionally robust to the imputation models. Similar to the general Theorem 2, we utilized the known missing data mechanism under MCAR by design to allow model mis-specifications on the imputation.

**Remark 9** *Regarding the PS model and OR model estimated over the labeled data of size* n, *our* ${\hat{\Delta }}_{\text{DR}}$ *is both rate doubly robust* Rotnitzky et al. (2020) *and model doubly robust* (Smucler et al., 2019). *When both models are correct, the dimension condition* (18) *for the PS model and OR model in Assumption 4d-iii satisfies the condition for rate doubly robust, i.e. each sparsity obeying* ${\left\|\alpha_{*}\right\|}_{0}\ll n/\text{log}(p),{\left\|\beta_{*,a}\right\|}_{0}\ll n/\text{log}(p)$ *and their product satisfying* ${\left\|\alpha_{*}\right\|}_{0}{\left\|\beta_{*,a}\right\|}_{0}\ll n/\text{log}(p{)}^{2}$. *In the case of only one model is correct, our* ${\hat{\Delta }}_{\text{DR}}$ *can still provide $\sqrt{n}$*-*inference, thus being model doubly robust. By the truncation* τ *in* (12), *we are able to completely remove the sparsity requirement of the mis-specified initial model under the (causal inference) positivity condition of Assumption 4c. The general framework of* Smucler et al. (2019) *would require all models in* (15) *being sparse*.

**Remark 10** *As the correct model specification is only required for OR or PS in Assumption 4d, the validity of Theorem 8 does not rely on the consistency of imputation models based on the MCAR missing data mechanism by design. Therefore, the choice on imputation methods* (9) *can be flexible. If preliminary evidence suggests that certain element in* S *contains the most information such as* $S_{a}$ *for* A *and* $S_{y}$ *for* Y, *we can remove penalty for the associated coefficients or simply run the low-dimensional regressions* $A\sim S_{a}$ and $Y\sim S_{y}$.

**Remark 11** *We presented the theory according to the exact sparsity in Assumption 4d-iii for two considerations. First, the exact sparsity has a clear interpretation that classifies the covariates into relevant signals and irrelevant noises, about which domain experts may have a preliminary evaluation in applications. Second, the exact sparsity facilitates direct comparison with many related literatures have used exact sparsity to measure the local efficiency or robustness of their proposed methods* (Farrell, 2015; Tan, 2020; Smucler et al., 2019; Zhang et al., 2023). *The exact sparsity in Assumption 4d-iii can be substituted by other conditions that produce the appropriate estimation rate in the more general Assumption 2d. For example, estimation rates of* $L_{1}$ *penalized high-dimensional generalized linear models have been established for approximately sparse models* (Negahban et al., 2012; Smucler et al., 2019).

## 5 Simulation

We conducted extensive simulation studies to evaluate the finite sample performance of the SMMAL methods. Throughout the simulations, we set the total sample size N=10000, the number of labels n=500, the number of repeats as 1000, q=2 with one surrogate $S_{A}$ for A and another $S_{Y}$ for Y. We focused on the situation that Y is also binary. Let Φ be cumulative distribution function for standard normal distribution. The surrogates for binary A and Y were generated from mixture Beta distribution of the form:

$$\begin{array}{rr} & S_{A}=AS_{A,1}+\left(1-A\right)S_{A,0},S_{Y}=YS_{Y,1}+\left(1-Y\right)S_{Y,0},\\ \text{Low-dimensional}\;\text{model}: & S_{A,1}\sim \text{Beta}\left(\alpha_{A}+X,1\right),S_{A,0}\sim \text{Beta}\left(1,\alpha_{A}+X\right),\\ & S_{Y,1}\sim \text{Beta}\left(\alpha_{Y}+X,1\right),S_{Y,0}\sim \text{Beta}\left(1,\alpha_{Y}+X\right);\\ \text{High-dimensional}\;\text{model}: & S_{A,1}\sim \text{Beta}\left(\alpha_{A}+\Phi \left(X_{1}\right),1\right),S_{A,0}\sim \text{Beta}\left(1,\alpha_{A}+\Phi \left(X_{1}\right)\right),\\ & S_{Y,1}\sim \text{Beta}\left(\alpha_{Y}+\Phi \left(X_{1}\right),1\right),S_{Y,0}\sim \text{Beta}\left(1,\alpha_{Y}+\Phi \left(X_{1}\right)\right). \end{array}$$

The mixture Beta distribution mimicked the outputs from phenotyping algorithms, which typically take value between zero and one (Liao et al., 2019). We considered a list of values for $\alpha_{A}$ and $\alpha_{Y}$ (Table 1), corresponding to different level of prediction accuracy measured by area-under-curve (AUC) of the receiver operating characteristic (ROC). Five values were considered for $\alpha_{A}$ and $\alpha_{Y}$, creating 25 two-way combinations for each simulation setting.

We considered two scenarios for generating the data, the low-dimensional smooth model and high-dimensional logistic regression.

### Low-dimensional smooth model

**Figure 2:**
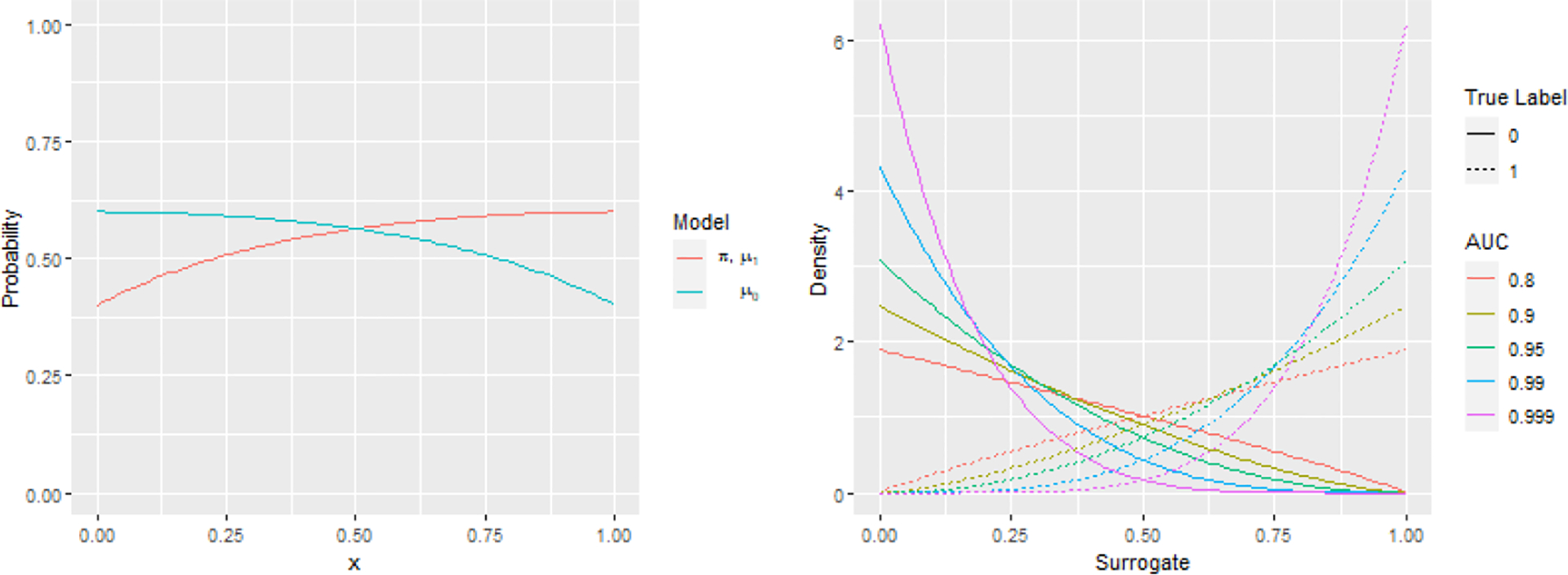
Visualized simulation settings. Left-the models for PS and OR under the low-dimensional setting. Right-the mixture Beta distribution for surrogates at different level of prediction accuracy (AUC 0.8, 0.9, 0.95, 0.99, 0.999) at the median covariate (X=0.5 under low-dimensional smooth model and $X_{1}=0$ under high-dimensional logistic regression).

We generated the one dimensional X∈R from Uniform (0,1) and set the PS and OR to be the following smooth models (Figure 2):

$$\pi_{*}(1,X)=\mu_{*}(1,X)=1-1.2/\left(3-X^{2}\right),\;\mu_{*}(0,X)=1-1.2/\left\{3-(1-X{)}^{2}\right\}.$$

We used tensor product first order B-spline (piece-wise linear splines) regression to estimate the nuisance models. The splines were constructed from bs function of the *splines* R package. The degrees were selected by 10 fold cross-validation among integers less than $\sqrt{n}\approx 22$ according to the out-of-fold entropy. Using the cross-fitted nuisance models from B-spline regression with K=10, we obtained point and interval estimates for the ATE based on ${\hat{\Delta }}_{\text{SMMAL}}$ and ${\hat{𝒱}}_{\text{SMMAL}}$. As the benchmark, we also estimated the ATE using the labeled data only by the double machine learning method (Chernozhukov et al., 2018).

### High-dimensional logistic regression

We generated the high-dimensional $X\in R^{p}$ with p=500 from the multivariate Gaussian distribution with auto-regressive correlation structure:

$$U_{1},\ldots ,U_{p}\overset{i.i.d.}{\sim }N\left(0, 1\right),\;X_{1}=U_{1},\;X_{j}=0.5X_{j-1}+\sqrt{0.75}U_{j}.$$

We generated A and Y from the high-dimensional logistic regression models

$$\text{PS}\;\text{Linear}\;:\pi_{*}(1,X)=g\left(0.5X_{1}+0.25X_{2}+0.125X_{3}\right);\;\text{PS}\;\text{Interaction}:\;\pi_{*}(1,X)=g\left\{\left(0.5X_{1}+0.25X_{2}+0.125X_{3}\right)\left(1+0.0625X_{1}+0.125X_{2}-0.5X_{3}\right)\right\};\;\text{OR}\;\text{Linear}:\;\mu_{*}(1,X)=g\left(0.1+0.25X_{1}+0.125X_{2}+0.0625X_{3}\right),\;\mu_{*}(0,X)=g\left(-0.1-0.25X_{1}-0.125X_{2}-0.0625X_{3}\right);\;\text{OR}\;\text{Interaction}:\;\mu_{*}(1,X)=g\left\{\left(0.1+0.25X_{1}+0.125X_{2}+0.0625X_{3}\right)\right.\;\left.\times \left(1+0.0625X_{1}+0.125X_{2}-0.5X_{3}\right)\right\},\;\mu_{*}(0,X)=g\left\{\left(-0.1-0.25X_{1}-0.125X_{2}-0.0625X_{3}\right)\right.\;\left.\times \left(1+0.0625X_{1}+0.125X_{2}-0.5X_{3}\right)\right\}.$$

As signal strength is known to impact variable selection in theory and practice (Fan and Peng, 2004; Fan and Lv, 2010), we set up the coefficients in the models to reflect different level of signal strength: 0.5-strong, 0.25-moderately strong, 0.125-moderately weak, 0.0625-weak. For PS/OR models with second order interactions, we still fitted high-dimensional logistic regression without interactions, creating the mis-specification scenarios. We considered 3 combinations corresponding to the three settings of Assumption 4d: correct models (PS Linear + OR Linear); mis-specified PS (PS Interaction + OR Linear); mis-specified OR (PS Linear + OR Interaction). We set the number of the folds as 10 and fitted the imputations (9) and initial estimators (10) using *glmnet* from R-package *glmnet*. We fitted the calibrated estimators (12) using rcal from from R-package *rcal*. The penalty parameters were selected by 10-fold cross validation with out-of-fold entropy. Using the cross-fitted nuisance models, we estimated the ATE using ${\hat{\Delta }}_{\text{DR}}$ and construct the 95% confidence interval based on the variance estimator ${\hat{𝒱}}_{\text{DR}}$. As the benchmark, we also estimated the ATE by the model doubly robust estimation (Smucler et al., 2019) using (1) the labeled data alone; (2) the dichotomized surrogates defined by

$${\tilde{Y}}_{i}=I\left(S_{Y,i}\geq 1-n^{-1}\sum_{i=1}^{N}R_{i}Y_{i}\right),\;{\tilde{A}}_{i}=I\left(S_{A,i}\geq 1-n^{-1}\sum_{i=1}^{N}R_{i}A_{i}\right).$$

We refer to the two benchmarks as supervised learning (SL) and unsupervised learning (UL).

### Results

**Figure 3:**
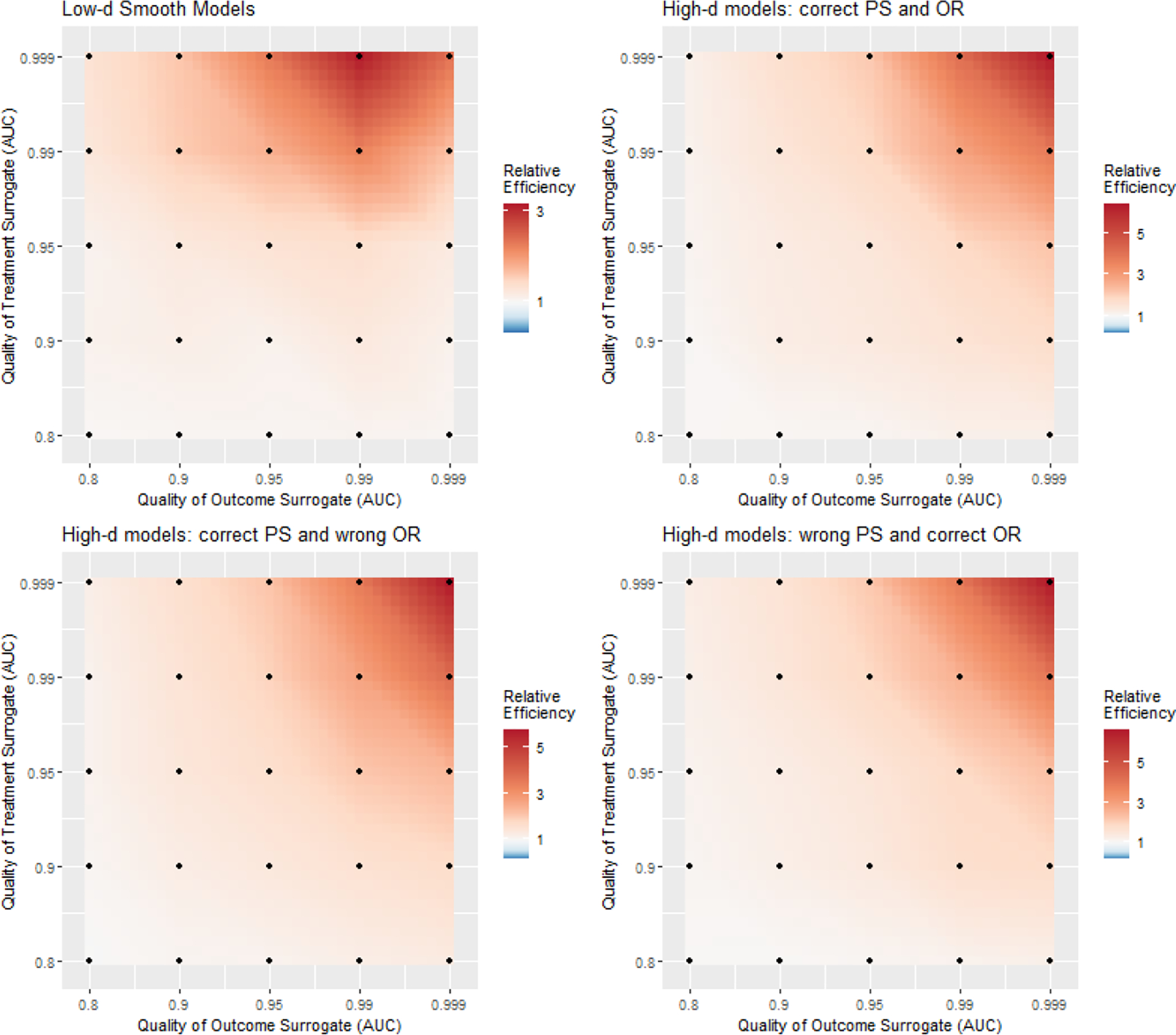
Heat map for relative efficiency of the SMMAL compared to the benchmark supervised learning in all four simulation settings. Deeper red indicates larger advantage of the semi-supervised estimation. We set relative efficiency one as white in all plots, but the scale varies between low-dimensional setting and high-dimensional settings.

**Figure 4:**
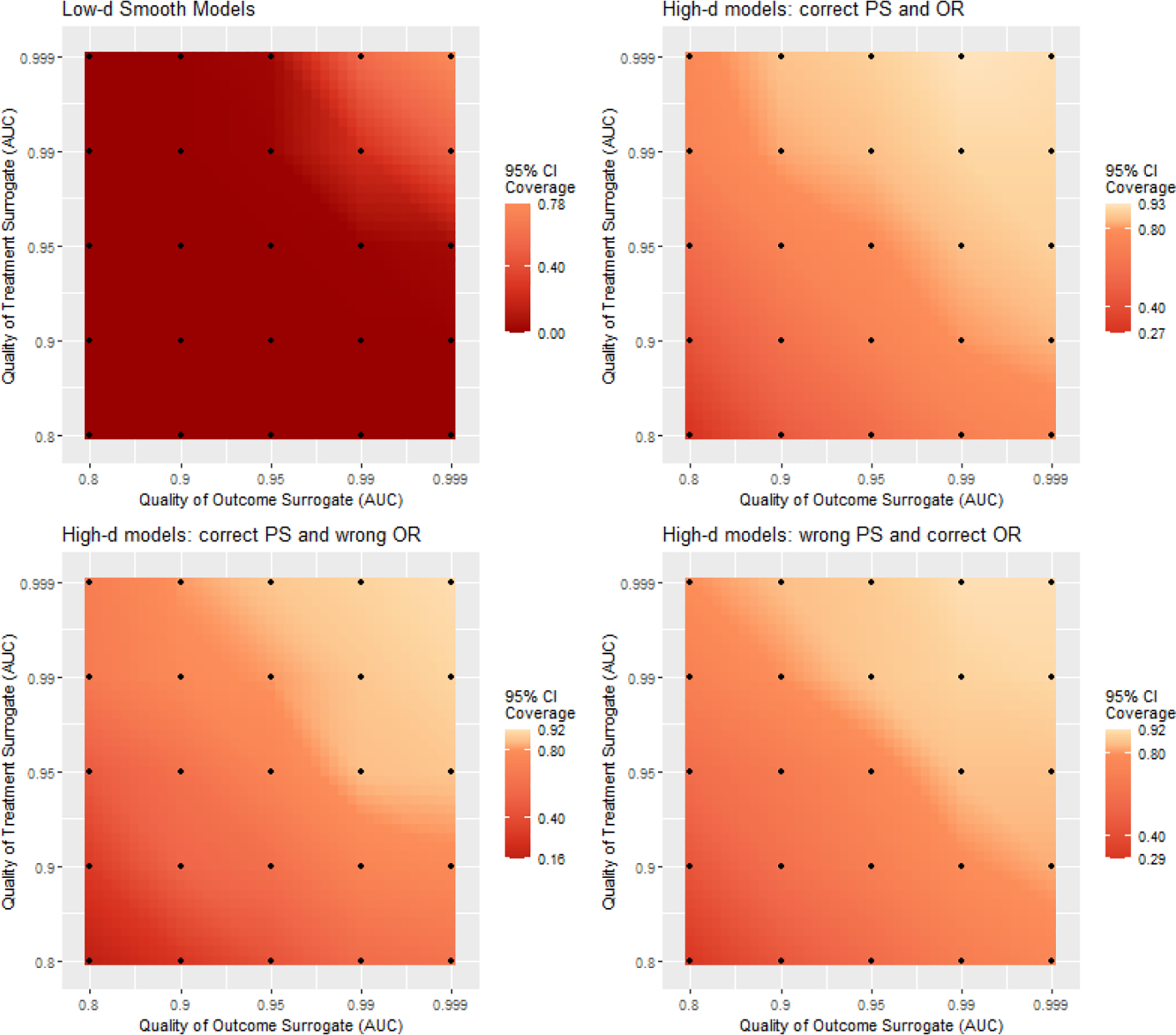
Heat map for coverage of 95% confidence intervals by unsupervised learning. White marks 0.95 coverage rate. Orange marks 0.8 coverage rate. Deeper red indicates poorer coverage rate by unsupervised learning.

Results generally followed a consistent pattern across low-d and high-d settings. Comparison between settings, however, is not meaningful due to the completely different data generating processes. In Figure 3, we visualized the relative efficiency of our semi-supervised ${\hat{\Delta }}_{\text{SMMAL}},{\hat{\Delta }}_{\text{DR}}$ compared to their supervised benchmarks. In general, our semi-supervised approaches gained efficiency from the unlabeled data whose magnitude was increasing with the minimal prediction accuracy of the two surrogates. With good imputation (AUC .95) from both surrogates, the relative efficiency was about 1.32–1.64 across all settings. With great imputation (AUC .99) from both surrogates, the relative efficiency was about 2.23–2.89 across all settings. The result quantified the benefit from improving the quality of surrogates in terms of relative increase in labels. Since the algorithms to curate surrogates are often portable to other studies sharing the variables, effort put into high-quality labels is more cost-effective compared to the brutal expansion in labeling. The detailed simulation results containing the bias, standard deviation, average standard error, coverage of 95% confidence interval for our semi-supervised ${\hat{\Delta }}_{\text{SMMAL}},{\hat{\Delta }}_{\text{DR}}$ along with those for the supervised benchmarks were presented in Tables A4–A7 in Section A of the Supplementary Materials. Our semi-supervised ${\hat{\Delta }}_{\text{SMMAL}},{\hat{\Delta }}_{\text{DR}}$ achieved reasonably honest inference with coverage of 95% confidence interval close to the nominal level. In Figure 4, we visualized the coverage of 95% confidence intervals by unsupervised learning. Using the dichotomized surrogates as if they were the true treatment and outcome led to under coverage of the confidence intervals even for nearly perfect surrogates, and the under coverage exacerbated with poorer surrogates. The detailed summaries on the bias, standard deviation and coverage of 95% confidence interval for the unsupervised benchmark were presented in Table A8 in Section A of the Supplementary Materials.

## 6 Real-world evidence on targeted cancer therapy

We applied the proposed SMMAL method to EHR data from Mass General Brigham healthcare to generate real-world evidence (RWE) on treatment effect of targeted therapy for metastatic colorectal cancer in comparison with conventional chemotherapy. Over the past two decades, a total of 9 targeted therapies have been approved for the treatment of colorectal cancer (Xie et al., 2020), the 4th most prevalent and lethal cancer (U.S. Cancer Statistics Working Group, 2022). While the targeted therapies have been reported as advantageous compared to conventional chemotherapy in clinical trials within specific trial populations, their effectiveness in real-world patient population has not been fully established. With increasing availability of EHR data, it is now plausible to generate RWE on targeted cancer therapy with respect to their efficacy in improving progression free survival via causal modeling treating EHR data as an observational cohort. Unfortunately, such a modeling task is highly challenging with EHR data due to the lack of readily available precise information on both treatments patient received and progression free survival. To overcome this challenge, we manually annotated treatment-response information for 100 randomly selected patients. We derived several potential surrogates for both $S_{A}$ and $S_{Y}$ from codified and narrative EHR data, which have varying degree of accuracy as shown in Table 2. Our goal was to leverage both the labeled observations on Y and S as well as the larger set of unlabeled EHR data to infer about ATE for targeted therapy based on SMMAL.

The full study cohort consisted of N=4147 colorectal cancer patients who have available cancer stage information extracted via a natural language process tool (Yuan et al., 2021) and received chemotherapy and/or targeted therapy. We grouped therapies into chemotherapy alone and targeted therapy which includes those treated with any of the 9 treatments: Bevacizumab, Cetuximab, Ipilimumab, Regorafenib, Pembrolizumab, Nivolumab, and Tipiracil. We set the outcome as 1-year progression free survival, a binary outcome defined as: 1 – exit in terminal condition (death/terminal care) or development of new metastasis site with 1-year from the treatment initiation; 0 – otherwise. As the standard quality control (Hou et al., 2023), an abstractor randomly sampled n=100 from the study cohort and annotated the gold-standard labels for prescription of targeted medication, terminal condition and new metastasis site by manually reviewing those patients’ EHR. The treatment A and Y outcome were defined based on annotations over the labeled set, creating the MCAR data. We reported the treatment and outcome labels as well as their EHR proxies in Table A9 of Supplementary Materials Section B, where we also described the construction of the reasonably good surrogates shown in Table 2.

We extracted a comprehensive list of potential confounders (Table 3). From EHR near the colorectal cancer diagnosis date, we used location specific colorectal cancer diagnosis code to identify the initial tumor location and natural language process tool (Yuan et al., 2021) to extract the initial stage. We also extracted the code for secondary malignancy at lymph node and other distant organs. From EHR between cancer diagnosis and subsequent metastasis, we extracted the codes for common procedures (chemotherapy, radiotherapy, colon biopsy and colon rescission). From EHR near the metastasis date, we used location specific secondary malignancy code to identify the initial metastasis site(s). We also adjusted for the time gap between diagnosis and metastasis, healthcare utilization before metastasis or one year before metastasis measured by days with diagnosis codes and the high-dimensional general health status consisting of diagnosis code counts grouped by the PheWAS catalog (Hou et al., 2022). The targeted therapy arm was associated with factors for poor prognosis including higher proportion of stage IV at diagnosis (81% vs 58%), higher proportion of likely liver metastasis (57% or 67% vs 34%). After merging rare levels for cancer characteristics at initial diagnosis (tumor location, cancer stage) and deleting features with fewer than 10 occurrence in labeled subset, we obtained the p=55 potential confounders.

**Figure 5:**
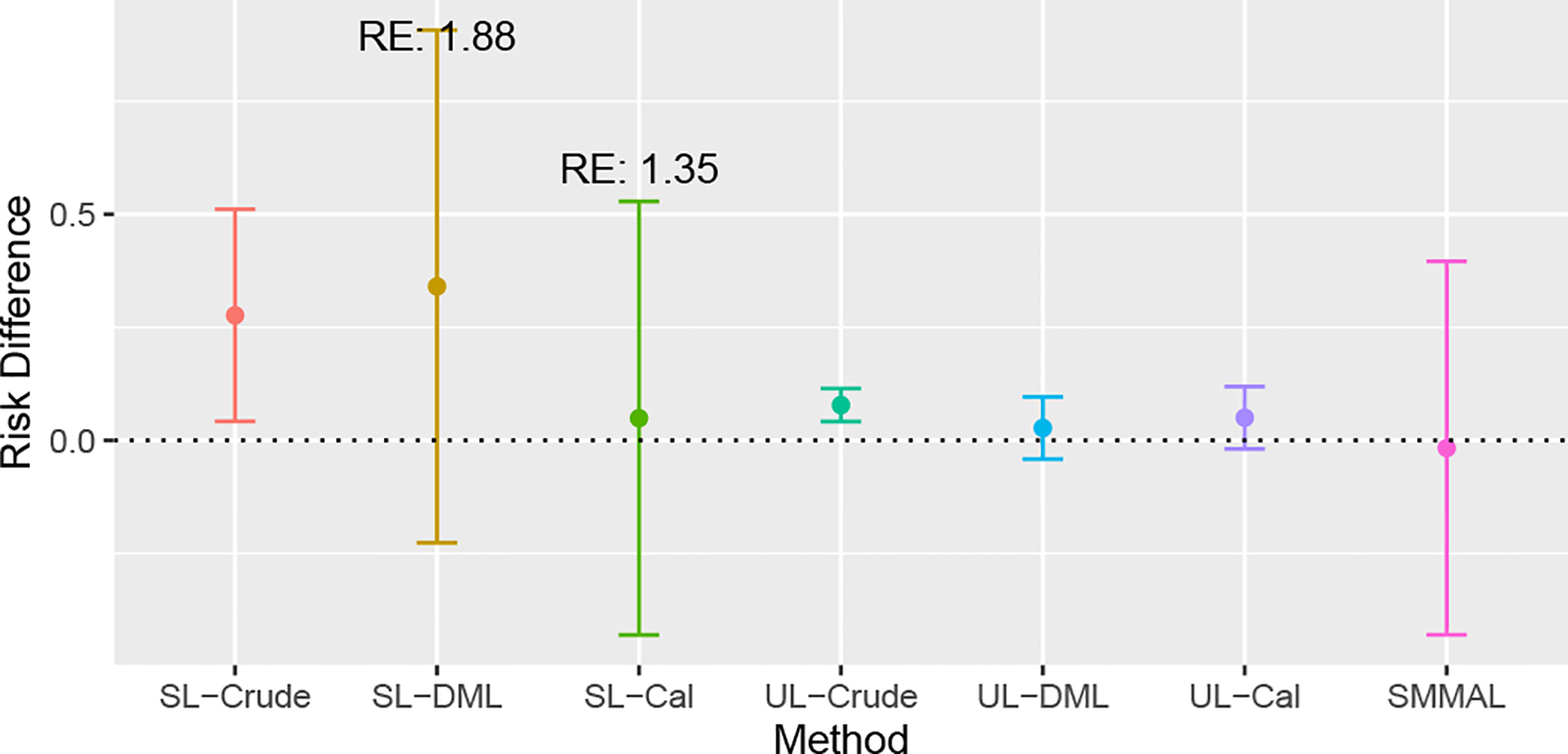
Point estimate and 95% confidence interval of average risk difference from crude, Double Machine-Learning (DML), calibrated (Cal) and SMMAL analyses. Supervised learning (SL) benchmark analysed only uses the labeled data. Unsupervised learning (UL) benchmark analyses used dichotomized surrogates by matching prevalence observed in labeled data. The RE value indicated the SMMAL’s relative efficiency in comparison with the two supervised benchmark methods (ratio of estimated variances).

We applied the doubly robust SMMAL in high-dimensions described in Section 3.3. Besides the crude analysis, we ran two benchmark analyses, the double machine learning (DML) (Chernozhukov et al., 2018) using initial estimators (10) and the calibrated estimation (Cal) (Tan, 2020; Smucler et al., 2019) using the calibrated estimators (12). Both supervised learning (SL) using labeled data only and the unsupervised learning (UL) deriving treatment and outcome from the dichotomized surrogates by matching observed prevalence in labeled data were considered. The number of fold was set as K=5, and the penalties factors were selected by the minimal cross-validated entropy. In Figure 5, we displayed the point estimation and the 95 % confidence interval. The confounder adjusted analysis results suggested that on average, targeted therapy had comparable efficacy compared to traditional chemotherapy. Compared to the SL crude analysis which indicated worse outcomes for targeted therapy, our SMMAL accounted for substantial confounding caused by association between target therapy and factors indicating poor prognosis. Except for the crude analysis that did not adjust for any confounding, our SMMAL had the shortest confidence interval, achieving 1.88 relative efficiency with respect to SL DML and 1.35 relative efficiency with respect to the SL cal. The results from UL methods were questionable as we observed a significant deviation of the UL crude estimation from the SL crude estimation, indicating substantial bias from imperfect data. Coupled with the short confidence intervals, researcher should take caution in the risk of misleading conclusions from the UL methods.

## 7 General Efficiency Lower Bound

While the paper focused on the method for ATE under double missing SSL setting, we established the theoretical efficient lower bound for general parameter and broader missing data pattern in this section. We considered a generic model for data (R,RZ,W) with always observed W and MCAR Z. Specifically, consider

$${𝒮}_{\text{SSL}}=\left\{d{\text{P}}_{f}\left(r,z,w,r\right)={\left[\rho_{\text{N}}f\left(z,w\right)\right]}^{r}{\left[\left(1-\rho_{\text{N}}\right)\int_{z\in 𝒵}f\left(z,w\right)d\nu_{z}\left(z\right)\right]}^{\left(1-r\right)}d\nu_{\text{SSL}}\left(r,z,w\right):\right.\;\left.f\left(z,w\right)d\nu_{\text{cmp}}\left(z,w\right)\in {𝒮}_{\text{cmp}}\right\}$$

for a complete data model class ${𝒮}_{\text{cmp}}$ over 𝒵⊗𝒲 and measures $\nu_{z}$ over $𝒵,\nu_{w}$ over 𝒲 and

$$\nu_{\text{cmp}}=\nu_{z}\times \nu_{w},\nu_{\text{SSL}}\left(r,z,w\right)=\delta_{1}\left(r\right)\times \nu_{\text{cmp}}\left(z,w\right)+\delta_{0}\left(r\right)\times \nu_{w}\left(w\right).$$

Let ℋ be the nuisance tangent space of ${𝒮}_{\text{SSL}}$ at the true model $dP_{f_{*}}$ with $f=f_{*}$. Suppose $\psi_{\text{cmp}}(Z,W)$ is the efficient influence function for parameter θ under ${𝒮}_{\text{cmp}}$. Here we use a different notation ψ for general parameter under missing data components to distinguish from the ϕ used specifically for ATE under double missing SSL setting. Our theory was established under the following basic assumptions.

**Assumption 5** *For absolute constant* M,

- (MCAR)R⫫(Z,W);
- *(Informative labels)* ${\text{inf}}_{\|v{\|}_{2}=1}\;v^{⊤}\text{Var}\left[\psi_{\text{cmp}}(Z,W)-E\left\{\psi_{\text{cmp}}(Z,W)|W\right\}\right]v\geq 1/M$;
- *(Model flexibility)* $E_{*}\left\{\psi_{\text{cmp}}(Z,W)|W\right\}\in ℋ$;
- *(Bounded influence function)* ${\left\|\psi_{\text{cmp}}(Z,W)\right\|}_{2}\leq M$ *almost surely*.

We derived the SSL efficient influence function by the following proposition.

**Proposition 12** *Let* $\psi_{\text{cmp}}(Z,W)$ *be the efficient influence function for parameter θ under complete data model* ${𝒮}_{\text{cmp}}$. *Under Assumptions 5a and 5c, the efficient influence function for* θ *under SSL model* ${𝒮}_{\text{SSL}}$ *is*

(19) $$\psi_{\text{SSL}}\left(R,Z,W\right)=\frac{R}{\rho_{N}}\left[\psi_{\text{cmp}}\left(Z,W\right)-E\left\{\psi_{\text{cmp}}\left(Z,W\right)|W\right\}\right]+E\left\{\psi_{\text{cmp}}\left(Z,W\right)|W\right\}.$$

The influence function $\psi_{\text{SSL}}$ leads to a semi-parametric efficiency lower bound.

**Theorem 13** *Under Assumptions 5a-5d, we have the minimax semi-parametric efficiency for SSL of* θ *under* ${𝒮}_{\text{SSL}}$,

$$\underset{a:\|a{\|}_{2}=1}{\text{inf}}\underset{c\to \infty }{\text{lim}\;\text{inf}}\underset{N\to \infty }{\text{lim}\;\text{inf}}\underset{\hat{\theta }}{\text{inf}}\underset{{\left\|f-f_{*}\right\|}_{\text{TV}}\leq c/\sqrt{\rho_{N}N}}{\text{sup}}\frac{\int N{\left\{a^{⊤}\left(\hat{\theta }-\theta^{*}\right)\right\}}^{2}d\prod_{i=1}^{N}P_{f}\left(z_{i},w_{i},r_{i}\right)}{a^{⊤}\text{Var}\left\{\psi_{\text{SSL}}(R,Z,W)\right\}a}\geq 1.$$

We offered the proof of Theorem 13 in Section C6 of the Supplementary Materials. Upper bound would depend on the context. Like Corollary 7, the bound can be attained if non-parametric estimation of nuisance models admit sufficiently fast rate of consistency, which has been thoroughly studied under classical low-dimensional settings by Stone (1977, 1982). While we focus on $\rho_{N}\to 0$ and n≪N setting, the theory also applies to classical setting with $\rho_{N}\in [1/M,1-1/M]$ and n≍N setting.

## 8 Discussion

Motivated by the increasing interest of generating real-world evidence on treatment effect with big yet noisy EHR data, we proposed a robust and efficient semi-supervised estimator for ATE under the double missing SSL setting. The SMMAL estimator gained efficiency by leveraging the large unlabelled data containing noisy yet predictive surrogates for Y and A with almost no additional requirement than those needed for the supervised analysis using the labeled set alone. We established semi-parametric efficiency bound for the ATE estimator under the low dimensional confounder setting and constructed a doubly robust SMMAL estimator for the high dimensional confounder setting.

Unlike the MCAR setting, the missing data propensity score P(R=1∣W)=ρ(W) must be modeled and estimated. We conjecture that the efficient influence function under MAR may take the form

$$\phi_{\text{MAR}}(\text{RY},\text{RA},W,R)=E\left\{\phi_{\text{cmp}}(Y,A,X)|W\right\}\;+\frac{R}{\rho (W)}\left[\phi_{\text{cmp}}(Y,A,X)-E\left\{\phi_{\text{cmp}}(Y,A,X)|W\right\}\right].$$

The estimation of the decaying ρ(W) has been studied in Zhang et al. (2023). When all nuisance models, (μ,π,Π,m,ρ), are consistently estimated at suitable rates, the efficiency lower bound should be attained under ideal conditions. However, extension of the SMMAL with high-dimensional regressions to MAR setting would require a more sophisticated calibration procedure for all 5 models (μ,π,Π,m,ρ), as the potential bias from mis-specified ρ now may impact the orthogonality of ATE estimator toward all 4 other estimated models. Moreover, caution must be taken when making MAR assumption for treatment and outcome data from linked observational data such as a disease registry. Enrollment in registry led by pioneering clinical experts may systematically impact the treatment pattern and care quality, which would put the MAR assumption in doubt.

The classical semi-parametric efficiency theory relies on the correct modeling and estimation of the nuisance models. When some nuisance models cannot be consistently estimated, there is no universal efficiency guarantee for estimation procedures derived from semi-parametric efficiency theory. To ensure efficiency improvement when both the supervised estimator

$${\hat{\Delta }}_{\text{SL}}=\frac{1}{N}\sum_{k=1}^{K}\sum_{i\in {ℐ}_{k}}\frac{R_{i}}{\rho_{\text{N}}}\left[{\hat{\mu }}^{\left(k\right)}\left(1,X_{i}\right)+\frac{A_{i}}{{\hat{\pi }}^{\left(k\right)}\left(1,X_{i}\right)}\left\{Y_{i}-{\hat{\mu }}^{\left(k\right)}\left(1,X_{i}\right)\right\}\right]\;-\frac{R_{i}}{\rho_{\text{N}}}\left[{\hat{\mu }}^{\left(k\right)}\left(0,X_{i}\right)+\frac{1-A_{i}}{{\hat{\pi }}^{\left(k\right)}\left(0,X_{i}\right)}\left\{Y_{i}-{\hat{\mu }}^{\left(k\right)}\left(0,X_{i}\right)\right\}\right]$$

and the SMMAL estimator ${\hat{\Delta }}_{\text{SMMAL}}$ are consistent and asymptotically normal, we may consider the linear ensemble

$${\hat{\Delta }}_{\text{comb}}={\hat{\Delta }}_{\text{SMMAL}}+b\left({\hat{\Delta }}_{\text{SL}}-{\hat{\Delta }}_{\text{SMMAL}}\right).$$

Suppose the influence functions for ${\hat{\Delta }}_{\text{SMMAL}}$ and ${\hat{\Delta }}_{\text{SL}}$ are $\phi_{\text{SSL}}$ and $R\phi_{\text{cmp}}/\rho_{N}$, respectively. The optimal linear ensemble is given by

$$b_{\text{opt}}=\frac{\text{Var}\left(\phi_{\text{SSL}}\right)-\text{Cov}\left(\phi_{\text{SSL}},R\phi_{\text{cmp}}/\rho_{N}\right)}{\text{Var}\left(\phi_{\text{SSL}}\right)+\text{Var}\left(R\phi_{\text{cmp}}/\rho_{N}\right)-2\text{Cov}\left(\phi_{\text{SSL}},R\phi_{\text{cmp}}/\rho_{N}\right)},$$

which can be estimated by the empirical variances and covariance of estimated influence functions constructed with estimated nuisance models $\left(\hat{\mu },\hat{\pi },\hat{m},\hat{\Pi }\right)$.

Our doubly robust estimation can be generalized to other models if the calibrated estimation for the model is available. For example, we can directly adopt the estimators from Tan (2020) for linear outcome model. The calibrated estimation is, however, limited to M-estimator in high-dimensional regression due to the paucity of works on Z-estimators in high-dimensional setting. It would be interesting to study if the Z-estimator approach (Vermeulen and Vansteelandt, 2015) can be generalized to high-dimensional setting.

## Supplementary Material

1

## Figures and Tables

**Table 1:** List of parameters used in the mixture Beta distribution for the surrogates.

Setting	OK	Reasonable	Good	Great	Perfect
AUC	0.80	0.90	0.95	0.99	0.999
Low-dimensional smooth model					
$\alpha_{A}$	1.39	1.99	2.54	3.86	5.49
$\alpha_{Y}$	1.39	1.96	2.57	3.80	5.70
High-dimensional logistic regression					
$\alpha_{A}$	1.36	1.99	2.54	3.80	5.64
$\alpha_{Y}$	1.33	1.96	2.51	3.89	5.55
High-dimensional regression: mis-specified PS					
$\alpha_{A}$	1.36	1.96	2.54	3.80	5.55
$\alpha_{Y}$	1.39	1.93	2.54	3.74	5.52
High-dimensional regression: mis-specified OR					
$\alpha_{A}$	1.36	1.96	2.54	3.80	5.55
$\alpha_{Y}$	1.39	1.93	2.54	3.74	5.52

**Table 2:** **Accuracy of extracted EHR feature counts** for targeted therapy and 1-year progression (defined as new metastasis site) free survival from EHR valided over 100 patients reviewed by abstractor. False positive rates (FPR) and false negative rates (FNR) were calculated by the dichotomized extractions: Benchmark features – count > 0; **Engineered features** – classification by the quantiles matching prevalence in gold-standard labels. Area under reception operating curve (AUC) were calculated using count/score as predictor (death encoded as a very large value 1000).

Surrogate	FPR	FNR	AUC
Targeted Therapy			
Medication Code	0.44	0.17	0.60
**Mention in Note**	0.35*	0.10*	**0.93**
1-year Progression Free Survival			
Death Registry	0.02	0.43	–
Death & New Site Code	0.34	0.20	0.84
Death & New Site in Note	0.31	0.20	0.85
**Terminal-Progression Score**	0.31*	0.10*	**0.93**

Straightforward rule based extraction (indicated by *) failed to capture treatment and response.

Two surrogates in **bold font** were chosen for SMMAL for their **reasonably good AUC**.

**Table 3:** Baseline characteristics of full study cohort and two arms in the labeled subset. The format is “count (percentage %)” for binary/categorical variables and “mean (standard deviation)” for numerical variables.

	Full data	Labeled set	
		Chemotherapy	Targeted Therapy
Size	4147	79	21
**Demographics**			
Age at Metastasis	62.5 (13.8)	65.2 (11.4)	62.7 (15.9)
Female	1926 (46%)	46 (58%)	11 (52%)
White	3470 (84%)	68 (86%)	20 (95%)
**Cancer characteristics at diagnosis**			
Left Colon Tumor	221 (5%)	5 (6%)	0 (0%)
Right Colon Tumor	890 (21%)	23 (29%)	8 (38%)
Transverse Colon Tumor	420 (10%)	9 (11%)	1 (5%)
Sigmoid Colon Tumor	2092 (50%)	44 (56%)	9 (43%)
Rectum Tumor	2002 (48%)	43 (54%)	5 (24%)
Metastasis Code	2674 (64%)	50 (63%)	16 (76%)
Lymph Node Tumor	364 (9%)	10 (13%)	0 (0%)
Stage I	55 (1%)	0 (0%)	0 (0%)
Stage II	159 (4%)	3 (4%)	0 (0%)
Stage II	992 (24%)	22 (28%)	2 (10%)
Stage IV	2546 (61%)	46 (58%)	17 (81%)
Stage Missing	395 (10%)	8 (10%)	2 (10%)
**Cancer characteristics at metastasis**			
Year since Diagnosis	0.7 (2)	0.5 (1)	0.8 (1.7)
Lung Metastasis Code	646 (16%)	10 (13%)	8 (38%)
Liver Metastasis Code	1694 (41%)	27 (34%)	14 (67%)
Liver Metastasis in Note	1422 (34%)	27 (34%)	12 (57%)
**Treatments between diagnosis and metastasis**			
Chemotherapy Code	1.4 (5.3)	1.3 (3.7)	0 (0)
Radiotherapy Code	10.1 (35.1)	10.5 (30.4)	7.2 (29.1)
Colon Biopsy Code	0.6 (1.7)	0.5 (1.5)	0 (0)
Colon Rescission Code	0.4 (0.8)	0.3 (0.7)	0.2 (0.5)
**Healthcare utilization**			
Before Metastasis	29.7 (59.3)	36.2 (74.5)	13 (21.2)
One Year Before Metastasis	9.8 (15.4)	10.2 (14.7)	4.2 (8.1)
