# Supplementary material for: Efficient and Robust Semi-supervised Estimation of Average Treatment Effect with Partially Annotated Treatment and Response
Source: J Mach Learn Res. Author manuscript; Available in PMC 2025 Dec 3. (PMC12671556)
Supplement: 1 [file NIHMS2084485-supplement-1.pdf]

Supplementary Materials

We present the detailed summaries of simulation results in Appendix A and additional information on the treatment and outcome in the data example in Appendix B. The proofs of Theorems 2-13, Corollary 7 and Proposition 12 are given in Appendix C. The technical details in these proofs are put in Appendix D. Definitions and additional details are stated in Appendix E.

Appendix A. Simulation Tables

The detailed simulation results containing the bias, standard deviation, average standard error, coverage of 95% confidence interval for our semi-supervised $\widehat{\Delta}_{\mathsf{SMMAL}}$, $\widehat{\Delta}_{\mathsf{DR}}$ along with those for the supervised benchmarks were presented in Tables A4-A7. Our semi-supervised $\widehat{\Delta}_{\mathsf{SMMAL}}$, $\widehat{\Delta}_{\mathsf{DR}}$ achieved reasonably honest inference with coverage of 95% confidence interval close to the nominal level and a better efficiency than the supervised benchmark. The detailed simulation results containing the bias, standard deviation, coverage of 95% confidence interval for the unsupervised benchmarks were presented in Table A8.

Table A4: Detailed simulation results on bias, standard deviation (SD), average standard error (ASE), coverage of 95% confidence interval (Cov) for SMMAL and supervised benchmark (SL) under low-dimensional smooth models. The 25 rows correspond to 5×5 points in **top left plot in Figure 3**, indexed by the designed AUC of surrogates for A (column 1, vertical axis in Figure 3) and Y (column 2, horizontal axis in Figure 3). Bias, standard deviation (SD) and average standard error (ASE) were multiplied by 100.

AUC		SL				SMMAL				RE
A	Y	Bias	SD	ASE	Cov	Bias	SD	ASE	Cov	
0.80	0.80	-0.81	4.08	4.07	0.94	-0.79	4.04	3.98	0.94	1.02
0.90	0.80	-0.85	4.14	4.08	0.94	-0.79	3.97	3.95	0.94	1.09
0.95	0.80	-0.75	3.87	4.09	0.96	-0.71	3.72	3.84	0.95	1.08
0.99	0.80	-0.90	4.16	4.11	0.94	-0.92	3.69	3.66	0.94	1.25
0.999	0.80	-0.56	4.21	4.11	0.94	-0.61	3.64	3.59	0.94	1.33
0.80	0.90	-1.01	4.10	4.07	0.95	-1.00	4.01	3.92	0.94	1.04
0.90	0.90	-0.69	4.08	4.09	0.95	-0.66	3.83	3.85	0.95	1.14
0.95	0.90	-0.70	4.11	4.09	0.95	-0.72	3.70	3.63	0.94	1.22
0.99	0.90	-0.66	4.21	4.10	0.94	-0.61	3.34	3.29	0.94	1.58
0.999	0.90	-0.35	4.02	4.10	0.95	-0.43	3.14	3.13	0.94	1.62
0.80	0.95	-1.14	4.09	4.06	0.94	-1.07	4.01	3.87	0.94	1.05
0.90	0.95	-0.71	3.98	4.08	0.95	-0.70	3.81	3.76	0.94	1.09
0.95	0.95	-0.69	4.01	4.09	0.96	-0.59	3.49	3.51	0.96	1.32
0.99	0.95	-0.27	4.12	4.09	0.94	-0.23	3.06	3.01	0.94	1.80
0.999	0.95	-0.22	4.08	4.10	0.95	-0.20	2.69	2.78	0.96	2.29
0.80	0.99	-0.92	4.07	4.07	0.94	-0.89	3.94	3.86	0.94	1.07
0.90	0.99	-0.71	3.99	4.09	0.95	-0.52	3.63	3.71	0.95	1.22
0.95	0.99	-0.10	4.12	4.09	0.94	-0.30	3.50	3.32	0.93	1.38
0.99	0.99	-0.37	4.05	4.10	0.95	-0.07	2.73	2.61	0.94	2.23
0.999	0.99	-0.22	4.12	4.10	0.95	-0.05	2.31	2.21	0.94	3.17
0.80	0.999	-0.95	3.87	4.08	0.95	-0.94	3.79	4.00	0.95	1.04
0.90	0.999	-0.60	4.10	4.08	0.94	-0.69	3.89	3.87	0.94	1.10
0.95	0.999	-0.39	4.02	4.08	0.94	-0.37	3.67	3.59	0.94	1.20
0.99	0.999	0.09	4.05	4.09	0.94	0.02	3.13	3.04	0.94	1.67
0.999	0.999	0.03	4.08	4.11	0.95	-0.12	2.64	2.64	0.95	2.38

Table A5: Detailed simulation results on bias, standard deviation (SD), average standard error (ASE), coverage of 95% confidence interval (Cov) for SMMAL and supervised benchmark (SL) under high-dimensional models with logistic regression PS and OR. The 25 rows correspond to 5×5 points in **top right plot in Figure 3**, indexed by the designed AUC of surrogates for A (column 1, vertical axis in Figure 3) and Y (column 2, horizontal axis in Figure 3). Bias, standard deviation (SD) and average standard error (ASE) were multiplied by 100.

AUC		SL				SMMAL				RE
A	Y	Bias	SD	ASE	Cov	Bias	SD	ASE	Cov	
0.80	0.80	-0.28	4.71	4.49	0.94	-0.22	4.62	4.47	0.95	1.04
0.90	0.80	-0.24	4.70	4.48	0.95	-0.19	4.63	4.36	0.95	1.03
0.95	0.80	-0.24	4.66	4.49	0.96	-0.20	4.43	4.29	0.95	1.11
0.99	0.80	-0.22	4.65	4.49	0.94	-0.17	4.33	4.18	0.95	1.15
0.999	0.80	-0.32	4.70	4.48	0.95	-0.33	4.33	4.11	0.94	1.18
0.80	0.90	-0.26	4.68	4.49	0.94	-0.16	4.56	4.34	0.94	1.06
0.90	0.90	-0.26	4.64	4.49	0.94	-0.22	4.13	4.09	0.94	1.27
0.95	0.90	-0.33	4.72	4.49	0.94	-0.18	4.13	3.92	0.94	1.31
0.99	0.90	-0.31	4.63	4.49	0.95	0.03	3.79	3.65	0.94	1.50
0.999	0.90	-0.24	4.68	4.48	0.95	-0.07	3.63	3.50	0.94	1.67
0.80	0.95	-0.32	4.69	4.49	0.95	-0.26	4.53	4.27	0.95	1.07
0.90	0.95	-0.31	4.62	4.49	0.95	-0.36	3.94	3.91	0.94	1.37
0.95	0.95	-0.27	4.71	4.49	0.95	-0.27	3.89	3.67	0.94	1.46
0.99	0.95	-0.21	4.71	4.48	0.95	-0.11	3.44	3.28	0.93	1.87
0.999	0.95	-0.33	4.71	4.48	0.93	-0.19	3.21	3.05	0.93	2.15
0.80	0.99	-0.31	4.67	4.49	0.95	-0.25	4.29	4.15	0.95	1.19
0.90	0.99	-0.31	4.68	4.49	0.94	-0.09	3.76	3.64	0.94	1.55
0.95	0.99	-0.28	4.71	4.49	0.94	-0.11	3.46	3.27	0.94	1.86
0.99	0.99	-0.30	4.73	4.49	0.94	-0.09	2.79	2.67	0.94	2.89
0.999	0.99	-0.28	4.70	4.48	0.95	-0.12	2.32	2.27	0.94	4.11
0.80	0.999	-0.30	4.65	4.48	0.96	-0.22	4.26	4.09	0.95	1.19
0.90	0.999	-0.26	4.70	4.49	0.95	-0.04	3.55	3.50	0.96	1.76
0.95	0.999	-0.24	4.68	4.48	0.95	-0.09	3.13	3.06	0.94	2.23
0.99	0.999	-0.27	4.69	4.49	0.95	-0.08	2.39	2.33	0.95	3.86
0.999	0.999	-0.32	4.69	4.49	0.95	-0.05	1.85	1.78	0.94	6.49

Table A6: Detailed simulation results on bias, standard deviation (SD), average standard error (ASE), coverage of 95% confidence interval (Cov) for SMMAL and supervised benchmark (SL) under high-dimensional models with miss-specified PS and correct OR models. The 25 rows correspond to 5 × 5 points in **bottom right plot in Figure 3**, indexed by the designed AUC of surrogates for A (column 1, vertical axis in Figure 3) and Y (column 2, horizontal axis in Figure 3). Bias, standard deviation (SD) and average standard error (ASE) were multiplied by 100.

AUC		SL				SMMAL				RE
A	Y	Bias	SD	ASE	Cov	Bias	SD	ASE	Cov	
0.80	0.80	-0.28	4.67	4.46	0.94	-0.21	4.61	4.43	0.94	1.03
0.90	0.80	-0.31	4.72	4.45	0.94	-0.32	4.55	4.31	0.94	1.08
0.95	0.80	-0.26	4.74	4.45	0.94	-0.17	4.46	4.22	0.94	1.13
0.99	0.80	-0.29	4.67	4.45	0.95	-0.19	4.28	4.09	0.94	1.20
0.999	0.80	-0.29	4.72	4.45	0.95	-0.29	4.21	4.03	0.96	1.25
0.80	0.90	-0.28	4.70	4.45	0.94	-0.26	4.64	4.32	0.94	1.02
0.90	0.90	-0.29	4.71	4.45	0.94	-0.26	4.27	4.08	0.95	1.22
0.95	0.90	-0.30	4.73	4.46	0.95	-0.25	4.18	3.90	0.93	1.28
0.99	0.90	-0.31	4.78	4.46	0.94	0.04	3.95	3.65	0.93	1.47
0.999	0.90	-0.27	4.76	4.46	0.95	-0.04	3.83	3.51	0.94	1.55
0.80	0.95	-0.29	4.70	4.45	0.95	-0.25	4.54	4.23	0.93	1.08
0.90	0.95	-0.32	4.72	4.46	0.94	-0.13	4.03	3.89	0.95	1.37
0.95	0.95	-0.27	4.69	4.45	0.95	-0.10	3.85	3.63	0.93	1.49
0.99	0.95	-0.26	4.72	4.46	0.94	-0.07	3.43	3.24	0.94	1.90
0.999	0.95	-0.31	4.65	4.45	0.94	-0.01	3.16	3.01	0.94	2.18
0.80	0.99	-0.28	4.70	4.45	0.94	-0.22	4.39	4.12	0.94	1.15
0.90	0.99	-0.31	4.72	4.45	0.94	-0.15	3.61	3.65	0.96	1.71
0.95	0.99	-0.23	4.71	4.45	0.94	-0.05	3.45	3.27	0.92	1.87
0.99	0.99	-0.37	4.72	4.46	0.94	-0.12	2.92	2.71	0.95	2.62
0.999	0.99	-0.25	4.75	4.45	0.94	-0.04	2.40	2.32	0.94	3.91
0.80	0.999	-0.32	4.72	4.46	0.94	-0.25	4.31	4.05	0.94	1.20
0.90	0.999	-0.26	4.70	4.45	0.94	-0.09	3.51	3.50	0.96	1.79
0.95	0.999	-0.28	4.69	4.45	0.94	-0.08	3.07	3.04	0.94	2.33
0.99	0.999	-0.32	4.76	4.45	0.94	-0.07	2.38	2.32	0.95	4.03
0.999	0.999	-0.30	4.68	4.46	0.94	0.11	1.81	1.79	0.95	6.68

Table A7: Detailed simulation results on bias, standard deviation (SD), average standard error (ASE), coverage of 95% confidence interval (Cov) for SMMAL and supervised benchmark (SL) under high-dimensional models with correct PS and miss-specified OR models. The 25 rows correspond to 5 × 5 points in **bottom left plot in Figure 3**, indexed by the designed AUC of surrogates for A (column 1, vertical axis in Figure 3) and Y (column 2, horizontal axis in Figure 3). Bias, standard deviation (SD) and average standard error (ASE) were multiplied by 100.

AUC		SL				SMMAL				RE
A	Y	Bias	SD	ASE	Cov	Bias	SD	ASE	Cov	
0.80	0.80	-0.21	4.70	4.49	0.95	-0.19	4.70	4.46	0.94	1.00
0.90	0.80	-0.20	4.75	4.49	0.95	-0.22	4.60	4.35	0.94	1.07
0.95	0.80	-0.16	4.64	4.49	0.96	-0.18	4.35	4.27	0.94	1.13
0.99	0.80	-0.22	4.64	4.49	0.95	-0.14	4.36	4.15	0.94	1.13
0.999	0.80	-0.17	4.74	4.49	0.95	-0.19	4.30	4.09	0.95	1.21
0.80	0.90	-0.20	4.69	4.48	0.95	-0.23	4.50	4.33	0.95	1.08
0.90	0.90	-0.23	4.70	4.48	0.95	-0.24	4.21	4.10	0.94	1.24
0.95	0.90	-0.25	4.75	4.48	0.95	-0.16	3.99	3.92	0.95	1.42
0.99	0.90	-0.22	4.67	4.49	0.95	0.01	3.83	3.66	0.95	1.49
0.999	0.90	-0.26	4.68	4.48	0.95	-0.15	3.68	3.51	0.94	1.62
0.80	0.95	-0.18	4.67	4.48	0.96	-0.21	4.41	4.25	0.93	1.12
0.90	0.95	-0.19	4.69	4.48	0.95	-0.19	3.99	3.93	0.95	1.38
0.95	0.95	-0.21	4.67	4.48	0.96	-0.03	3.65	3.68	0.95	1.64
0.99	0.95	-0.21	4.67	4.48	0.95	-0.32	3.38	3.29	0.94	1.90
0.999	0.95	-0.17	4.65	4.49	0.96	0.09	3.20	3.07	0.94	2.12
0.80	0.99	-0.18	4.64	4.49	0.96	-0.21	4.20	4.13	0.95	1.22
0.90	0.99	-0.23	4.65	4.49	0.95	-0.25	3.72	3.68	0.95	1.56
0.95	0.99	-0.16	4.71	4.48	0.95	-0.05	3.27	3.30	0.95	2.08
0.99	0.99	-0.22	4.61	4.49	0.95	-0.08	2.81	2.70	0.94	2.70
0.999	0.99	-0.25	4.69	4.48	0.96	-0.01	2.51	2.32	0.92	3.51
0.80	0.999	-0.20	4.67	4.48	0.95	-0.08	4.09	4.06	0.96	1.31
0.90	0.999	-0.17	4.71	4.48	0.95	-0.09	3.60	3.53	0.95	1.71
0.95	0.999	-0.21	4.60	4.48	0.95	-0.08	3.02	3.09	0.95	2.33
0.99	0.999	-0.24	4.68	4.48	0.95	-0.05	2.39	2.35	0.95	3.85
0.999	0.999	-0.17	4.67	4.48	0.95	0.11	1.94	1.84	0.94	5.81

Table A8: Detailed simulation results on bias, standard deviation (SD) and coverage of 95 % confidence interval (Cov) for unsupervised analyses under **settings in Figure 4** low-dimensional smooth model (Low-d, top left plot in Figure 4), high-dimensional logistic model (High-d, top right plot), high-dimensional model with mis-specified propensity scores (High-d misPS, bottom right plot in Figure 4), high-dimensional model with mis-specified outcome regression (High-d misOR, bottom left plot in Figure 4). The 25 rows correspond to 5×5 points in each plot in Figure 4, indexed by the designed AUC of surrogates for A (column 1, vertical axis in Figure 4) and Y (column 2, horizontal axis in Figure 4). Bias and standard deviation (SD) were multiplied by 100.

AUC		Low-d			High-d			High-d misPS			High-d misOR		
A	Y	Bias	SD	Cov	Bias	SD	Cov	Bias	SD	Cov	Bias	SD	Cov
0.80	0.80	-9.08	0.92	0.00	-2.55	1.04	0.27	-2.57	1.04	0.29	-2.87	0.97	0.16
0.90	0.80	-8.37	0.92	0.00	-2.14	0.98	0.44	-2.12	0.98	0.45	-2.47	0.95	0.31
0.95	0.80	-7.85	0.99	0.00	-1.77	1.04	0.58	-1.75	1.04	0.59	-2.10	1.07	0.48
0.99	0.80	-7.15	0.99	0.00	-1.36	1.06	0.73	-1.38	1.09	0.72	-1.59	1.03	0.68
0.999	0.80	-6.94	1.05	0.00	-1.25	1.06	0.77	-1.13	1.09	0.80	-1.47	1.09	0.69
0.80	0.90	-8.47	0.94	0.00	-2.04	1.00	0.48	-2.04	1.05	0.46	-2.46	0.94	0.29
0.90	0.90	-7.18	0.98	0.00	-1.66	1.04	0.61	-1.73	0.92	0.61	-1.92	1.01	0.54
0.95	0.90	-6.31	0.98	0.00	-1.35	0.96	0.74	-1.42	1.11	0.68	-1.61	1.07	0.61
0.99	0.90	-5.27	0.99	0.00	-1.01	1.09	0.83	-0.96	1.11	0.80	-1.19	1.08	0.78
0.999	0.90	-4.75	1.01	0.00	-0.80	1.12	0.87	-0.82	1.10	0.86	-1.03	1.15	0.81
0.80	0.95	-8.09	0.95	0.00	-1.77	1.01	0.59	-1.77	1.03	0.58	-2.15	0.99	0.43
0.90	0.95	-6.42	0.99	0.00	-1.37	1.02	0.73	-1.46	1.00	0.70	-1.74	1.04	0.60
0.95	0.95	-5.29	1.00	0.00	-1.15	1.07	0.79	-1.21	1.09	0.77	-1.34	1.02	0.74
0.99	0.95	-4.11	1.02	0.01	-0.75	1.16	0.86	-0.75	1.12	0.87	-0.96	1.13	0.81
0.999	0.95	-3.50	0.96	0.03	-0.60	1.14	0.89	-0.59	1.17	0.88	-0.74	1.11	0.87
0.80	0.99	-7.53	0.93	0.00	-1.39	1.05	0.72	-1.49	1.00	0.69	-1.74	0.96	0.58
0.90	0.99	-5.40	0.99	0.00	-1.05	1.02	0.81	-1.16	0.97	0.79	-1.38	0.98	0.74
0.95	0.99	-3.95	0.97	0.01	-0.82	1.09	0.86	-0.86	1.06	0.86	-0.97	1.04	0.86
0.99	0.99	-2.46	1.02	0.25	-0.47	1.14	0.91	-0.51	1.11	0.90	-0.58	1.16	0.87
0.999	0.99	-1.65	1.04	0.57	-0.31	1.11	0.93	-0.33	1.14	0.92	-0.44	1.14	0.90
0.80	0.999	-7.29	0.96	0.00	-1.23	1.00	0.76	-1.28	1.01	0.75	-1.60	0.98	0.63
0.90	0.999	-5.01	1.01	0.00	-0.93	1.02	0.84	-1.01	0.95	0.83	-1.20	0.96	0.78
0.95	0.999	-3.53	1.06	0.04	-0.71	1.06	0.89	-0.73	1.06	0.86	-0.84	1.06	0.87
0.99	0.999	-1.82	1.03	0.51	-0.38	1.15	0.91	-0.40	1.14	0.91	-0.48	1.13	0.90
0.999	0.999	-1.03	1.00	0.77	-0.23	1.15	0.92	-0.20	1.15	0.92	-0.29	1.17	0.92

Table A9: Treatment, outcomes and their surrogates in full study cohort and two arms in the labeled subset. The format is "count (percentage %)" for binary variables and "mean (standard deviation)" for numerical variables.

	Full data	Labeled set	
Size	4147	100	
**Treatment Arms**		Chemotherapy	Targeted Therapy
Gold-standard labels	–	79	21
EHR proxies for treatment between metastasis and treatment			
Targeted Medication Code Count	0.2 (0.7)	0.1 (0.4)	0.6 (1.2)
Targeted Therapy Mention Count in Note	4.6 (13.6)	1.4 (3.9)	14.4 (24.6)
**Endpoints up to 1 year after treatment:**			
Gold-standard labels			
Terminal Condition	–	18 (23%)	11 (52%)
New Metastasis	–	7 (9%)	2 (10%)
EHR proxies for outcome during 1 year follow-up			
Occurrence of death record	781 (19%)	12 (15%)	9 (43%)
Diagnosis Code Count in Last Month	33.5 (50.8)	34.1 (49)	53.4 (52.6)
Procedure Code Count in Last Month	1.9 (4.8)	2 (5)	2.8 (4.9)
New Metastasis Code Counts	3.2 (9.3)	4.9 (10.7)	1.9 (4.9)

Appendix B. Treatment and Outcomes in Data Example

We report the treatment and outcome labels as well as their EHR proxies in Table A9. The targeted therapy arm was marginally associated with poorer outcomes in terms of terminal conditions (52 % vs 23 %). Tables A9 shows that occurrence of EHR proxies cannot accurately indicate prescription information. We used the log counts of targeted medication mention in note from metastasis to treatment as the surrogate for treatment indicator due to its large contrast between the two labeled arms. Progress-free survival is poorly structured in EHR with no clear indicator. We construct a terminal-progression score with reasonably good prediction power (see Table 2) using death records, activity (diagnosis and procedure codes) in last EHR month and metastasis code for a new site during 1 year follow-up.

Appendix C. Proofs of Main Text Theorems

To analyze the cross-fitting, we adopt the following notations for the conditional expectations given different part of the data.

Definition A14 *Recall that we denote the full data, fold-k data, out-of-fold-k data and out-of-folds-k_1-k_2 data as $\mathscr{D}$, $\mathscr{D}_k$, $\mathscr{D}_k^c$ and $\mathscr{D}_{k_1,k_2}^c$, respectively. The conditional expectation for samples with index in set $\mathcal{I}$ conditionally on subset of the data $\mathscr{D}'$ is denoted as*

$$\mathbb{E}_{i\in\mathcal{I}}\{f(\mathbf{D}_i) \mid \mathscr{D}\},\ \mathcal{I} \subseteq \{1,\ldots,n+N\}, \mathscr{D}' \subseteq \mathscr{D}.$$

C1 Proof of Theorem 2

We first analyze the bias terms in $\widehat{\Delta}_{\mathsf{SMMAL}}$ and $\widehat{\mathcal{V}}_{\mathsf{SMMAL}}$ from the cross-fitted estimators through a lemma. We group the limiting nuisance models as $\bar{\boldsymbol{\eta}} = (\pi_*, \mu_*, \bar{\Pi}, \bar{m}_*)$. In the proof, we repeat the same analyze on two components for treatment arms in ϕ_{SSL},

$$\begin{aligned}\phi_{\mathsf{SSL,a}}(\mathbf{D};\boldsymbol{\eta}) =& \mu(a,\mathbf{X}) + \frac{\mathrm{I}(A=a)}{\pi(a,\mathbf{X})}\{Y-\mu(a,\mathbf{X})\} && \text{(A.20)}\\ &+ \frac{1}{\pi(a,\mathbf{X})}\left\{\frac{R}{\rho_{\mathsf{N}}}-1\right\}\{\mathrm{I}(A=a)Y - \Pi(a,\mathbf{W})m(a,\mathbf{W})\}\\ &- \frac{\mu(a,\mathbf{X})}{\pi(a,\mathbf{X})}\left\{\frac{R}{\rho_{\mathsf{N}}}-1\right\}\{\mathrm{I}(A=a) - \Pi(a,\mathbf{W})\}. && \text{(A.21)}\end{aligned}$$

Notice the connection of $\phi_{\mathsf{SSL,a}}$ to the efficient influence function when $\boldsymbol{\eta}$ equals the true models $\boldsymbol{\eta}_*$ $\phi_{\mathsf{SSL}}(RY, RA, \mathbf{W}, R) = \phi_{\mathsf{SSL,1}}(\mathbf{D};\boldsymbol{\eta}*) - \phi_{\mathsf{SSL,0}}(\mathbf{D};\boldsymbol{\eta}*) - \Delta_*$. We may identify the average treatment by $\Delta_* = \mathbb{E}\{\mu(1,\mathbf{X}) - \mu(0,\mathbf{X})\} = \mathbb{E}\{\phi_{\mathsf{SSL,1}}(\mathbf{D};\bar{\boldsymbol{\eta}}) - \phi_{\mathsf{SSL,0}}(\mathbf{D};\bar{\boldsymbol{\eta}})\}$.

Lemma A15 *Let $\boldsymbol{\eta}_n = (\pi_n, \mu_n, \Pi_n, m_n)$ be a (deterministic) sequence of nuisance models satisfying almost surely*

$$\sup_{a=0,1} \max\{|1/\pi_n(a,\mathbf{X}_i)|, |\mu_n(a,\mathbf{X}_i)|, |\Pi_n(a,\mathbf{W}_i)|, |m_n(a,\mathbf{W}_i)|\} \le M. \quad \text{(A.22)}$$

Under Assumptions 2a and 2b, we have

1. *For bias:*
$$|\mathbb{E}\{\phi_{\mathsf{SSL,a}}(\mathbf{D};\boldsymbol{\eta}_n) - \phi_{\mathsf{SSL,a}}(\mathbf{D};\bar{\boldsymbol{\eta}})\}| \lesssim \|\pi_n - \pi_*\|_2\|\mu_n - \mu_*\|_2,$$

2. *For variance:*
$$\begin{aligned}&\rho_{\mathsf{N}}\,\mathrm{Var}\{\phi_{\mathsf{SSL,a}}(\mathbf{D};\boldsymbol{\eta}_n) - \phi_{\mathsf{SSL,a}}(\mathbf{D};\bar{\boldsymbol{\eta}})\}\\ \lesssim& \|\pi_n - \pi_*\|_2^2 + \|\mu_n - \mu_*\|_2^2 + \|m_n - \bar{m}\|_2^2 + \|\Pi_n - \bar{\Pi}\|_2^2,\end{aligned}$$

3. *For variance estimation:*
$$\begin{aligned}&\mathbb{E}\left\{\rho_{\mathsf{N}}\phi_{\mathsf{SSL,a}}(\mathbf{D};\boldsymbol{\eta}_n)^2\right\} - \mathbb{E}\left\{\rho_{\mathsf{N}}\phi_{\mathsf{SSL,a}}(\mathbf{D};\bar{\boldsymbol{\eta}})^2\right\}\\ \lesssim& \|\pi_n - \pi_*\|_2 + \|\mu_n - \mu_*\|_2 + \|m_n - \bar{m}\|_2 + \|\Pi_n - \bar{\Pi}\|_2\\ &+ \|\pi_n - \pi_*\|_2^2 + \|\mu_n - \mu_*\|_2^2 + \|m_n - \bar{m}\|_2^2 + \|\Pi_n - \bar{\Pi}\|_2^2.\end{aligned}$$

Proof [Proof of Lemma A15] We prove the lemma through a calculation of the expectations and variances of the quantities of interest. To simplify our notation, we denote

$$\triangle\mu = \mu_n(a,\mathbf{X}) - \mu_*(a,\mathbf{X}),\ \triangle\pi = \pi_n(a,\mathbf{X}) - \pi_*(a,\mathbf{X}), \tag{A.23}$$
$$\triangle m = m_n(a,\mathbf{W}) - \bar{m}(a,\mathbf{W}),\ \triangle\Pi = \Pi_n(a,\mathbf{W}) - \bar{\Pi}(a,\mathbf{W}). \tag{A.24}$$

We substitute Y and A in analysis by the model definition

$$\mathbb{E}\{\mathrm{I}(A=a) \mid \mathbf{X}\} = \pi_*(a,\mathbf{X}),\ \mathbb{E}\{\mathrm{I}(A=a)Y \mid \mathbf{X}\} = \pi_*(a,\mathbf{X})\mu_*(a,\mathbf{X}),$$
$$\mathbb{E}\{\mathrm{I}(A=a) \mid \mathbf{W}\} = \bar{\Pi}(a,\mathbf{W}),\ \mathbb{E}\{\mathrm{I}(A=a)Y \mid \mathbf{W}\} = \bar{\Pi}(a,\mathbf{W})\bar{m}(a,\mathbf{W}).$$

1. **For bias:**

   We decompose the expectation into

$$\begin{aligned}
&\mathbb{E}\left\{\phi_{\mathsf{SSL,a}}(\mathbf{D};\boldsymbol{\eta}_n) - \phi_{\mathsf{SSL,a}}(\mathbf{D};\bar{\boldsymbol{\eta}})\right\}\\
=&\underbrace{\mathbb{E}\left\{\phi_{\mathsf{SSL,a}}(\mathbf{D};(\pi_n,\mu_n,\Pi_n,m_n)) - \phi_{\mathsf{SSL,a}}(\mathbf{D};(\pi_n,\mu_*,\Pi_n,m_n))\right\}}_{T_1}\\
&+\underbrace{\mathbb{E}\left\{\phi_{\mathsf{SSL,a}}(\mathbf{D};(\pi_n,\mu_*,\Pi_n,m_n)) - \phi_{\mathsf{SSL,a}}(\mathbf{D};(\pi_*,\mu_*,\Pi_n,m_n))\right\}}_{T_2}\\
&+\underbrace{\mathbb{E}\left\{\phi_{\mathsf{SSL,a}}(\mathbf{D};(\pi_*,\mu_*,\Pi_n,m_n)) - \phi_{\mathsf{SSL,a}}(\mathbf{D};(\pi_*,\mu_*,\bar{\Pi},\bar{m}))\right\}}_{T_3},
\end{aligned}$$

   which we shall analyze separately.

   First, note that T_1 can be written as

$$\begin{aligned}
T_1 =&\mathbb{E}\left(\triangle\mu\left[1 - \frac{I(A=a)}{\pi_n(a,\mathbf{X})} - \frac{1}{\pi_n(a,\mathbf{X})}\left(\frac{R}{\rho_{\mathsf{N}}} - 1\right)\{I(A=a) - \Pi_n(a,\mathbf{W})\}\right]\right)\\
=&\mathbb{E}\left\{-\triangle\mu\triangle\pi/\pi_n(a,\mathbf{X})\right\}.
\end{aligned}$$

   As a result, we have a bound for T_1 by (A.22) and the Cauchy-Schwartz inequality to obtain $|T_1| \leq M\|\triangle\mu\|_2\|\triangle\pi\|_2$. Second, we calculate T_2,

$$\begin{aligned}
T_2 =&\mathbb{E}\left[\frac{\triangle\pi}{\pi_n(a,\mathbf{X})\pi_*(a,\mathbf{X})}\left(\frac{R}{\rho_{\mathsf{N}}} - 1\right)\{I(A=a)Y - \Pi_n(a,\mathbf{W})m_n(a,\mathbf{W})\}\right]\\
&-\mathbb{E}\left[\frac{\triangle\pi}{\pi_n(a,\mathbf{X})\pi_*(a,\mathbf{X})}\mu_*(a,\mathbf{X})\left(\frac{R}{\rho_{\mathsf{N}}} - 1\right)\{I(A=a) - \Pi_n(a,\mathbf{W})\}\right]\\
=&0.
\end{aligned}$$

   Third, we calculate T_3,

$$\begin{aligned}
T_3 =&\mathbb{E}\left[\frac{1}{\pi_*(a,\mathbf{X})}\left(\frac{R}{\rho_{\mathsf{N}}} - 1\right)\{I(A=a)Y - \Pi_n(a,\mathbf{W})m_n(a,\mathbf{W})\}\right]\\
&-\mathbb{E}\left[\frac{1}{\pi_*(a,\mathbf{X})}\mu_*(a,\mathbf{X})\left(\frac{R}{\rho_{\mathsf{N}}} - 1\right)\{I(A=a) - \Pi_n(a,\mathbf{W})\}\right]
\end{aligned}$$

$$
\begin{aligned}
&-\mathbb{E}\left[\frac{1}{\pi_*(a,\mathbf{X})}\left(\frac{R}{\rho_{\mathsf{N}}}-1\right)\{I(A=a)Y-\bar{\Pi}(a,\mathbf{W})\bar{m}(a,\mathbf{W})\}\right]\\
&+\mathbb{E}\left[\frac{1}{\pi_*(a,\mathbf{X})}\mu_*(a,\mathbf{X})\left(\frac{R}{\rho_{\mathsf{N}}}-1\right)\{I(A=a)-\bar{\Pi}(a,\mathbf{W})\}\right]\\
=&0.
\end{aligned}
$$

Putting the bounds for T_1-T_3 together, we have therefore have that

$$
|\mathbb{E}\left\{\phi_{\mathsf{SSL,a}}(\mathbf{D};\boldsymbol{\eta}_n)-\phi_{\mathsf{SSL,a}}(\mathbf{D};\bar{\boldsymbol{\eta}})\right\}|\lesssim\|\pi_n-\pi_*\|_2\|\mu_n-\mu_*\|_2.
$$

2. **For variance:**

Here, we first establish the order for the second moments of terms with $(R/\rho_{\mathsf{N}}-1)$ in them as follows

$$
\begin{aligned}
&\mathbb{E}[\{h_1(Y,A,\mathbf{W})+(R/\rho_{\mathsf{N}}-1)h_2(Y,A,\mathbf{W})\}^2]\\
=&\mathbb{E}\{h_1(Y,A,\mathbf{W})^2\}+\mathbb{E}\{(R/\rho_{\mathsf{N}}-1)^2\}\mathbb{E}\{h_2(Y,A,\mathbf{W})^2\}\\
=&\|h_1\|_2^2+(1/\rho_{\mathsf{N}}-1)\|h_2\|_2^2\\
\leq&\|h_1\|_2^2+\|h_2^2\|_2/\rho_{\mathsf{N}}.
\end{aligned}
\tag{A.25}
$$

The bound for the variance is derived from the bound for the second moment

$$
\mathrm{Var}\left\{\phi_{\mathsf{SSL,a}}(\mathbf{D};\boldsymbol{\eta}_n)-\phi_{\mathsf{SSL,a}}(\mathbf{D};\bar{\boldsymbol{\eta}})\right\}\leq\mathbb{E}\left[\left\{\phi_{\mathsf{SSL,a}}(\mathbf{D};\boldsymbol{\eta}_n)-\phi_{\mathsf{SSL,a}}(\mathbf{D};\bar{\boldsymbol{\eta}})\right\}^2\right].
$$

By the inequality $(a+b+c)^2\leq 4(a^2+b^2+c^2)$ for any $a,b,c\in\mathbb{R}$, we can control the bound in the decomposition:

$$
\begin{aligned}
&\mathbb{E}\left[\left\{\phi_{\mathsf{SSL,a}}(\mathbf{D};\boldsymbol{\eta}_n)-\phi_{\mathsf{SSL,a}}(\mathbf{D};\bar{\boldsymbol{\eta}})\right\}^2\right]\\
=&4\underbrace{\mathbb{E}\left[\left\{\phi_{\mathsf{SSL,a}}(\mathbf{D};(\pi_n,\mu_n,\Pi_n,m_n))-\phi_{\mathsf{SSL,a}}(\mathbf{D};(\pi_n,\mu_*,\Pi_n,m_n))\right\}^2\right]}_{T_1'}\\
&+4\underbrace{\mathbb{E}\left[\left\{\phi_{\mathsf{SSL,a}}(\mathbf{D};(\pi_n,\mu_*,\Pi_n,m_n))-\phi_{\mathsf{SSL,a}}(\mathbf{D};(\pi_*,\mu_*,\Pi_n,m_n))\right\}^2\right]}_{T_2'}\\
&+4\underbrace{\mathbb{E}\left[\left\{\phi_{\mathsf{SSL,a}}(\mathbf{D};(\pi_*,\mu_*,\Pi_n,m_n))-\phi_{\mathsf{SSL,a}}(\mathbf{D};(\pi_*,\mu_*,\bar{\Pi},\bar{m}))\right\}^2\right]}_{T_3'}.
\end{aligned}
$$

Under Assumptions 2a, 2b and (A.22), since we have everything except for R/ρ_{N} bounded in T_1'-T_3', we can derive upper bounds on their rates as follows:

$$
\begin{aligned}
T_1'=&\mathbb{E}\left(\triangle\mu^2\left[1-\frac{I(A=a)}{\pi_n(a,\mathbf{X}_i)}-\frac{1}{\pi_n(a,\mathbf{X}_i)}\left(\frac{R}{\rho_{\mathsf{N}}}-1\right)\{I(A=a)-\Pi_n(a,\mathbf{W})\}\right]^2\right)\\
\lesssim&\|\mu_n-\mu_*\|_2^2/\rho_{\mathsf{N}},
\end{aligned}
$$

$$
\begin{aligned}
T_2' =&\mathbb{E}\left(\left(\frac{\triangle\pi}{\pi_n(a,\mathbf{X})\pi_*(a,\mathbf{X})}\right)^2\left[\left(\frac{R}{\rho_{\mathsf{N}}}-1\right)\{I(A=a)Y-\Pi_n(a,\mathbf{W})m_n(a,\mathbf{W})\}\right.\right.\\
&\left.\left.-\mu_*(a,\mathbf{X})\left(\frac{R}{\rho_{\mathsf{N}}}-1\right)\{I(A=a)-\Pi_n(a,\mathbf{W})\}\right]^2\right)\\
\lesssim&\|\pi_n-\pi_*\|_2^2/\rho_{\mathsf{N}},\\
T_3' \leq& 2\mathbb{E}\left[\triangle\Pi^2\left\{\frac{\mu_*(a,\mathbf{X})-\bar{m}(a,\mathbf{W})}{\pi_*(a,\mathbf{X})}\left(\frac{R}{\rho_{\mathsf{N}}}-1\right)\right\}^2\right]+2\mathbb{E}\left(\triangle m^2\left[\frac{\Pi_n(a,\mathbf{W})}{\pi_*(a,\mathbf{X})}\left(\frac{R}{\rho_{\mathsf{N}}}-1\right)\right]^2\right)\\
\lesssim&\|\Pi_n-\bar{\Pi}\|_2^2/\rho_{\mathsf{N}}+\|m_n-\bar{m}\|_2^2/\rho_{\mathsf{N}}.
\end{aligned}
$$

Putting the rates for T_1'-T_3' together, we therefore obtain

$$
\begin{aligned}
&\rho_{\mathsf{N}}\operatorname{Var}\{\phi_{\mathsf{SSL,a}}(\mathbf{D};\boldsymbol{\eta}_n)-\phi_{\mathsf{SSL,a}}(\mathbf{D};\bar{\boldsymbol{\eta}})\}\\
\leq&\mathbb{E}\left[\{\phi_{\mathsf{SSL,a}}(\mathbf{D};\boldsymbol{\eta}_n)-\phi_{\mathsf{SSL,a}}(\mathbf{D};\bar{\boldsymbol{\eta}})\}^2\right]\\
\lesssim&\|\pi_n-\pi_*\|_2^2+\|\mu_n-\mu_*\|_2^2+\|m_n-\bar{m}\|_2^2+\|\Pi_n-\bar{\Pi}\|_2^2.
\end{aligned}
$$

3. **For variance estimator:**

We establish the last result by connecting it to the second bound above,

$$
\begin{aligned}
&\mathbb{E}\left\{\rho_{\mathsf{N}}\phi_{\mathsf{SSL,a}}(\mathbf{D};\boldsymbol{\eta}_n)^2\right\}-\mathbb{E}\left\{\rho_{\mathsf{N}}\phi_{\mathsf{SSL,a}}(\mathbf{D};\bar{\boldsymbol{\eta}})^2\right\}\\
\leq&\rho_{\mathsf{N}}\mathbb{E}\left[\{\phi_{\mathsf{SSL,a}}(\mathbf{D};\boldsymbol{\eta}_n)-\phi_{\mathsf{SSL,a}}(\mathbf{D};\bar{\boldsymbol{\eta}})\}^2\right]\\
&+2\rho_{\mathsf{N}}\mathbb{E}\left[\phi_{\mathsf{SSL,a}}(\mathbf{D};\bar{\boldsymbol{\eta}})\{\phi_{\mathsf{SSL,a}}(\mathbf{D};\boldsymbol{\eta}_n)-\phi_{\mathsf{SSL,a}}(\mathbf{D};\bar{\boldsymbol{\eta}})\}\right]\\
\leq&\rho_{\mathsf{N}}\mathbb{E}\left[\{\phi_{\mathsf{SSL,a}}(\mathbf{D};\boldsymbol{\eta}_n)-\phi_{\mathsf{SSL,a}}(\mathbf{D};\bar{\boldsymbol{\eta}})\}^2\right]\\
&+\rho_{\mathsf{N}}\sqrt{\mathbb{E}\{\phi_{\mathsf{SSL,a}}(\mathbf{D};\bar{\boldsymbol{\eta}})^2\}\mathbb{E}\left[\{\phi_{\mathsf{SSL,a}}(\mathbf{D};\boldsymbol{\eta}_n)-\phi_{\mathsf{SSL,a}}(\mathbf{D};\bar{\boldsymbol{\eta}})\}^2\right]}\\
\lesssim&\|\pi_n-\pi_*\|_2+\|\mu_n-\mu_*\|_2+\|m_n-\bar{m}\|_2+\|\Pi_n-\bar{\Pi}\|_2\\
&+\|\pi_n-\pi_*\|_2^2+\|\mu_n-\mu_*\|_2^2+\|m_n-\bar{m}\|_2^2+\|\Pi_n-\bar{\Pi}\|_2^2.
\end{aligned}
$$

Thus, we have obtained all three rates for bias, variance and variance estimation. ■

Using Lemma A15, we can now proceed to prove Theorem 2. Denote the out-of-k-fold estimators for nuisance models as $\widehat{\boldsymbol{\eta}}^{(\mathsf{k})}=(\widehat{\pi}^{(\mathsf{k})},\widehat{\mu}^{(\mathsf{k})},\widehat{\Pi}^{(\mathsf{k})},\widehat{m}^{(\mathsf{k})})$. We shall first establish the asymptotic approximation for the fold-k estimator

$$
\begin{aligned}
\widehat{\Delta}_{\mathsf{SMMAL}}^{(\mathsf{k})}=&\frac{K}{N}\sum_{i\in\mathcal{I}_k}\phi_{\mathsf{SSL,1}}(\mathbf{D}_i;\widehat{\boldsymbol{\eta}}^{(\mathsf{k})})-\phi_{\mathsf{SSL,0}}(\mathbf{D}_i;\widehat{\boldsymbol{\eta}}^{(\mathsf{k})})\\
=&\frac{K}{N}\sum_{i\in\mathcal{I}_k}\phi_{\mathsf{SSL,1}}(\mathbf{D}_i;\bar{\boldsymbol{\eta}})-\phi_{\mathsf{SSL,0}}(\mathbf{D}_i;\bar{\boldsymbol{\eta}})+o_p\left(n^{-1/2}\right).
\end{aligned}
$$

Then the asymptotic normally follows from the central limit theorem regarding the empirical mean term of the i.i.d. random variables. We will thereafter conclude the proof by showing the variance estimator is indeed consistent.

To establish the asymptotic expansion, we consider the decomposition

$$\begin{aligned}
&\widehat{\Delta}^{(\mathsf{k})}_{\mathsf{SMMAL}}\\
=&\frac{K}{N}\sum_{i\in\mathcal{I}_k}\phi_{\mathsf{SSL,1}}(\mathbf{D}_i;\bar{\boldsymbol{\eta}})-\phi_{\mathsf{SSL,0}}(\mathbf{D}_i;\bar{\boldsymbol{\eta}})\\
&+\frac{K}{N}\sum_{i\in\mathcal{I}_k}\left[\phi_{\mathsf{SSL,1}}(\mathbf{D}_i;\widehat{\boldsymbol{\eta}}^{(\mathsf{k})})-\phi_{\mathsf{SSL,1}}(\mathbf{D}_i;\bar{\boldsymbol{\eta}})-\mathbb{E}_{i\in\mathcal{I}_k}\{\phi_{\mathsf{SSL,1}}(\mathbf{D}_i;\widehat{\boldsymbol{\eta}}^{(\mathsf{k})})-\phi_{\mathsf{SSL,1}}(\mathbf{D}_i;\bar{\boldsymbol{\eta}})\mid\mathscr{D}_k^c\}\right]\\
&+\mathbb{E}_{i\in\mathcal{I}_k}\{\phi_{\mathsf{SSL,0}}(\mathbf{D}_i;\widehat{\boldsymbol{\eta}}^{(\mathsf{k})})-\phi_{\mathsf{SSL,0}}(\mathbf{D}_i;\bar{\boldsymbol{\eta}})\mid\mathscr{D}_k^c\}\\
&-\frac{K}{N}\sum_{i\in\mathcal{I}_k}\left[\phi_{\mathsf{SSL,0}}(\mathbf{D}_i;\widehat{\boldsymbol{\eta}}^{(\mathsf{k})})-\phi_{\mathsf{SSL,0}}(\mathbf{D}_i;\bar{\boldsymbol{\eta}})-\mathbb{E}_{i\in\mathcal{I}_k}\{\phi_{\mathsf{SSL,0}}(\mathbf{D}_i;\widehat{\boldsymbol{\eta}}^{(\mathsf{k})})-\phi_{\mathsf{SSL,0}}(\mathbf{D}_i;\bar{\boldsymbol{\eta}})\mid\mathscr{D}_k^c\}\right]\\
&-\mathbb{E}_{i\in\mathcal{I}_k}\{\phi_{\mathsf{SSL,0}}(\mathbf{D}_i;\widehat{\boldsymbol{\eta}}^{(\mathsf{k})})-\phi_{\mathsf{SSL,0}}(\mathbf{D}_i;\bar{\boldsymbol{\eta}})\mid\mathscr{D}_k^c\}. \qquad (A.26)
\end{aligned}$$

We can now apply Lemma A15 along with Assumption 2d to get

$$\begin{aligned}
&\mathrm{Var}_{i\in\mathcal{I}_k}\{\phi_{\mathsf{SSL,a}}(\mathbf{D}_i;\widehat{\boldsymbol{\eta}}^{(\mathsf{k})})-\phi_{\mathsf{SSL,a}}(\mathbf{D}_i;\bar{\boldsymbol{\eta}})\mid\mathscr{D}_k^c\}=o_p\left(1/\rho_{\mathsf{N}}\right),\\
&\mathbb{E}_{i\in\mathcal{I}_k}\{\phi_{\mathsf{SSL,a}}(\mathbf{D}_i;\widehat{\boldsymbol{\eta}}^{(\mathsf{k})})-\phi_{\mathsf{SSL,a}}(\mathbf{D}_i;\bar{\boldsymbol{\eta}})\mid\mathscr{D}_k^c\}=o_p\left(n^{-1/2}\right). \qquad (A.27)
\end{aligned}$$

Therefore, by the Tchebychev's inequality and the fact that $\rho_{\mathsf{N}}N=n$, we have

$$\begin{aligned}
&\frac{K}{N}\sum_{i\in\mathcal{I}_k}\left[\phi_{\mathsf{SSL,a}}(\mathbf{D}_i;\widehat{\boldsymbol{\eta}}^{(\mathsf{k})})-\phi_{\mathsf{SSL,a}}(\mathbf{D}_i;\bar{\boldsymbol{\eta}})-\mathbb{E}_{i\in\mathcal{I}_k}\{\phi_{\mathsf{SSL,a}}(\mathbf{D}_i;\widehat{\boldsymbol{\eta}}^{(\mathsf{k})})-\phi_{\mathsf{SSL,a}}(\mathbf{D}_i;\bar{\boldsymbol{\eta}})\mid\mathscr{D}_k^c\}\right]\\
=&o_p\left(n^{-1/2}\right). \qquad (A.28)
\end{aligned}$$

Thereafter combining (A.27) and (A.28) to (A.26), we have

$$\widehat{\Delta}^{(\mathsf{k})}_{\mathsf{SMMAL}}=\frac{K}{N}\sum_{i\in\mathcal{I}_k}\phi_{\mathsf{SSL,1}}(\mathbf{D}_i;\bar{\boldsymbol{\eta}})-\phi_{\mathsf{SSL,0}}(\mathbf{D}_i;\bar{\boldsymbol{\eta}})+o_p\left(n^{-1/2}\right).$$

Summing over all the folds, we obtain

$$\widehat{\Delta}_{\mathsf{SMMAL}}=\frac{1}{K}\sum_{k=1}^{K}\widehat{\Delta}^{(\mathsf{k})}_{\mathsf{SMMAL}}=\frac{1}{N}\sum_{i=1}^{N}\phi_{\mathsf{SSL,1}}(\mathbf{D}_i;\bar{\boldsymbol{\eta}})-\phi_{\mathsf{SSL,0}}(\mathbf{D}_i;\bar{\boldsymbol{\eta}})+o_p\left(n^{-1/2}\right). \qquad (A.29)$$

Using (A.25) along with Assumptions 2a and 2b, we therefore obtain that the variance of each summand in (A.29) scales with $1/\rho_{\mathsf{N}}$ as,

$$\mathrm{Var}\{\phi_{\mathsf{SSL,1}}(\mathbf{D}_i;\bar{\boldsymbol{\eta}})-\phi_{\mathsf{SSL,0}}(\mathbf{D}_i;\bar{\boldsymbol{\eta}})\}=\mathcal{V}_*/\rho_{\mathsf{N}}+O(1).$$

Under Assumption 2e, we can then scale it to obtain a stable variance

$$\mathcal{V}_{\mathsf{SMMAL}}=\mathrm{Var}\{\sqrt{\rho_{\mathsf{N}}}\phi_{\mathsf{SSL,1}}(\mathbf{D}_i;\bar{\boldsymbol{\eta}})-\sqrt{\rho_{\mathsf{N}}}\phi_{\mathsf{SSL,0}}(\mathbf{D}_i;\bar{\boldsymbol{\eta}})\}=\mathcal{V}_*+O(\rho_{\mathsf{N}})\in[1/2M,2M] \qquad (A.30)$$

for sufficiently small ρ_{N}. Applying the central limit theorem at $\sqrt{n}$-scale, we have

$$\sqrt{n}(\widehat{\Delta}_{\mathsf{SMMAL}} - \Delta_*) = \frac{1}{\sqrt{N}} \sum_{i=1}^{N} \sqrt{\rho_{\mathsf{N}}}\phi_{\mathsf{SSL},1}(\mathbf{D}_i; \bar{\boldsymbol{\eta}}) - \sqrt{\rho_{\mathsf{N}}}\phi_{\mathsf{SSL},0}(\mathbf{D}_i; \bar{\boldsymbol{\eta}}) + o_p(1) \rightsquigarrow N(0, \mathcal{V}_{\mathsf{SMMAL}}) \tag{A.31}$$

Finally, we can use Lemma A15 again to show the consistency of the variance estimator. To this end, we decompose the variance estimator as

$$\begin{aligned}
\widehat{\mathcal{V}}_{\mathsf{SMMAL}} =& \frac{1}{N} \sum_{k=1}^{K} \sum_{i \in \mathcal{I}_k} \rho_{\mathsf{N}} \{\phi_{\mathsf{SSL},1}(\mathbf{D}_i; \widehat{\boldsymbol{\eta}}^{(\mathsf{k})}) - \phi_{\mathsf{SSL},0}(\mathbf{D}_i; \widehat{\boldsymbol{\eta}}^{(\mathsf{k})})\}^2 - \rho_{\mathsf{N}} \widehat{\Delta}^2_{\mathsf{SMMAL}} \\
=& \mathcal{V}_{\mathsf{SMMAL}} + \rho_{\mathsf{N}}(\Delta_*^2 - \widehat{\Delta}^2_{\mathsf{SMMAL}}) \\
&+ \frac{1}{N} \sum_{k=1}^{K} \sum_{i \in \mathcal{I}_k} \rho_{\mathsf{N}} \{\phi_{\mathsf{SSL},1}(\mathbf{D}_i; \widehat{\boldsymbol{\eta}}^{(\mathsf{k})}) - \phi_{\mathsf{SSL},0}(\mathbf{D}_i; \widehat{\boldsymbol{\eta}}^{(\mathsf{k})})\}^2 \\
&\quad - \mathbb{E}_{i \in \mathcal{I}_k}[\rho_{\mathsf{N}} \{\phi_{\mathsf{SSL},1}(\mathbf{D}_i; \widehat{\boldsymbol{\eta}}^{(\mathsf{k})}) - \phi_{\mathsf{SSL},0}(\mathbf{D}_i; \widehat{\boldsymbol{\eta}}^{(\mathsf{k})})\}^2 \mid \mathscr{D}_k^c] \\
&+ \frac{1}{K} \sum_{k=1}^{K} \mathbb{E}_{i \in \mathcal{I}_k}[\rho_{\mathsf{N}} \{\phi_{\mathsf{SSL},1}(\mathbf{D}_i; \widehat{\boldsymbol{\eta}}^{(\mathsf{k})}) - \phi_{\mathsf{SSL},0}(\mathbf{D}_i; \widehat{\boldsymbol{\eta}}^{(\mathsf{k})})\}^2 \mid \mathscr{D}_k^c] \\
&\quad - \mathbb{E}[\rho_{\mathsf{N}} \{\phi_{\mathsf{SSL},1}(\mathbf{D}_i; \bar{\boldsymbol{\eta}}) - \phi_{\mathsf{SSL},0}(\mathbf{D}_i; \bar{\boldsymbol{\eta}})\}^2 \mid \mathscr{D}_k^c].
\end{aligned} \tag{A.32}$$

By the asymptotic normality of $\widehat{\Delta}_{\mathsf{SMMAL}}$ and the boundedness of Δ_* from Assumption 2a, we have

$$\rho_{\mathsf{N}}(\Delta_*^2 - \widehat{\Delta}^2_{\mathsf{SMMAL}}) = O_p\left(\rho_{\mathsf{N}} n^{-1/2}\right). \tag{A.33}$$

We denote the labeled data component and unlabeled data component of $\phi_{\mathsf{SSL},1} - \phi_{\mathsf{SSL},0}$ as

$$\begin{aligned}
\psi_1(\mathbf{D}; \boldsymbol{\eta}) =& \mu(1, \mathbf{X}) + \frac{\Pi(1, \mathbf{X})}{\pi(1, \mathbf{X})} \{m(1, \mathbf{W}) - \mu(1, \mathbf{X})\} \\
&- \mu(0, \mathbf{X}) - \frac{\Pi(0, \mathbf{X})}{\pi(0, \mathbf{X})} \{m(0, \mathbf{W}) - \mu(0, \mathbf{X})\} \\
\psi_2(\mathbf{D}; \boldsymbol{\eta}) =& \frac{\mathrm{I}(A=1)}{\pi(1, \mathbf{X})} \{Y - \mu(1, \mathbf{X})\} - \frac{\Pi(1, \mathbf{X})}{\pi(1, \mathbf{X})} \{m(1, \mathbf{W}) - \mu(1, \mathbf{X})\} \\
&- \frac{\mathrm{I}(A=0)}{\pi(0, \mathbf{X})} \{Y - \mu(0, \mathbf{X})\} + \frac{\Pi(0, \mathbf{X})}{\pi(0, \mathbf{X})} \{m(0, \mathbf{W}) - \mu(0, \mathbf{X})\}.
\end{aligned}$$

Using the identity $R_i^2 = R_i$, we express the term $\rho_{\mathsf{N}}\{\phi_{\mathsf{SSL},1}(\mathbf{D}_i; \widehat{\boldsymbol{\eta}}^{(\mathsf{k})}) - \phi_{\mathsf{SSL},0}(\mathbf{D}_i; \widehat{\boldsymbol{\eta}}^{(\mathsf{k})})\}^2$ in the generic form analyzed in Lemma A19

$$\begin{aligned}
&\rho_{\mathsf{N}} \{\phi_{\mathsf{SSL},1}(\mathbf{D}_i; \widehat{\boldsymbol{\eta}}^{(\mathsf{k})}) - \phi_{\mathsf{SSL},0}(\mathbf{D}_i; \widehat{\boldsymbol{\eta}}^{(\mathsf{k})})\}^2 \\
=& \rho_{\mathsf{N}} \left\{\psi_1(\mathbf{D}_i; \widehat{\boldsymbol{\eta}}^{(\mathsf{k})}) + \frac{R_i}{\rho_{\mathsf{N}}} \psi_2(\mathbf{D}_i; \widehat{\boldsymbol{\eta}}^{(\mathsf{k})}\right\}^2 \\
=& \underbrace{\rho_{\mathsf{N}} \psi_1(\mathbf{D}_i; \widehat{\boldsymbol{\eta}}^{(\mathsf{k})})^2 + 2R_i \psi_1(\mathbf{D}_i; \widehat{\boldsymbol{\eta}}^{(\mathsf{k})}) \psi_2(\mathbf{D}_i; \widehat{\boldsymbol{\eta}}^{(\mathsf{k})})}_{h_1 \text{ for Lemma A19}} + \frac{R_i}{\rho_{\mathsf{N}}} \underbrace{\psi_2(\mathbf{D}_i; \widehat{\boldsymbol{\eta}}^{(\mathsf{k})})^2}_{h_2 \text{ for Lemma A19}}.
\end{aligned}$$

Under Assumptions 2a and 2c, the h_1 and h_2 components are all bounded, so we can apply the concentration result in Lemma A19 to get

$$\frac{1}{N}\sum_{k=1}^{K}\sum_{i\in\mathcal{I}_k}\rho_{\mathsf{N}}\{\phi_{\mathsf{SSL},1}(\mathbf{D}_i;\widehat{\boldsymbol{\eta}}^{(\mathsf{k})})-\phi_{\mathsf{SSL},0}(\mathbf{D}_i;\widehat{\boldsymbol{\eta}}^{(\mathsf{k})})\}^2 \\ -\mathbb{E}_{i\in\mathcal{I}_k}[\rho_{\mathsf{N}}\{\phi_{\mathsf{SSL},1}(\mathbf{D}_i;\widehat{\boldsymbol{\eta}}^{(\mathsf{k})})-\phi_{\mathsf{SSL},0}(\mathbf{D}_i;\widehat{\boldsymbol{\eta}}^{(\mathsf{k})})\}^2\mid\mathscr{D}_k^c]=O_p\left(n^{-1/2}\right). \tag{A.34}$$

Also, applying Lemma A15 with Assumption 2d, we have

$$\frac{1}{N}\sum_{k=1}^{K}\sum_{i\in\mathcal{I}_k}\mathbb{E}_{i\in\mathcal{I}_k}[\rho_{\mathsf{N}}\{\phi_{\mathsf{SSL},1}(\mathbf{D}_i;\widehat{\boldsymbol{\eta}}^{(\mathsf{k})})-\phi_{\mathsf{SSL},0}(\mathbf{D}_i;\widehat{\boldsymbol{\eta}}^{(\mathsf{k})})\}^2\mid\mathscr{D}_k^c] \\ -\mathbb{E}[\rho_{\mathsf{N}}\{\phi_{\mathsf{SSL},1}(\mathbf{D}_i;\bar{\boldsymbol{\eta}})-\phi_{\mathsf{SSL},0}(\mathbf{D}_i;\bar{\boldsymbol{\eta}})\}^2\mid\mathscr{D}_k^c]=o_p(1). \tag{A.35}$$

Putting the rates (A.33)-(A.35) to (A.32), we have

$$\widehat{\mathcal{V}}_{\mathsf{SMMAL}}=\mathcal{V}_{\mathsf{SMMAL}}+o_p(1). \tag{A.36}$$

With the asymptotic normality (A.31), stable variance (A.30) and consistent variance estimator (A.36), we apply the continuous mapping theorem to get

$$\sqrt{n/\widehat{\mathcal{V}}_{\mathsf{SMMAL}}}(\widehat{\Delta}_{\mathsf{SMMAL}}-\Delta_*)\rightsquigarrow N(0,1).$$

C2 Proof of Theorem 5

Here we directly apply the conclusion of Theorem 13 by verifying Assumption 5. See the proof of Theorem 13 for details. By the Theorem 2.2 Setting IV of Kallus and Mao (2024), the efficient influence function for Δ_* under complete data is ϕ_{cmp}. Now, we verify each item in Assumption 5.

(a) We assume missing completely at random (1) throughout;

(b) We assume the stable variance from labeled portion as Assumption 2e;

(c) We are considering nonparametric model, so $\mathscr{H}$ contains all the mean zero square integrable random variables;

(d) The complete data efficient influence function ϕ_{cmp} is bounded under Assumptions 2a and 2b.

C3 Proof of Corollary 7

We verify the Assumption 2 using existing results for B-spline regression summarized in Lemma A24 (see Newey and Robins, 2018, for example). Under (1), Assumptions 3a and 2b, the densities of $\mathbf{X}\mid R=1$, $\mathbf{X}\mid R=1,A=1$, $\mathbf{X}\mid R=1,A=0$, $\mathbf{W}\mid R=1$, $\mathbf{W}\mid R=1,A=1$ and $\mathbf{W}\mid R=1,A=0$ are all bounded and bounded away from zero. We have the boundedness of Y from Assumption 2a, and A is naturally bounded by one. Under Assumption 3b, all nuisance models are Hölder smooth. Choosing tensor B-splines

with equally spaced knots and proper normalization, B-spline regressions for π, μ, Π and m all satisfy the conditions of Lemma A24.

Apply Lemma A24 with the order κ and degrees from the statement of Corollary 7, we have

$$\|\widehat{\pi}^{(\mathsf{k})}(a,\cdot)-\pi_*(a,\cdot)\|_2 = O_p\left(n^{-\frac{\mathcal{H}(\pi_*(a,\cdot))/p}{1+\mathcal{H}(\pi_*(a,\cdot))/p}}\right),\ \|\widehat{\mu}^{(\mathsf{k})}(a,\cdot)-\mu_*(a,\cdot)\|_2 = O_p\left(n^{-\frac{\mathcal{H}(\mu_*(a,\cdot))/p}{1+\mathcal{H}(\mu_*(a,\cdot))/p}}\right),$$
$$\|\widehat{\Pi}^{(\mathsf{k})}(a,\cdot)-\pi_*(a,\cdot)\|_2 = O_p\left(n^{-\frac{\mathcal{H}(\pi_*(a,\cdot))/p}{1+\mathcal{H}(\Pi_*(a,\cdot))/p}}\right),\ \|\widehat{m}^{(\mathsf{k})}(a,\cdot)-\mu_*(a,\cdot)\|_2 = O_p\left(n^{-\frac{\mathcal{H}(\mu_*(a,\cdot))/p}{1+\mathcal{H}(m_*(a,\cdot))/p}}\right).$$

Under Assumptions 2a and 2b, truncation at M does not increase estimation error,

$$\|\min\{M,\widehat{\pi}^{(\mathsf{k})}(a,\cdot)\}-\pi_*(a,\cdot)\|_2 = O_p\left(n^{-\frac{\mathcal{H}(\pi_*(a,\cdot))/p}{1+\mathcal{H}(\pi_*(a,\cdot))/p}}\right),$$
$$\|\min\{M,\widehat{\mu}^{(\mathsf{k})}(a,\cdot)\}-\mu_*(a,\cdot)\|_2 = O_p\left(n^{-\frac{\mathcal{H}(\mu_*(a,\cdot))/p}{1+\mathcal{H}(\mu_*(a,\cdot))/p}}\right),$$
$$\|\min\{M,\widehat{\Pi}^{(\mathsf{k})}(a,\cdot)\}-\pi_*(a,\cdot)\|_2 = O_p\left(n^{-\frac{\mathcal{H}(\pi_*(a,\cdot))/p}{1+\mathcal{H}(\Pi_*(a,\cdot))/p}}\right),$$
$$\|\min\{M,\widehat{m}^{(\mathsf{k})}(a,\cdot)\}-\mu_*(a,\cdot)\|_2 = O_p\left(n^{-\frac{\mathcal{H}(\mu_*(a,\cdot))/p}{1+\mathcal{H}(m_*(a,\cdot))/p}}\right).$$

The truncation at M secures Assumption 2c. Under Assumptions 2b and 3b, the rates for the truncated estimators above satisfy Assumption 2d with $\bar{\Pi}=\Pi_*$ and $\bar{m}=m_*$. Therefore, we can apply the asymptotic normality (A.31) from Theorem 2 to get

$$\sqrt{n}(\widehat{\Delta}_{\mathsf{SSL}}-\Delta_*) \rightsquigarrow N(0,\rho_{\mathsf{N}}\operatorname{Var}\{\phi_{\mathsf{SSL}}(RY,RA,\mathbf{W},R)\}).$$

C4 Proof of Theorem 8

In Section D1, we developed the estimation rates for Lasso estimators defined in (9)-(12) in Lemma A21. Two estimation rates from Lemma A21 corresponds to the two situation: 1) the general case in which the cross-fitted parameter must be consistent to identify the target parameter; 2) the special case in which the cross-fitted parameter does not exist or is not needed for identifying the target parameter. The special case applies to the imputation Lasso (9), the initial Lasso (10) and the calibrated Lasso (12) if the underlying model is correct. The general case applies to the calibrated Lasso (12) if the underlying model is wrong. We do not require any concentration of the initial estimator for the mis-specified model following the truncation of the weights in the calibrated Lasso (12). The proofs for Lemma A21 are based on the technique developed in Hou et al. (2021b). In summary, we obtain

1. Both models correct: $\boldsymbol{\alpha}_*=\bar{\boldsymbol{\alpha}}_{\mathsf{init}}=\bar{\boldsymbol{\alpha}}_{\mathsf{a}}$ and $\boldsymbol{\beta}_*=\bar{\boldsymbol{\beta}}_{\mathsf{a,init}}=\bar{\boldsymbol{\beta}}_{\mathsf{a}}$,

$$\|\widehat{\boldsymbol{\alpha}}_{\mathsf{a}}^{(\mathsf{k})}-\boldsymbol{\alpha}_*\|_2 = O_p\left(\sqrt{\|\boldsymbol{\alpha}_*\|_0\log(p)/n}\right),\ \|\widehat{\boldsymbol{\beta}}_{\mathsf{a}}^{(\mathsf{k})}-\boldsymbol{\beta}_{*,\mathsf{a}}\|_2 = O_p\left(\sqrt{\|\boldsymbol{\beta}_{*,\mathsf{a}}\|_0\log(p)/n}\right),$$
$$\|\widehat{\boldsymbol{\xi}}^{(\mathsf{k})}-\bar{\boldsymbol{\xi}}\|_2 = O_p\left(\sqrt{\|\bar{\boldsymbol{\xi}}\|_0\log(p+q)/n}\right),\ \|\widehat{\boldsymbol{\zeta}}_{\mathsf{a}}^{(\mathsf{k})}-\bar{\boldsymbol{\zeta}}_{\mathsf{a}}\|_2 = O_p\left(\sqrt{\|\bar{\boldsymbol{\zeta}}_{\mathsf{a}}\|_0\log(p+q)/n}\right).$$

2. PS model correct: $\boldsymbol{\alpha}_* = \bar{\boldsymbol{\alpha}}_{\mathsf{init}} = \bar{\boldsymbol{\alpha}}_{\mathsf{a}}$,

$$\|\widehat{\boldsymbol{\alpha}}_{\mathsf{a}}^{(\mathsf{k})} - \boldsymbol{\alpha}_*\|_2 = O_p\left(\sqrt{\|\boldsymbol{\alpha}_*\|_0 \log(p)/n}\right),$$
$$\|\widehat{\boldsymbol{\beta}}_{\mathsf{a}}^{(\mathsf{k})} - \bar{\boldsymbol{\beta}}_{\mathsf{a}}\|_2 = O_p\left(\sqrt{\left(\|\bar{\boldsymbol{\beta}}_{\mathsf{a}}\|_0 + \|\boldsymbol{\alpha}_*\|_0\right)\log(p)/n}\right),$$
$$\|\widehat{\boldsymbol{\xi}}^{(\mathsf{k})} - \bar{\boldsymbol{\xi}}\|_2 = O_p\left(\sqrt{\|\bar{\boldsymbol{\xi}}\|_0 \log(p+q)/n}\right),\ \|\widehat{\boldsymbol{\zeta}}_{\mathsf{a}}^{(\mathsf{k})} - \bar{\boldsymbol{\zeta}}_{\mathsf{a}}\|_2 = O_p\left(\sqrt{\|\bar{\boldsymbol{\zeta}}_{\mathsf{a}}\|_0 \log(p+q)/n}\right).$$

3. OR model correct: $\boldsymbol{\beta}_* = \bar{\boldsymbol{\beta}}_{\mathsf{a,init}} = \bar{\boldsymbol{\beta}}_{\mathsf{a}}$,

$$\|\widehat{\boldsymbol{\alpha}}_{\mathsf{a}}^{(\mathsf{k})} - \bar{\boldsymbol{\alpha}}_{\mathsf{a}}\|_2 = O_p\left(\sqrt{\left(\|\boldsymbol{\beta}_{*,\mathsf{a}}\|_0 + \|\bar{\boldsymbol{\alpha}}_{\mathsf{a}}\|_0\right)\log(p)/n}\right),$$
$$\|\widehat{\boldsymbol{\beta}}_{\mathsf{a}}^{(\mathsf{k})} - \boldsymbol{\beta}_{*,\mathsf{a}}\|_2 = O_p\left(\sqrt{\|\boldsymbol{\beta}_{*,\mathsf{a}}\|_0 \log(p)/n}\right),$$
$$\|\widehat{\boldsymbol{\xi}}^{(\mathsf{k})} - \bar{\boldsymbol{\xi}}\|_2 = O_p\left(\sqrt{\|\bar{\boldsymbol{\xi}}\|_0 \log(p+q)/n}\right),\ \|\widehat{\boldsymbol{\zeta}}_{\mathsf{a}}^{(\mathsf{k})} - \bar{\boldsymbol{\zeta}}_{\mathsf{a}}\|_2 = O_p\left(\sqrt{\|\bar{\boldsymbol{\zeta}}_{\mathsf{a}}\|_0 \log(p+q)/n}\right).$$

Similar to the proof of Theorem 2, we group the limiting nuisance models (15) as

$$\begin{aligned}\bar{\boldsymbol{\eta}} = (\bar{\pi}, \bar{\mu}, \bar{\Pi}, \bar{m}),\ \bar{\pi}(a, \mathbf{X}) = a g(\bar{\boldsymbol{\alpha}}_1^\mathsf{T}\mathbf{X}) + (1-a) g(-\bar{\boldsymbol{\alpha}}_0^\mathsf{T}\mathbf{X}),\\ \bar{\mu}(a, \mathbf{X}) = g(\bar{\boldsymbol{\alpha}}_{\mathsf{a}}^\mathsf{T}\mathbf{X}),\ \bar{\Pi}(a, \mathbf{W}) = g(\bar{\boldsymbol{\xi}}^\mathsf{T}\mathbf{W}),\ \bar{m}(a, \mathbf{W}) = g(-\bar{\boldsymbol{\zeta}}_{\mathsf{a}}^\mathsf{T}\mathbf{W}).\end{aligned} \tag{A.37}$$

The L_2 estimation rates translate to the mean square error rate of the model estimators (13) by Lemma A20,

$$\begin{aligned}\|\widehat{\pi}^{(\mathsf{k})}(a,\cdot) - \bar{\pi}(a,\cdot)\|_2 \lesssim \|\widehat{\boldsymbol{\alpha}}_{\mathsf{a}}^{(\mathsf{k})} - \bar{\boldsymbol{\alpha}}_{\mathsf{a}}\|_2,\ \|\widehat{\mu}^{(\mathsf{k})}(a,\cdot) - \bar{\mu}(a,\cdot)\|_2 \lesssim \|\widehat{\boldsymbol{\beta}}_{\mathsf{a}}^{(\mathsf{k})} - \bar{\boldsymbol{\beta}}_{\mathsf{a}}\|_2,\\ \|\widehat{\Pi}^{(\mathsf{k})}(a,\cdot) - \bar{\Pi}(a,\cdot)\|_2 \lesssim \|\widehat{\boldsymbol{\xi}}^{(\mathsf{k})} - \bar{\boldsymbol{\xi}}\|_2,\ \|\widehat{m}^{(\mathsf{k})}(a,\cdot) - \bar{m}(a,\cdot)\|_2 \lesssim \|\widehat{\boldsymbol{\zeta}}_{\mathsf{a}}^{(\mathsf{k})} - \bar{\boldsymbol{\zeta}}_{\mathsf{a}}\|_2.\end{aligned}$$

For the case of both models being correct in Assumption 4d-iii, we can directly apply Theorem 2. We study the other cases in which one of the PS or OR is correct in the rest of the proof.

We use the $\phi_{\mathsf{SSL,a}}(\mathbf{D};\boldsymbol{\eta})$ notation defined in (A.21). Notice that

$$\begin{aligned}\mathbb{E}\{\phi_{\mathsf{SSL,1}}(\mathbf{D};\bar{\boldsymbol{\eta}}) - \phi_{\mathsf{SSL,0}}(\mathbf{D};\bar{\boldsymbol{\eta}})\} =& \mathbb{E}\left[g(\mathbf{X}^\mathsf{T}\bar{\boldsymbol{\beta}}_1) + \frac{A}{g(\mathbf{X}^\mathsf{T}\bar{\boldsymbol{\alpha}}_1)}\{Y - g(\mathbf{X}^\mathsf{T}\bar{\boldsymbol{\beta}}_1)\}\right]\\ &- \mathbb{E}\left[g(\mathbf{X}^\mathsf{T}\bar{\boldsymbol{\beta}}_0) + \frac{1-A}{g(-\mathbf{X}^\mathsf{T}\bar{\boldsymbol{\alpha}}_0)}\{Y - g(\mathbf{X}^\mathsf{T}\bar{\boldsymbol{\beta}}_0)\}\right]\end{aligned}$$

so $\Delta_* = \mathbb{E}\{\phi_{\mathsf{SSL,1}}(\mathbf{D};\bar{\boldsymbol{\eta}}) - \phi_{\mathsf{SSL,0}}(\mathbf{D};\bar{\boldsymbol{\eta}})\}$ if either the PS or OR is correct (Bang and Robins, 2005). We state a modified version of Lemma A15.

Lemma A16 *Let $\boldsymbol{\eta}_n = (\pi_n, \mu_n, \Pi_n, m_n)$ be a (deterministic) sequence of nuisance models satisfying almost surely*

$$\sup_{a=0,1} \max\left\{|1/\pi_n(a, \mathbf{X}_i)|, |\mu_n(a, \mathbf{X}_i)|, |\Pi_n(a, \mathbf{W}_i)|, |m_n(a, \mathbf{W}_i)|\right\} \le M. \tag{A.38}$$

Define

$$\Psi_1(\mu_n) = \mathbb{E}\left[\{\mu_n(a,\mathbf{X}) - \bar{\mu}(a,\mathbf{X})\}\left\{\frac{\mathrm{I}(A=a)}{\bar{\pi}(a,\mathbf{X})} - 1\right\}\right],$$
$$\Psi_2(\pi_n) = \mathbb{E}\left[\left\{\frac{1}{\pi_n(a,\mathbf{X})} - \frac{1}{\bar{\pi}(a,\mathbf{X})}\right\}\mathrm{I}(A=a)\{Y - \bar{\mu}(a,\mathbf{X})\}\right]. \tag{A.39}$$

Under Assumptions 2a and 2b, we have

1. *For bias:*

$$|\mathbb{E}\{\phi_{\mathsf{SSL,a}}(\mathbf{D};\boldsymbol{\eta}_n) - \phi_{\mathsf{SSL,a}}(\mathbf{D};\bar{\boldsymbol{\eta}})\}| \lesssim \|\pi_n - \bar{\pi}\|_2\|\mu_n - \bar{\mu}\|_2 + \Psi_1(\mu_n) + \Psi_2(\pi_n),$$

2. *For variance:*

$$\begin{aligned}&\rho_{\mathsf{N}}\operatorname{Var}\{\phi_{\mathsf{SSL,a}}(\mathbf{D};\boldsymbol{\eta}_n) - \phi_{\mathsf{SSL,a}}(\mathbf{D};\bar{\boldsymbol{\eta}})\}\\ \lesssim&\|\pi_n - \bar{\pi}\|_2^2 + \|\mu_n - \bar{\mu}\|_2^2 + \|m_n - \bar{m}\|_2^2 + \|\Pi_n - \bar{\Pi}\|_2^2,\end{aligned}$$

3. *For variance estimation:*

$$\begin{aligned}&\mathbb{E}\left\{\rho_{\mathsf{N}}\phi_{\mathsf{SSL,a}}(\mathbf{D};\boldsymbol{\eta}_n)^2\right\} - \mathbb{E}\left\{\rho_{\mathsf{N}}\phi_{\mathsf{SSL,a}}(\mathbf{D};\bar{\boldsymbol{\eta}})^2\right\}\\ \lesssim&\|\pi_n - \bar{\pi}\|_2 + \|\mu_n - \bar{\mu}\|_2 + \|m_n - \bar{m}\|_2 + \|\Pi_n - \bar{\Pi}\|_2\\ &+ \|\pi_n - \bar{\pi}\|_2^2 + \|\mu_n - \bar{\mu}\|_2^2 + \|m_n - \bar{m}\|_2^2 + \|\Pi_n - \bar{\Pi}\|_2^2.\end{aligned}$$

We omit the proof of Lemma A16 as it merely repeats that of Lemma A15. The only difference is that limiting models $\bar{\pi}$ and $\bar{\mu}$ can deviate from the truth π_* and μ_*, so we have the extra terms (A.39) in the bias representation. In the next lemma, we study (A.39) under Assumption 4d-i or 4d-ii.

Lemma A17 *Let π_n and μ_n be the logistic regression predictions*

$$\pi_n(a,\mathbf{X}) = g_\tau((-1)^{a+1}\mathbf{X}^\intercal\boldsymbol{\alpha}_n),\ \mu_n(a,\mathbf{X}) = g((-1)^{a+1}\mathbf{X}^\intercal\boldsymbol{\beta}_n).$$

We have

1. *under Assumption 4d-i: for $\|\boldsymbol{\alpha}_n - \boldsymbol{\alpha}_*\|_2 \le 1/(2M)$,*

$$\Psi_1(\mu_n) = 0,\ \Psi_2(\pi_n) \lesssim \|\boldsymbol{\alpha}_n - \boldsymbol{\alpha}_*\|_2^2 + e^{-1/(2\|\boldsymbol{\alpha}_n - \boldsymbol{\alpha}_*\|_2^2)};$$

2. *under Assumption 4d-ii:*

$$\Psi_2(\pi_n) = 0,\ \Psi_1(\mu_n) \lesssim \|\boldsymbol{\beta}_n - \boldsymbol{\beta}_{*,\mathsf{a}}\|_2^2.$$

Proof [Proof of Lemma A17]
<u>PS correct:</u> We have $\bar{\boldsymbol{\alpha}}_{\mathsf{a}} = \boldsymbol{\alpha}_*$ and $\bar{\pi} = \pi_*$. As the result, we have

$$\Psi_1(\mu_n) = \mathbb{E}\left[\{\mu_n(a,\mathbf{X}) - \bar{\mu}(a,\mathbf{X})\}\frac{\mathbb{E}\{\mathrm{I}(A=a) - \pi_*(a,\mathbf{X}) \mid \mathbf{X}\}}{\pi_*(a,\mathbf{X})}\right] = 0.$$

In the following, we set $a = 1$, while the $a = 0$ case can be obtained by the same steps. To analyze $\Psi_2(\pi_n)$, we consider the following decomposition

$$\begin{aligned}\Psi_2(\pi_n) =&\mathbb{E}\left[\{\exp_\tau(-\mathbf{X}^\intercal\boldsymbol{\alpha}_n) - \exp(-\mathbf{X}^\intercal\boldsymbol{\alpha}_n)\} A\{Y - \bar{\mu}(1,\mathbf{X})\}\right] \\ &- (\boldsymbol{\alpha}_n - \boldsymbol{\alpha}_*)^\intercal\mathbb{E}\left[\exp(-\mathbf{X}^\intercal\boldsymbol{\alpha}_*)\mathbf{X}A\{Y - \bar{\mu}(1,\mathbf{X})\}\right] \\ &+ \frac{1}{2}(\boldsymbol{\alpha}_n - \boldsymbol{\alpha}_*)^\intercal\mathbb{E}\left[\exp(-\mathbf{X}^\intercal\widetilde{\boldsymbol{\alpha}})\mathbf{X}\mathbf{X}^\intercal A\{Y - \bar{\mu}(1,\mathbf{X})\}\right](\boldsymbol{\alpha}_n - \boldsymbol{\alpha}_*) \end{aligned} \tag{A.40}$$

for some $\widetilde{\boldsymbol{\alpha}}$ between $\boldsymbol{\alpha}_n$ and $\boldsymbol{\alpha}_*$. Under Assumption 4c, $|\mathbf{X}^\intercal\boldsymbol{\alpha}_*| \le M$ the truncation at $2M$ for $\mathbf{X}^\intercal\boldsymbol{\alpha}_n$ would only be triggered if $|\mathbf{X}^\intercal(\boldsymbol{\alpha}_n - \boldsymbol{\alpha}_*)| \ge M$. Thus, we may derive the following upper bound for the truncation error,

$$\begin{aligned}&\left|\mathbb{E}\left[\{\exp_\tau(-\mathbf{X}^\intercal\boldsymbol{\alpha}_n) - \exp(-\mathbf{X}^\intercal\boldsymbol{\alpha}_n)\} A\{Y - \bar{\mu}(1,\mathbf{X})\}\right]\right| \\ \le&\mathbb{E}\left[\exp(|\mathbf{X}^\intercal\boldsymbol{\alpha}_n|)\mathrm{I}(|\mathbf{X}^\intercal(\boldsymbol{\alpha}_n - \boldsymbol{\alpha}_*)| \ge M)\right] \\ \le&\mathbb{E}\left[e^M\exp(|\mathbf{X}^\intercal(\boldsymbol{\alpha}_n - \boldsymbol{\alpha}_*)|)\mathrm{I}(|\mathbf{X}^\intercal(\boldsymbol{\alpha}_n - \boldsymbol{\alpha}_*)| \ge M)\right] \\ \le&e^M\sqrt{\mathbb{E}\left\{\exp(2|\mathbf{X}^\intercal(\boldsymbol{\alpha}_n - \boldsymbol{\alpha}_*)|)\right\}\mathbb{P}(|\mathbf{X}^\intercal(\boldsymbol{\alpha}_n - \boldsymbol{\alpha}_*)| \ge M)} \\ \le&e^M\sqrt{\mathbb{E}\left\{\exp\left(\frac{|\mathbf{X}^\intercal(\boldsymbol{\alpha}_n - \boldsymbol{\alpha}_*)|}{M\|\boldsymbol{\alpha}_n - \boldsymbol{\alpha}_*\|_2}\right)\right\}\mathbb{P}(|\mathbf{X}^\intercal(\boldsymbol{\alpha}_n - \boldsymbol{\alpha}_*)| \ge M)}. \end{aligned} \tag{A.41}$$

The last inequality above follows from the assumption that $\|\boldsymbol{\alpha}_n - \boldsymbol{\alpha}_*\|_2 \le 1/(2M)$. Under Assumption 4a, $\mathbf{X}^\intercal(\boldsymbol{\alpha}_n - \boldsymbol{\alpha}_*)$ is sub-Gaussian thus sub-exponential

$$\|\mathbf{X}^\intercal(\boldsymbol{\alpha}_n - \boldsymbol{\alpha}_*)\|_{\psi_1} \le \|\mathbf{X}^\intercal(\boldsymbol{\alpha}_n - \boldsymbol{\alpha}_*)\|_{\psi_2} \le M\|\boldsymbol{\alpha}_n - \boldsymbol{\alpha}_*\|_2,$$

so we may apply the definition of sub-Gaussian/sub-exponential random variable and its property in tail probability to get

$$\mathbb{E}\left\{\exp\left(\frac{|\mathbf{X}^\intercal(\boldsymbol{\alpha}_n - \boldsymbol{\alpha}_*)|}{M\|\boldsymbol{\alpha}_n - \boldsymbol{\alpha}_*\|_2}\right)\right\} \le 2,\ \mathbb{P}(|\mathbf{X}^\intercal(\boldsymbol{\alpha}_n - \boldsymbol{\alpha}_*)| \ge M) \le 2e^{-1/\|\boldsymbol{\alpha}_n - \boldsymbol{\alpha}_*\|_2^2},. \tag{A.42}$$

Applying (A.42) to (A.41), we have

$$\left|\mathbb{E}\left[\{\exp_\tau(-\mathbf{X}^\intercal\boldsymbol{\alpha}_n) - \exp(-\mathbf{X}^\intercal\boldsymbol{\alpha}_n)\} A\{Y - \bar{\mu}(1,\mathbf{X})\}\right]\right| \lesssim e^{-1/(2\|\boldsymbol{\alpha}_n - \boldsymbol{\alpha}_*\|_2^2)}. \tag{A.43}$$

By the definition $\bar{\boldsymbol{\beta}}_{\mathsf{a}}$ (15), they must satisfy the first order condition of optimality

$$\mathbb{E}\left[\exp(-\mathbf{X}^\intercal\bar{\boldsymbol{\alpha}}_{\mathsf{init}})A\mathbf{X}\{Y - g(\mathbf{X}^\intercal\bar{\boldsymbol{\beta}}_1)\right] = 0. \tag{A.44}$$

By the definition of $\bar{\mu}$ and the fact $\bar{\boldsymbol{\alpha}}_{\mathsf{init}} = \boldsymbol{\alpha}_*$ under correct OR model, we infer from (A.44),

$$\mathbb{E}\left[\exp(-\mathbf{X}^\intercal\boldsymbol{\alpha}_*)\mathbf{X}A\{Y - \bar{\mu}(1,\mathbf{X})\}\right] = 0. \tag{A.45}$$

We bound the quadratic term in (A.40) with Assumptions 4a, 4c,

$$\begin{aligned}&\left|\frac{1}{2}(\boldsymbol{\alpha}_n - \boldsymbol{\alpha}_*)^\intercal\mathbb{E}\left[\exp(-\mathbf{X}^\intercal\widetilde{\boldsymbol{\alpha}})\mathbf{X}\mathbf{X}^\intercal A\{Y - \bar{\mu}(1,\mathbf{X})\}\right](\boldsymbol{\alpha}_n - \boldsymbol{\alpha}_*)\right| \\ \lesssim&\mathbb{E}[e^M\exp(|\mathbf{X}^\intercal(\boldsymbol{\alpha}_n - \boldsymbol{\alpha}_*)|)\{(\boldsymbol{\alpha}_n - \boldsymbol{\alpha}_*)^\intercal\mathbf{X}\}^2]\end{aligned} \tag{A.46}$$

$$
\begin{aligned}
&\lesssim e^{M}\sqrt{\mathbb{E}\left\{\exp\left(\frac{|\mathbf{X}^{\intercal}(\boldsymbol{\alpha}_n-\boldsymbol{\alpha}_*)|^2}{M^2\|\boldsymbol{\alpha}_n-\boldsymbol{\alpha}_*\|_2^2}\right)\right\}\mathbb{E}[\{(\boldsymbol{\alpha}_n-\boldsymbol{\alpha}_*)^{\intercal}\mathbf{X}\}^4]} \\
&\lesssim\|\boldsymbol{\alpha}_n-\boldsymbol{\alpha}_*\|_2^2. \qquad (A.47)
\end{aligned}
$$

We have used again the sub-Gaussian property of (A.42) in the last inequality. Applying A.43, (A.45) and (A.47) to (A.40), we have shown

$$
\Psi_2(\pi_n) \lesssim \|\boldsymbol{\alpha}_n-\boldsymbol{\alpha}_*\|_2^2 + e^{-1/(2\|\boldsymbol{\alpha}_n-\boldsymbol{\alpha}_*\|_2^2)}.
$$

<u>OR correct:</u> We have $\bar{\boldsymbol{\beta}}_{\mathsf{a}} = \boldsymbol{\beta}_{*,\mathsf{a}}$ and $\bar{\mu} = \mu_*$. As the result, we have

$$
\Psi_2(\pi_n) = \mathbb{E}\left[\left\{\frac{1}{\pi_n(a,\mathbf{X})} - \frac{1}{\bar{\pi}(a,\mathbf{X})}\right\}\mathrm{I}(A=a)\mathbb{E}\{Y-\mu_*(a,\mathbf{X}) \mid A,\mathbf{X}\}\right] = 0.
$$

Using the Mean Value Theorem on $\Psi_1(\mu_n)$, we have

$$
\begin{aligned}
\Psi_1(\mu_n) =&(\boldsymbol{\beta}_n-\boldsymbol{\beta}_{*,\mathsf{a}})^{\intercal}\mathbb{E}\left[\dot{g}(\mathbf{X}^{\intercal}\boldsymbol{\beta}_{*,\mathsf{a}})\mathbf{X}\left\{\frac{\mathrm{I}(A=a)}{\bar{\pi}(a,\mathbf{X})}-1\right\}\right] \\
&+\frac{1}{2}(\boldsymbol{\beta}_n-\boldsymbol{\beta}_{*,\mathsf{a}})^{\intercal}\mathbb{E}\left[g''(\mathbf{X}^{\intercal}\widetilde{\boldsymbol{\beta}})\mathbf{X}\mathbf{X}^{\intercal}\left\{\frac{\mathrm{I}(A=a)}{\bar{\pi}(a,\mathbf{X})}-1\right\}\right](\boldsymbol{\beta}_n-\boldsymbol{\beta}_{*,\mathsf{a}}) \qquad (A.48)
\end{aligned}
$$

for some $\widetilde{\boldsymbol{\beta}}$ between $\boldsymbol{\beta}_n$ and $\boldsymbol{\beta}_{*,\mathsf{a}}$. By the definition $\bar{\boldsymbol{\alpha}}_{\mathsf{a}}$ (15), they must satisfy the first order condition of optimality

$$
\begin{aligned}
\mathbb{E}\left[\dot{g}(\mathbf{X}^{\intercal}\bar{\boldsymbol{\beta}}_{1,\mathsf{init}})\{1-A(1+e^{-\mathbf{X}^{\intercal}\bar{\boldsymbol{\alpha}}_1})\}\right] &= 0, \\
\mathbb{E}\left[\dot{g}(\mathbf{X}^{\intercal}\bar{\boldsymbol{\beta}}_{0,\mathsf{init}})\{1-(1-A)(1+e^{\mathbf{X}^{\intercal}\bar{\boldsymbol{\alpha}}_0})\}\right] &= 0. \qquad (A.49)
\end{aligned}
$$

By the definition of $\bar{\pi}$ and the fact $\bar{\boldsymbol{\beta}}_{\mathsf{a},\mathsf{init}} = \boldsymbol{\beta}_{*,\mathsf{a}}$ under correct OR model, we infer from (A.49),

$$
\mathbb{E}\left[\dot{g}(\mathbf{X}^{\intercal}\boldsymbol{\beta}_{*,\mathsf{a}})\mathbf{X}\left\{\frac{\mathrm{I}(A=a)}{\bar{\pi}(a,\mathbf{X})}-1\right\}\right] = 0. \qquad (A.50)
$$

We bound the quadratic term in (A.48) with Assumptions 4a, 4c and bounds for $\|g''\|_\infty \leq 1/(6\sqrt{3})$,

$$
\begin{aligned}
\left|\frac{1}{2}(\boldsymbol{\beta}_n-\boldsymbol{\beta}_{*,\mathsf{a}})^{\intercal}\mathbb{E}\left[g''(\mathbf{X}^{\intercal}\widetilde{\boldsymbol{\beta}})\mathbf{X}\mathbf{X}^{\intercal}\left\{\frac{\mathrm{I}(A=a)}{\bar{\pi}(a,\mathbf{X})}-1\right\}\right](\boldsymbol{\beta}_n-\boldsymbol{\beta}_{*,\mathsf{a}})\right| &\lesssim\mathbb{E}[\{(\boldsymbol{\beta}_n-\boldsymbol{\beta}_{*,\mathsf{a}})^{\intercal}\mathbf{X}\}^2] \\
&\lesssim\|\boldsymbol{\beta}_n-\boldsymbol{\beta}_{*,\mathsf{a}}\|_2^2. \qquad (A.51)
\end{aligned}
$$

Applying (A.50) and (A.51) to (A.48), we have shown

$$
\Psi_1(\mu_n) \lesssim \|\boldsymbol{\beta}_n-\boldsymbol{\beta}_{*,\mathsf{a}}\|_2^2.
$$

■

As Lemma A17 require $\|\boldsymbol{\alpha}_n - \boldsymbol{\alpha}_*\|_2$ to be sufficiently small, we create the hypothetical error truncated estimators for $\widehat{\boldsymbol{\alpha}}_{\mathsf{a}}^{(\mathsf{k})}$,

$$\check{\boldsymbol{\alpha}}_{\mathsf{a}}^{(\mathsf{k})} = \bar{\boldsymbol{\alpha}}_{\mathsf{a}} + (\widehat{\boldsymbol{\alpha}}_{\mathsf{a}}^{(\mathsf{k})} - \bar{\boldsymbol{\alpha}}_{\mathsf{a}}) \min\left\{1, \frac{1}{M\|\widehat{\boldsymbol{\alpha}}_{\mathsf{a}}^{(\mathsf{k})} - \bar{\boldsymbol{\alpha}}_{\mathsf{a}}\|_2}\right\}. \tag{A.52}$$

Under Assumption 4d, we have $\|\widehat{\boldsymbol{\alpha}}_{\mathsf{a}}^{(\mathsf{k})} - \bar{\boldsymbol{\alpha}}_{\mathsf{a}}\|_2 = o_p(1)$, so $\check{\boldsymbol{\alpha}}_{\mathsf{a}}^{(\mathsf{k})} = \widehat{\boldsymbol{\alpha}}_{\mathsf{a}}^{(\mathsf{k})}$ with large probability. From here, we repeat the proof of Theorem 2 after Lemma A15 and obtain

$$\sqrt{n/\widehat{\mathcal{V}}_{\mathsf{DR}}}(\widehat{\Delta}_{\mathsf{DR}} - \Delta_*) \rightsquigarrow N(0,1).$$

C5 Proof of Proposition 12

The proof runs in two steps. First, we show that $\boldsymbol{\psi}_{\mathsf{SSL}}$ is an influence function for $\boldsymbol{\theta}$ under $\mathcal{S}_{\mathsf{SSL}}$ using the property of $\boldsymbol{\psi}_{\mathsf{cmp}}$ being the influence function for $\boldsymbol{\theta}$ under $\mathcal{S}_{\mathsf{cmp}}$. Second, we show that $\boldsymbol{\psi}_{\mathsf{SSL}}$ is efficient in the sense that it belongs to the tangent space of model $\mathcal{S}_{\mathsf{SSL}}$.

Consider a generic parametric sub-model for complete data and SSL data as follows

$$\begin{aligned}
\mathcal{S}_{\mathsf{cmp}}(\boldsymbol{\eta}) &= \{f(\mathbf{z}, \mathbf{w}; \boldsymbol{\eta}) : \boldsymbol{\eta} \in \mathbb{R}^p\} \subset \mathcal{S}_{\mathsf{cmp}}, \\
f_{\mathbf{W}}(\mathbf{w}; \boldsymbol{\eta}) &= \int f_{\mathbf{Z},\mathbf{W}}(\mathbf{z}, \mathbf{w}; \boldsymbol{\eta}) d\nu_z(\mathbf{z}), \\
\mathcal{S}_{\mathsf{SSL}}(\boldsymbol{\eta}) &= \left\{[\rho_{\mathsf{N}} f(\mathbf{z}, \mathbf{w}; \boldsymbol{\eta})]^r \left[(1-\rho_{\mathsf{N}}) f_{\mathbf{W}}(\mathbf{w}; \boldsymbol{\eta})\right]^{(1-r)} d\nu_{\mathsf{SSL}}(r, \mathbf{z}, \mathbf{w}) : \boldsymbol{\eta} \in \mathbb{R}^p\right\} \subset \mathcal{S}_{\mathsf{SSL}}.
\end{aligned} \tag{A.53}$$

The true model is attained at $f(\mathbf{z}, \mathbf{w}; \boldsymbol{\eta}^*) = f_*(\mathbf{z}, \mathbf{w})$. We denote the score function from the complete data as

$$\boldsymbol{\Psi}_{\mathsf{cmp}}(\mathbf{Z}, \mathbf{W}) = \frac{\partial}{\partial \boldsymbol{\eta}} \log\{f(\mathbf{Z}, \mathbf{W}; \boldsymbol{\eta})\}\bigg|_{\boldsymbol{\eta}=\boldsymbol{\eta}^*}. \tag{A.54}$$

The score function under the SSL model $\mathcal{S}_{\mathsf{SSL}}$ can be expressed as

$$\begin{aligned}
\boldsymbol{\Psi}_{\mathsf{SSL}}(R, \mathbf{Z}, \mathbf{W}) =& R\boldsymbol{\Psi}_{\mathsf{cmp}}(\mathbf{Z}, \mathbf{W}) + (1-R)\frac{\partial}{\partial \boldsymbol{\eta}} \log\{f_{\mathbf{W}}(\mathbf{W}; \boldsymbol{\eta})\}\bigg|_{\boldsymbol{\eta}=\boldsymbol{\eta}^*} \\
=& R\boldsymbol{\Psi}_{\mathsf{cmp}}(\mathbf{Z}, \mathbf{W}) + (1-R)\mathbb{E}_*\{\boldsymbol{\Psi}_{\mathsf{cmp}}(\mathbf{Z}, \mathbf{W}) \mid \mathbf{W}\}
\end{aligned} \tag{A.55}$$

Since $\boldsymbol{\psi}_{\mathsf{cmp}}$ is the influence function for $\boldsymbol{\theta}$, we must have

$$\mathbb{E}_*\{\boldsymbol{\psi}_{\mathsf{cmp}}(\mathbf{Z}, \mathbf{W})\boldsymbol{\Psi}_{\mathsf{cmp}}(\mathbf{Z}, \mathbf{W})\} = \frac{\partial}{\partial \boldsymbol{\eta}}\boldsymbol{\theta}(\boldsymbol{\eta})\bigg|_{\boldsymbol{\eta}=\boldsymbol{\eta}^*}. \tag{A.56}$$

Under Assumption 5a, we can calculate

$$\begin{aligned}
&\mathbb{E}_*\{\boldsymbol{\psi}_{\mathsf{SSL}}(R, \mathbf{Z}, \mathbf{W})\boldsymbol{\Psi}_{\mathsf{SSL}}(R, \mathbf{Z}, \mathbf{W})\} \\
=& \mathbb{E}_*\left\{\frac{R}{\rho_{\mathsf{N}}}\boldsymbol{\psi}_{\mathsf{cmp}}(\mathbf{Z}, \mathbf{W})\boldsymbol{\Psi}_{\mathsf{cmp}}(\mathbf{Z}, \mathbf{W})\right\} \\
&+ \mathbb{E}_*\left(\frac{R}{\rho_{\mathsf{N}}}\mathbb{E}_*\{\boldsymbol{\psi}_{\mathsf{cmp}}(\mathbf{Z}, \mathbf{W}) \mid \mathbf{W}\}\left[\boldsymbol{\Psi}_{\mathsf{cmp}}(\mathbf{Z}, \mathbf{W}) - \mathbb{E}_*\{\boldsymbol{\Psi}_{\mathsf{cmp}}(\mathbf{Z}, \mathbf{W}) \mid \mathbf{W}\}\right]\right)
\end{aligned}$$

$$
\begin{aligned}
&+ \mathbb{E}_* \left\{ (R/\rho_{\mathsf{N}} - 1)\mathbb{E}_*\{\boldsymbol{\psi}_{\mathsf{cmp}}(\mathbf{Z},\mathbf{W}) \mid \mathbf{W}\}\mathbb{E}_*\{\boldsymbol{\Psi}_{\mathsf{cmp}}(\mathbf{Z},\mathbf{W}) \mid \mathbf{W}\} \right\} \\
=&\mathbb{E}_* \left\{ \boldsymbol{\psi}_{\mathsf{cmp}}(\mathbf{Z},\mathbf{W})\boldsymbol{\Psi}_{\mathsf{cmp}}(\mathbf{Z},\mathbf{W}) \right\} \\
=& \frac{\partial}{\partial \boldsymbol{\eta}} \boldsymbol{\theta}(\boldsymbol{\eta}) \bigg|_{\boldsymbol{\eta}=\boldsymbol{\eta}^*}. \qquad (A.57)
\end{aligned}
$$

Thus, we verify that $\boldsymbol{\psi}_{\mathsf{SSL}}$ is an influence function for $\boldsymbol{\theta}$ under model $\mathcal{S}_{\mathsf{SSL}}$.

Now, we prove that $\boldsymbol{\psi}_{\mathsf{SSL}}$ belongs to the maximal nonparametric tangent space under model $\mathcal{S}_{\mathsf{SSL}}$. From the Assumption 5c and the efficient influence function $\boldsymbol{\psi}_{\mathsf{cmp}}$, we know two elements in the tangent space under model $\mathcal{S}_{\mathsf{cmp}}$,

$$
\boldsymbol{\psi}_{\mathsf{cmp}}(\mathbf{Z},\mathbf{W}),\ \mathbb{E}_*\{\boldsymbol{\psi}_{\mathsf{cmp}}(\mathbf{Z},\mathbf{W}) \mid \mathbf{W}\}. \qquad (A.58)
$$

According to the connection between the scores for $\mathcal{S}_{\mathsf{cmp}}$ and $\mathcal{S}_{\mathsf{SSL}}$ (A.55), we obtain two elements in the tangent space under model $\mathcal{S}_{\mathsf{SSL}}$,

$$
\begin{aligned}
\mathbf{U} &= R\left[\boldsymbol{\psi}_{\mathsf{cmp}}(\mathbf{Z},\mathbf{W}) - \mathbb{E}_*\{\boldsymbol{\psi}_{\mathsf{cmp}}(\mathbf{Z},\mathbf{W}) \mid \mathbf{W}\}\right] + \mathbb{E}_*\{\boldsymbol{\psi}_{\mathsf{cmp}}(\mathbf{Z},\mathbf{W}) \mid \mathbf{W}\}, \\
\mathbf{V} &= \mathbb{E}_*\{\boldsymbol{\psi}_{\mathsf{cmp}}(\mathbf{Z},\mathbf{W}) \mid \mathbf{W}\}. \qquad (A.59)
\end{aligned}
$$

Notice that we can express $\boldsymbol{\psi}_{\mathsf{SSL}}$ as the linear combination of the two elements above

$$
\boldsymbol{\psi}_{\mathsf{SSL}}(R,\mathbf{Z},\mathbf{W}) = \mathbf{U}/\rho_{\mathsf{N}} + \mathbf{V}(1 - 1/\rho_{\mathsf{N}}). \qquad (A.60)
$$

Since the tangent space is a linear space, $\boldsymbol{\psi}_{\mathsf{SSL}}$ must also be an element in the tangent space.

We have shown that $\boldsymbol{\psi}_{\mathsf{SSL}}$ is an element in the tangent space of $\mathcal{S}_{\mathsf{SSL}}$ satisfying (A.57). Therefore, $\boldsymbol{\psi}_{\mathsf{SSL}}$ is the efficient influence function for $\boldsymbol{\theta}$ under $\mathcal{S}_{\mathsf{SSL}}$.

C6 Proof of Theorem 13

Suppose the dimension of $\boldsymbol{\theta}$ is q. We start with the construction of the $2q$-dimensional least favorable model. From the Assumption 5c and the efficient influence function $\boldsymbol{\psi}_{\mathsf{cmp}}$, we know two elements in the nuisance parameter tangent space under model $\mathcal{S}_{\mathsf{SSL}}$,

$$
\boldsymbol{\psi}_{\mathsf{cmp}}(\mathbf{Z},\mathbf{W}),\ \mathbb{E}_*\{\boldsymbol{\psi}_{\mathsf{cmp}}(\mathbf{Z},\mathbf{W}) \mid \mathbf{W}\}. \qquad (A.61)
$$

We set the two tilt directions as

$$
\begin{aligned}
\mathbf{g}_1(\mathbf{Z},\mathbf{W}) &= \boldsymbol{\psi}_{\mathsf{cmp}}(\mathbf{Z},\mathbf{W}) - \mathbb{E}_*\{\boldsymbol{\psi}_{\mathsf{cmp}}(\mathbf{Z},\mathbf{W}) \mid \mathbf{W}\}, \\
\mathbf{g}_2(\mathbf{W}) &= \mathbb{E}_*\{\boldsymbol{\psi}_{\mathsf{cmp}}(\mathbf{Z},\mathbf{W}) \mid \mathbf{W}\}. \qquad (A.62)
\end{aligned}
$$

We denote the variances of two directions as

$$
\begin{aligned}
\mathcal{V}_{\psi,1} &= \mathrm{Var}_*\{\mathbf{g}_1(\mathbf{Z},\mathbf{W})\} = \mathbb{E}_*[\mathrm{Var}_*\{\mathbf{g}_1(\mathbf{Z},\mathbf{W})\}], \\
\mathcal{V}_{\psi,2} &= \mathrm{Var}_*\{\mathbf{g}_2(\mathbf{W})\} = \mathrm{Var}_*[\mathbb{E}_*\{\mathbf{g}_1(\mathbf{Z},\mathbf{W})\}], \\
\mathcal{V}_{\psi} &= \begin{pmatrix} \mathcal{V}_{\psi,1} & \mathbb{O}_q \\ \mathbb{O}_q & \mathcal{V}_{\psi,2} \end{pmatrix}. \qquad (A.63)
\end{aligned}
$$

Denote

$$
\left[1 + \frac{\mathbf{h}_1^{\intercal}\mathbf{g}_1(\mathbf{z}_i,\mathbf{w}_i)}{\sqrt{\rho_{\mathsf{N}} N}} + \frac{\mathbf{h}_2^{\intercal}\mathbf{g}_2(\mathbf{w}_i)}{\sqrt{N}}\right]_+ = \max\left\{0, 1 + \frac{\mathbf{h}_1^{\intercal}\mathbf{g}_1(\mathbf{z}_i,\mathbf{w}_i)}{\sqrt{\rho_{\mathsf{N}} N}} + \frac{\mathbf{h}_2^{\intercal}\mathbf{g}_2(\mathbf{w}_i)}{\sqrt{N}}\right\},
$$

$$C_{\mathbf{h}} = \mathbb{E}_* \left(\left[1 + \frac{\mathbf{h}_1^\intercal \mathbf{g}_1(\mathbf{Z}_i, \mathbf{W}_i)}{\sqrt{\rho_{\mathsf{N}} N}} + \frac{\mathbf{h}_2^\intercal \mathbf{g}_2(\mathbf{W}_i)}{\sqrt{N}} \right]_+ \right),$$

we construct the two-way tilted density as

$$f_{\mathbf{h}}(\mathbf{z}_i, \mathbf{w}_i) = f_*(\mathbf{z}_i, \mathbf{w}_i) \left[1 + \frac{\mathbf{h}_1^\intercal \mathbf{g}_1(\mathbf{z}_i, \mathbf{w}_i)}{\sqrt{\rho_{\mathsf{N}} N}} + \frac{\mathbf{h}_2^\intercal \mathbf{g}_2(\mathbf{w}_i)}{\sqrt{N}} \right]_+ / C_{\mathbf{h}}.$$

In Lemma A25, we proved that the two-way tilted density falls in the neighborhood in $\|\cdot\|_{\mathsf{TV}}$ under Assumption 5d.

$$\|f_* - f_{\mathbf{h}}\|_{\mathsf{TV}} \le M \sqrt{\|\mathbf{h}_1\|_2^2/\rho_{\mathsf{N}} N + \|\mathbf{h}_2\|_2^2/N} + o(\|\mathbf{h}\|_2/\sqrt{\rho_{\mathsf{N}} N}). \tag{A.64}$$

Thus for $\|\mathbf{h}_1\|_2^2 + \|\mathbf{h}_2\|_2^2 \le c^2$ and sufficiently large $\rho_{\mathsf{N}} N$ (as the expected number of labels grows asymptotically to infinity, $\rho_{\mathsf{N}} N \to \infty$), we have

$$\left\| f_* - f_* \left[1 + \frac{\mathbf{h}_1^\intercal \mathbf{g}_1}{\sqrt{\rho_{\mathsf{N}} N}} + \frac{\mathbf{h}_2^\intercal \mathbf{g}_2}{\sqrt{N}} \right]_+ \right\|_{\mathsf{TV}} \le 2Mc/\sqrt{\rho_{\mathsf{N}} N}.$$

We may consider the relaxed minimax problem to the $2q$-dimensional least favorable model:

$$aMSE = \liminf_{c\to\infty} \liminf_{N\to\infty} \sup_{\|\mathbf{h}_1\|_2^2 + \|\mathbf{h}_2\|_2^2 \le c^2} \int \rho_{\mathsf{N}} N \{\mathbf{a}^\intercal(\widehat{\boldsymbol{\theta}} - \boldsymbol{\theta}^*)\}^2 d \prod_{i=1}^N \mathbb{P}_{\mathbf{h}}(r_i, \mathbf{z}_i, \mathbf{w}_i) \tag{A.65}$$

where $d\mathbb{P}_{\mathbf{h}^\intercal \mathbf{g}}$ is the tilted distribution

$$\begin{aligned} & d\mathbb{P}_{\mathbf{h}}(r_i, \mathbf{z}_i, \mathbf{w}_i) \\ =& \rho_{\mathsf{N}} \left(f_* \left[1 + \frac{\mathbf{h}_1^\intercal \mathbf{g}_1}{\sqrt{\rho_{\mathsf{N}} N}} + \frac{\mathbf{h}_2^\intercal \mathbf{g}_2}{\sqrt{N}} \right]_+ \right) (\mathbf{z}_i, \mathbf{w}_i)/C_{\mathbf{h}} d\nu_{\mathsf{cmp}}(\mathbf{z}_i, \mathbf{w}_i) \times \delta_1(r_i) \\ &+ \int_{\mathbf{z}} (1 - \rho_{\mathsf{N}}) \left(f_* \left[1 + \frac{\mathbf{h}_1^\intercal \mathbf{g}_1}{\sqrt{\rho_{\mathsf{N}} N}} + \frac{\mathbf{h}_2^\intercal \mathbf{g}_2}{\sqrt{N}} \right]_+ \right) (\mathbf{z}_i, \mathbf{w}_i)/C_{\mathbf{h}} d\nu_{\mathsf{cmp}}(\mathbf{z}_i, \mathbf{w}_i) \times \delta_0(r_i). \end{aligned} \tag{A.66}$$

To simplify the notation, we invoke Assumption 5d and drop the truncation at zero for $\sqrt{\rho_{\mathsf{N}} N} > 2cM$,

$$\begin{aligned} & d\mathbb{P}_{\mathbf{h}}(r_i, \mathbf{z}_i, \mathbf{w}_i) \\ =& \rho_{\mathsf{N}} \left(f_* \left[1 + \frac{\mathbf{h}_1^\intercal \mathbf{g}_1}{\sqrt{\rho_{\mathsf{N}} N}} + \frac{\mathbf{h}_2^\intercal \mathbf{g}_2}{\sqrt{N}} \right] \right) (\mathbf{z}_i, \mathbf{w}_i) d\nu_{\mathsf{cmp}}(\mathbf{z}_i, \mathbf{w}_i) \times \delta_1(r_i) \\ &+ \int_{\mathbf{z}} (1 - \rho_{\mathsf{N}}) \left(f_* \left[1 + \frac{\mathbf{h}_1^\intercal \mathbf{g}_1}{\sqrt{\rho_{\mathsf{N}} N}} + \frac{\mathbf{h}_2^\intercal \mathbf{g}_2}{\sqrt{N}} \right] \right) (\mathbf{z}_i, \mathbf{w}_i) d\nu_{\mathsf{cmp}}(\mathbf{z}_i, \mathbf{w}_i) \times \delta_0(r_i). \end{aligned} \tag{A.67}$$

Notice that the tilted data distribution $d\mathbb{P}_{\mathbf{h}}$ has two components: 1) the model is restricted to the least favorable model; 2) the neighborhood along the direction of $\mathbf{g}_2$ is narrowed to $c/\sqrt{N}$. The representation would hold approximately with an error $2Mc/\sqrt{\rho_{\mathsf{N}} N}$ without Assumption 5d, following the approximation of total variation established in Lemma A25.

The design of our least favorable model leads to a factorization of the tilted model

$$
\begin{aligned}
&f_*(\mathbf{z},\mathbf{w})\left\{1+\frac{\mathbf{h}_1^\top\mathbf{g}_1(\mathbf{z},\mathbf{w})}{\sqrt{\rho_{\mathsf{N}}N}}+\frac{\mathbf{h}_2^\top\mathbf{g}_2(\mathbf{w})}{\sqrt{N}}\right\}\\
=&f^*_{\mathbf{Z}|\mathbf{W}}(\mathbf{z}\mid\mathbf{w})\left\{1+\frac{\mathbf{h}_1^\top\mathbf{g}_1(\mathbf{z},\mathbf{w})}{\sqrt{\rho_{\mathsf{N}}N}}\right\}f^*_{\mathbf{W}}(\mathbf{w})\left\{1+\frac{\mathbf{h}_2^\top\mathbf{g}_2(\mathbf{w})}{\sqrt{N}}\right\}+O\left(1/(N\sqrt{\rho_{\mathsf{N}}})\right),\\
&f^*_{\mathbf{W}}(\mathbf{w})=\int_{\mathbf{z}\in\mathcal{Z}}f_*(\mathbf{z},\mathbf{w})d\nu_z(\mathbf{z}),\ f^*_{\mathbf{Z}|\mathbf{W}}(\mathbf{z}\mid\mathbf{w})=f_*(\mathbf{z},\mathbf{w})/f^*_{\mathbf{W}}(\mathbf{w}).
\end{aligned}
$$

The factorization is also reflected in the decomposition of the log-likelihood ratio. By the definition of $\mathbf{g}_1$ and $\mathbf{g}_2$, we have the identities

$$
\begin{aligned}
\mathbb{E}_*\{\mathbf{g}_1(\mathbf{Z},\mathbf{W})\mid\mathbf{W}\}&=\mathbb{E}_*[\boldsymbol{\psi}_{\mathsf{cmp}}(\mathbf{Z},\mathbf{W})-\mathbb{E}_*\{\boldsymbol{\psi}_{\mathsf{cmp}}(\mathbf{Z},\mathbf{W})\mid\mathbf{W}\}\mid\mathbf{W}]=\mathbf{0},\\
\mathbb{E}_*\{\mathbf{g}_1(\mathbf{Z},\mathbf{W})\mathbf{g}_2(\mathbf{W})\}&=\mathbb{O}_{q\times q}.
\end{aligned}
\tag{A.68}
$$

For sufficiently large n and N, we have

$$
\begin{aligned}
&\log\left(\prod_{i=1}^N\frac{d\mathbb{P}_{\mathbf{h}}(R_i,\mathbf{Z}_i,\mathbf{W}_i)}{d\mathbb{P}_0(R_i,\mathbf{Z}_i,\mathbf{W}_i)}\right)\\
=&\sum_{i=1}^N R_i\log\left(1+\frac{\mathbf{h}_1^\top\mathbf{g}_1(\mathbf{Z}_i,\mathbf{W}_i)}{\sqrt{\rho_{\mathsf{N}}N}}+\frac{\mathbf{h}_2^\top\mathbf{g}_2(\mathbf{W}_i)}{\sqrt{N}}\right)\\
&+\sum_{i=1}^N(1-R_i)\log\left(1+\frac{\mathbf{h}_2^\top\mathbf{g}_2(\mathbf{w})}{\sqrt{N}}\right)\\
&+\sum_{i=1}^N(1-R_i)\log\left(\int\left\{1+\frac{\mathbf{h}_1^\top\mathbf{g}_1(\mathbf{z},\mathbf{w})}{\sqrt{\rho_{\mathsf{N}}N}}\right\}f_*(\mathbf{z},\mathbf{W}_i)d\nu_z(\mathbf{z})\right)\\
&-\sum_{i=1}^N(1-R_i)\log\left(\int f_*(\mathbf{z},\mathbf{W}_i)d\mu(\mathbf{z})\right)\\
=&(\rho_{\mathsf{N}}N)^{-1/2}\sum_{i=1}^N R_i\mathbf{h}_1^\top\mathbf{g}_1(\mathbf{Z}_i,\mathbf{W}_i)+N^{-1/2}\sum_{i=1}^N\mathbf{h}_2^\top\mathbf{g}_2(\mathbf{W}_i)\\
&+\frac{1}{2}\mathbf{h}_1^\top\mathcal{V}_{\psi,1}\mathbf{h}_1+\frac{1}{2}\mathbf{h}_2^\top\mathcal{V}_{\psi,2}\mathbf{h}_2+o_p(1),
\end{aligned}
\tag{A.69}
$$

where $\mathcal{V}_{\psi,1}$ and $\mathcal{V}_{\psi,2}$ were variances of $\mathbf{g}_1$ and $\mathbf{g}_2$ defined in (A.63). This shows that the locally asymptotically normality of the least favorable model.

Based on the local asymptotic normality of the proposed two-dimensional least favorable model (A.69) , we apply the standard “Le Cam” method (Le Cam and Yang, 2000; Tsybakov, 2009) of the minimax efficiency lower bound for parametric sub-model. First, we relax the supremum over the local neighborhood by the Bayesian posterior average over the neighborhood according to the truncated Gaussian prior,

$$
(\mathbf{h}_1^\top,\mathbf{h}_2^\top)^\top\sim p(\mathbf{h},c,\mathbb{A})=\frac{\phi(\mathbf{h},\mathbf{0},\mathbb{A})I(\|\mathbf{h}\|_2\le c)}{\int_{\|\mathbf{h}\|_2\le c}\phi(\mathbf{h},\mathbf{0},\mathbb{A})d\mathbf{h}},\ \phi(\mathbf{v},\boldsymbol{\mu},\Sigma)=\frac{\exp\left(-(\mathbf{v}-\boldsymbol{\mu})^\top\Sigma^{-1}(\mathbf{v}-\boldsymbol{\mu})/2\right)}{(2\pi)^{-q}\det(\Sigma)^{-1/2}},
$$

$$\begin{aligned}&\liminf_{c\to\infty}\liminf_{N\to\infty}\sup_{\|\mathbf{h}_1\|_2^2+\|\mathbf{h}_2\|_2^2\le c^2}\int n\{\mathbf{a}^{\intercal}(\widehat{\boldsymbol\theta}-\boldsymbol\theta_{\mathbf{h}})\}^2 d\prod_{i=1}^{N}\mathbb{P}_{\mathbf{h}}(r_i,\mathbf{z}_i,\mathbf{w}_i)\\ \ge&\liminf_{c\to\infty}\liminf_{N\to\infty}\int\int n\{\mathbf{a}^{\intercal}(\widehat{\boldsymbol\theta}-\boldsymbol\theta_{\mathbf{h}})\}^2 d\prod_{i=1}^{N}\mathbb{P}_{\mathbf{h}}(r_i,\mathbf{z}_i,\mathbf{w}_i)\times p(\mathbf{h},c,\mathbb{A})d\mathbf{h}.\end{aligned}$$

Following Chapter 6 of Le Cam and Yang (2000), we concluded in Lemma A26 that the posterior distribution $\mathbf{h}\mid\mathscr{D}_N$ with $\mathscr{D}_N=\{(R_i,R_i\mathbf{Z}_i,\mathbf{W}_i):i=1,\dots,N\}$ approaches the Gaussian posterior with (untruncated) Gaussian prior $\phi(\mathbf{h},\mathbf{0},\mathbb{A})$ and Gaussian data $\mathbf{V}\mid\mathbf{h}\to N(\mathbf{h},\mathcal{V}_\psi)$ whose variance $\mathcal{V}_\psi$ is defined in (A.63). The limiting Gaussian data $\mathbf{V}$ comes from the limits of empirical processes in the log likelihood of the LAN model $\mathbb{P}_{\mathbf{h}}$,

$$\mathbf{V}=(\mathbf{V}_1^{\intercal},\mathbf{V}_2^{\intercal})^{\intercal},\ \mathbf{V}_1=-\sum_{i=1}^{N}R_i\mathbf{g}_1(\mathbf{Z}_i,\mathbf{W}_i)/\sqrt{\rho_{\mathsf{N}}N},\ \mathbf{V}_2=-\sum_{i=1}^{N}\mathbf{g}_2(\mathbf{W}_i)/\sqrt{N}.$$

The limiting Gaussian posterior of $\mathbf{h}=(\mathbf{h}_1^{\intercal},\mathbf{h}_2^{\intercal})^{\intercal}$ is thus

$$\mathbf{h}\mid\mathscr{D}_N\xrightarrow{TV}\widetilde{\mathbf{h}}\mid\mathbf{V}\sim N\left(\widetilde{\boldsymbol\mu},\widetilde{\mathcal{V}}\right),\widetilde{\mathcal{V}}=(\mathbb{A}+\mathcal{V}_\psi)^{-1},\widetilde{\boldsymbol\mu}=\widetilde{\mathcal{V}}\mathcal{V}_\psi\mathbf{V}. \tag{A.70}$$

According to Lemma A26, the average aMSE over truncated Gaussian prior approaches the average aMSE over Gaussian posterior and marginal,

$$\begin{aligned}&\liminf_{c\to\infty}\liminf_{N\to\infty}\int\int\rho_{\mathsf{N}}N\{\mathbf{a}^{\intercal}(\widehat{\boldsymbol\theta}-\boldsymbol\theta_{\mathbf{h}})\}^2 d\prod_{i=1}^{N}\mathbb{P}_{\mathbf{h}}(r_i,\mathbf{z}_i,\mathbf{w}_i)p(\mathbf{h},c,\mathbb{A})d\mathbf{h}\\ =&\liminf_{c\to\infty}\liminf_{N\to\infty}\int\int\rho_{\mathsf{N}}N\{\mathbf{a}^{\intercal}(\widehat{\boldsymbol\theta}-\boldsymbol\theta_{\widetilde{\mathbf{h}}})\}^2\phi(\widetilde{\mathbf{h}},\widetilde{\boldsymbol\mu},\widetilde{\mathcal{V}})d\widetilde{\mathbf{h}}\times\phi(\mathbf{v},\mathbf{0},\mathcal{V}_\psi+\mathbb{A})d\mathbf{v}\\ =&\liminf_{c\to\infty}\liminf_{N\to\infty}\widetilde{\mathbb{E}}\left(\widetilde{\mathbb{E}}\left[\rho_{\mathsf{N}}N\{\mathbf{a}^{\intercal}(\widehat{\boldsymbol\theta}-\boldsymbol\theta_{\widetilde{\mathbf{h}}})\}^2\mid\mathbf{V}\right]\right).\end{aligned} \tag{A.71}$$

The expectation $\widetilde{\mathbb{E}}$ is taken according to the limiting Gaussian models $\mathbf{V}\sim N(\mathbf{0},\mathcal{V}_\psi+\mathbb{A})$ and $\widetilde{\mathbf{h}}\mid\mathbf{V}\sim N(\widetilde{\mu},\widetilde{\mathcal{V}})$ as defined in (A.70). We are using expression in (A.71) as the ultimate characterization of the asymptotic mean squared estimation error initially defined in (A.65).

While the efficient influence function $\boldsymbol\psi_{\mathsf{cmp}}$ under complete data setting $\mathcal{S}_{\mathsf{cmp}}$ can be characterized in multiple ways, we specifically chose the following definition connecting to the least-favorable model (van der Vaart, 1998, Section 25.3).

Definition A18 *Under local exponential-tilt sub-model,*

$$f_{\mathbf{h}}(\mathbf{z},\mathbf{w})=f_*(\mathbf{z},\mathbf{w})[1+\mathbf{h}^{\intercal}\mathbf{g}(\mathbf{z},\mathbf{w})]_+/C_{\mathbf{h}},\ C_{\mathbf{h}}=\mathbb{E}_*\left([1+\mathbf{h}^{\intercal}\mathbf{g}(\mathbf{z},\mathbf{w})]_+\right),$$

the local shift of parameter $\boldsymbol\theta$ along $\mathbf{h}$ observe

$$\boldsymbol\theta_{\mathbf{h}}=\boldsymbol\theta_*+\mathbb{E}_*\{\boldsymbol\psi_{\mathsf{cmp}}(\mathbf{Z},\mathbf{W})\mathbf{g}(\mathbf{Z},\mathbf{W})^{\intercal}\}\mathbf{h}+o(\|\mathbf{h}\|_2).$$

According to Definition A18, estimating $\boldsymbol{\theta}$ under our chosen local sub-model (A.61) is asymptotically equivalent to projecting the estimated local sub-model

$$\begin{aligned}
\boldsymbol{\theta}_{\mathbf{h}} =& \boldsymbol{\theta}_* + \mathbb{E}_*\{\boldsymbol{\psi}_{\mathsf{cmp}}(\mathbf{Z},\mathbf{W})\mathbf{g}_1(\mathbf{Z},\mathbf{W})^{\mathsf{T}}\}\frac{\mathbf{h}_1}{\sqrt{\rho_{\mathsf{N}}N}} + \mathbb{E}_*\{\boldsymbol{\psi}_{\mathsf{cmp}}(\mathbf{Z},\mathbf{W})\mathbf{g}_2(\mathbf{Z},\mathbf{W})^{\mathsf{T}}\}\frac{\mathbf{h}_2}{\sqrt{N}} + o\left(\frac{c}{\sqrt{\rho_{\mathsf{N}}N}}\right) \\
=& \boldsymbol{\theta}_* + \mathcal{V}_{\psi,1}\frac{\mathbf{h}_1}{\sqrt{\rho_{\mathsf{N}}N}} + \mathcal{V}_{\psi,2}\frac{\mathbf{h}_2}{\sqrt{N}} + o\left(\frac{c}{\sqrt{\rho_{\mathsf{N}}N}}\right), \\
=& \boldsymbol{\theta}_* + \frac{1}{\sqrt{\rho_{\mathsf{N}}N}}\left\{\mathcal{V}_{\psi,1}\mathbf{h}_1 + \sqrt{\rho_{\mathsf{N}}}\mathcal{V}_{\psi,2}\mathbf{h}_2 + o\left(c\right)\right\}, \\
\widehat{\boldsymbol{\theta}} =& \boldsymbol{\theta}_* + \frac{1}{\sqrt{\rho_{\mathsf{N}}N}}\left\{\mathcal{V}_{\psi,1}\widehat{\mathbf{h}}_1 + \sqrt{\rho_{\mathsf{N}}}\mathcal{V}_{\psi,2}\widehat{\mathbf{h}}_2 + o\left(c\right)\right\}.
\end{aligned}$$

The variances $\mathcal{V}_{\psi,1}$ and $\mathcal{V}_{\psi,2}$ of $\mathbf{g}_1$ and $\mathbf{g}_2$ above have been defined in (A.63). The asymptotic mean squared estimation error of $\widehat{\boldsymbol{\theta}}$ can be derived from that of $\widehat{\mathbf{h}}$,

$$\begin{aligned}
&\widetilde{\mathbb{E}}\left[\rho_{\mathsf{N}}N\{\mathbf{a}^{\mathsf{T}}(\widehat{\boldsymbol{\theta}} - \boldsymbol{\theta}_{\mathbf{h}})\}^2 \mid \mathbf{V}\right] \\
=&\widetilde{\mathbb{E}}\left[\left\{\mathbf{a}^{\mathsf{T}}(\mathcal{V}_{\psi,1}, \sqrt{\rho_{\mathsf{N}}}\mathcal{V}_{\psi,2})(\widehat{\mathbf{h}} - \mathbf{h})\right\}^2 \mid \mathbf{V}\right] + o(c^2) \\
=&\mathbf{a}^{\mathsf{T}}(\mathcal{V}_{\psi,1}, \sqrt{\rho_{\mathsf{N}}}\mathcal{V}_{\psi,2})\widetilde{\mathbb{E}}\left\{(\widehat{\mathbf{h}} - \mathbf{h})(\widehat{\mathbf{h}} - \mathbf{h})^{\mathsf{T}} \mid \mathbf{V}\right\}(\mathcal{V}_{\psi,1}, \sqrt{\rho_{\mathsf{N}}}\mathcal{V}_{\psi,2})^{\mathsf{T}}\mathbf{a} + o(c^2). \qquad \text{(A.72)}
\end{aligned}$$

Conditioning on $\mathbf{V}_1, \mathbf{V}_2$, the asymptotically optimal $\widehat{\mathbf{h}}$ is given by the conditional mean $\widehat{\mathbf{h}} = \widetilde{\mathbb{E}}(\mathbf{h} \mid \mathbf{V}_1, \mathbf{V}_2)$ according to the Andersen's Lemma

$$\mathbf{u}^{\mathsf{T}}\widetilde{\mathbb{E}}\left\{(\widehat{\mathbf{h}} - \mathbf{h})(\widehat{\mathbf{h}} - \mathbf{h})^{\mathsf{T}} \mid \mathbf{V}\right\}\mathbf{u} = \widetilde{\mathbb{E}}\left[\{\mathbf{u}^{\mathsf{T}}(\widehat{\mathbf{h}} - \mathbf{h})\}^2 \mid \mathbf{V}\right] \geq \mathbf{u}^{\mathsf{T}}\widetilde{\mathcal{V}}\mathbf{u}, \qquad \text{(A.73)}$$

where $\widetilde{\mathcal{V}}$ is the posterior variance of $\mathbf{h} \mid \mathbf{V}$ defined in (A.70). Applying (A.73) and (A.72) to the characterization of asymptotic mean squared estimation error (A.71), we have established the lower bound

$$\begin{aligned}
aMSE \geq& \liminf_{c\to\infty}\liminf_{N\to\infty}\widetilde{\mathbb{E}}\left\{\mathbf{a}^{\mathsf{T}}(\mathcal{V}_{\psi,1}, \sqrt{\rho_{\mathsf{N}}}\mathcal{V}_{\psi,2})\widetilde{\mathcal{V}}(\mathcal{V}_{\psi,1}, \sqrt{\rho_{\mathsf{N}}}\mathcal{V}_{\psi,2})^{\mathsf{T}}\mathbf{a} + o(c^2)\right\} \\
\geq& \mathbf{a}^{\mathsf{T}}(\mathcal{V}_{\psi,1}, \sqrt{\rho_{\mathsf{N}}}\mathcal{V}_{\psi,2})\left\{\mathbb{A} + \mathcal{V}_{\psi}\right\}^{-1}\begin{pmatrix}\mathcal{V}_{\psi,1} \\ \sqrt{\rho_{\mathsf{N}}}\mathcal{V}_{\psi,2}\end{pmatrix}\mathbf{a}. \qquad \text{(A.74)}
\end{aligned}$$

The lower bound (A.74) holds for any prior of $\mathbf{h}$ with arbitrary positive definite $\mathbb{A}$, so the limit of lower bound when $\mathbb{A} \to \mathbb{O}$ is still a lower bound,

$$\begin{aligned}
aMSE \geq& \liminf_{\|\mathbb{A}\|_2\to 0}\mathbf{a}^{\mathsf{T}}(\mathcal{V}_{\psi,1}, \sqrt{\rho_{\mathsf{N}}}\mathcal{V}_{\psi,2})\left\{\mathbb{A} + \mathcal{V}_{\psi}\right\}^{-1}\begin{pmatrix}\mathcal{V}_{\psi,1} \\ \sqrt{\rho_{\mathsf{N}}}\mathcal{V}_{\psi}\end{pmatrix}\mathbf{a} \\
\geq& \mathbf{a}^{\mathsf{T}}(\mathcal{V}_{\psi,1}, \sqrt{\rho_{\mathsf{N}}}\mathcal{V}_{\psi,2})\begin{pmatrix}\mathcal{V}_{\psi,1}^{-1}, & \mathbb{O} \\ \mathbb{O}, & \mathcal{V}_{\psi,2}^{-1}\end{pmatrix}\begin{pmatrix}\mathcal{V}_{\psi,1} \\ \sqrt{\rho_{\mathsf{N}}}\mathcal{V}_{\psi,2}\end{pmatrix}\mathbf{a} \\
=& \mathbf{a}^{\mathsf{T}}(\mathcal{V}_{\psi,1} + \rho_{\mathsf{N}}\mathcal{V}_{\psi,2})\mathbf{a}.
\end{aligned}$$

The lower bound is proportional to the variance of proposed SMMAL influence function

$$\boldsymbol{\psi}_{\mathsf{SSL}}(R, R\mathbf{Z}, \mathbf{W}) = R\mathbf{g}_1(\mathbf{Z}, \mathbf{W})/\rho_N + \mathbf{g}_2(\mathbf{W}),\ \mathrm{Var}\{\sqrt{\rho_{\mathsf{N}}}\boldsymbol{\psi}_{\mathsf{SSL}}(R, R\mathbf{Z}, \mathbf{W})\} = \mathcal{V}_{\psi,1} + \rho_{\mathsf{N}}\mathcal{V}_{\psi,2}.$$

The Assumption 5b is only needed in the end to show that the variance $\rho_{\mathsf{N}}\mathrm{Var}_*\{\boldsymbol{\psi}_{\mathsf{SSL}}(R, \mathbf{Z}, \mathbf{W})\}$ is not degenerating when $\rho_{\mathsf{N}} \to 0$.

Appendix D. Auxiliary Lemmas

Lemma A19 *Let $h_1(Y, A, \mathbf{W})$ and $h_2(Y, A, \mathbf{W})$ be two uniformly bounded measurable functions. We have the concentration*

$$\frac{1}{N}\sum_{i=1}^{N} h_1(Y_i, A_i, \mathbf{W}_i) + \frac{R_i}{\rho_{\mathsf{N}}} h_2(Y_i, A_i, \mathbf{W}_i) - \mathbb{E}\{h_1(Y, A, \mathbf{W}) + h_2(Y, A, \mathbf{W})\} = O_p\left(n^{-1/2}\right).$$

Proof [Proof of Lemma A19]

First establish the rate for $\frac{\sqrt{\rho_{\mathsf{N}}}}{N}\sum_{i=1}^{N}(R_i/\rho_{\mathsf{N}} - 1)$. By the variance expression

$$\mathrm{Var}\{\sqrt{\rho_{\mathsf{N}}}(R_i/\rho_{\mathsf{N}} - 1)\} = 1 - \rho_{\mathsf{N}},$$

we may apply the Tchebychev's inequality to obtain

$$\frac{\sqrt{\rho_{\mathsf{N}}}}{N}\sum_{i=1}^{N}(R_i/\rho_{\mathsf{N}} - 1) = O_p\left(N^{-1/2}\right).$$

Thus, we have two consequences

$$\begin{aligned}
\frac{\sum_{i=1}^{N} R_i}{\rho_{\mathsf{N}} N} - 1 &= O_p\left((\rho_{\mathsf{N}} N)^{-1/2}\right) = O_p\left(n^{-1/2}\right), \\
\left(\sum_{i=1}^{N} R_i\right)^{-1/2} &= \left\{n + O_p\left(\sqrt{n}\right)\right\}^{-1/2} = O_p\left(n^{-1/2}\right). \qquad \text{(A.75)}
\end{aligned}$$

Now, we decompose the empirical process of interest

$$\begin{aligned}
&\frac{1}{N}\sum_{i=1}^{N} h_1(Y_i, A_i, \mathbf{W}_i) + \frac{R_i}{\rho_{\mathsf{N}}} h_2(Y_i, A_i, \mathbf{W}_i) - \mathbb{E}\{h_1(Y, A, \mathbf{W}) + h_2(Y, A, \mathbf{W})\} \\
=&\frac{1}{N}\sum_{i=1}^{N}[h_1(Y_i, A_i, \mathbf{W}_i) - \mathbb{E}\{h_1(Y, A, \mathbf{W})\}] \\
&+ \frac{\sum_{i:R_i=1} h_2(Y_i, A_i, \mathbf{W}_i) - \mathbb{E}\{h_2(Y, A, \mathbf{W})\}}{\sum_{i=1}^{N} R_i} \frac{\sum_{i=1}^{N} R_i}{\rho_{\mathsf{N}} N}. \qquad \text{(A.76)}
\end{aligned}$$

Conditionally on $R_1, \ldots, R_N$, we apply the Hoeffding's inequality,

$$\begin{aligned}
\frac{1}{N}\sum_{i=1}^{N}[h_1(Y_i, A_i, \mathbf{W}_i) - \mathbb{E}\{h_1(Y, A, \mathbf{W})\}] &= O_p\left(N^{-1/2}\right), \\
\frac{\sum_{i:R_i=1} h_2(Y_i, A_i, \mathbf{W}_i) - \mathbb{E}\{h_2(Y, A, \mathbf{W})\}}{\sum_{i=1}^{N} R_i} &= O_p\left(\left(\sum_{i=1}^{N} R_i\right)^{-1/2}\right). \qquad \text{(A.77)}
\end{aligned}$$

Applying the rates of (A.75) and (A.77) to (A.76), we have shown

$$\frac{1}{N}\sum_{i=1}^{N} h_1(Y_i, A_i, \mathbf{W}_i) + \frac{R_i}{\rho_{\mathsf{N}}} h_2(Y_i, A_i, \mathbf{W}_i) - \mathbb{E}\{h_1(Y, A, \mathbf{W}) + h_2(Y, A, \mathbf{W})\}$$

$$
\begin{aligned}
&= O_p\left(N^{-1/2}\right) + O_p\left(\left(\sum_{i=1}^{N} R_i\right)^{-1/2}\right)\left\{1 + O_p\left(n^{-1/2}\right)\right\} \\
&= O_p\left(n^{-1/2}\right).
\end{aligned}
$$

■

Lemma A20 *Let* $\mathbf{X}$ *be sub-Gaussian random vector satisfying* $\sup_{\|\mathbf{v}\|_2=1} \|\mathbf{v}^\intercal \mathbf{X}\|_{\psi_2} \leq M$ *,* $g(x)$ *be a continuously differentiable link function and* $\tau(x) = \text{sign}(x)\min\{2M, |x|\}$ *be a truncation at* $2M$*. For coefficient* $\bar{\boldsymbol{\beta}}$ *satisfying* $|\mathbf{X}^\intercal \bar{\boldsymbol{\beta}}| \leq M$ *almost surely, we have the mean squared error bound*

$$
\sqrt{\mathbb{E}[\{g(\mathbf{X}^\intercal \bar{\boldsymbol{\beta}}) - g(\mathbf{X}^\intercal \boldsymbol{\beta})\}^2]} \leq \sqrt{2}/4M\|\bar{\boldsymbol{\beta}} - \boldsymbol{\beta}\|_2,
$$
$$
\sqrt{\mathbb{E}[\{g(\mathbf{X}^\intercal \bar{\boldsymbol{\beta}}) - g_\tau(\mathbf{X}^\intercal \boldsymbol{\beta})\}^2]} \leq \sqrt{2}/4M\|\bar{\boldsymbol{\beta}} - \boldsymbol{\beta}\|_2.
$$

Proof [Proof of Lemma A20] We focus on the case with truncation. The case without truncation can be derived from the same steps. By the Mean Value Theorem, we have

$$
g(\mathbf{X}^\intercal \bar{\boldsymbol{\beta}}) - g_\tau(\mathbf{X}^\intercal \boldsymbol{\beta}) = \dot{g}(t)\{\mathbf{X}^\intercal \bar{\boldsymbol{\beta}} - \tau(\mathbf{X}^\intercal \boldsymbol{\beta})\}
$$

for some t between $\mathbf{X}^\intercal \bar{\boldsymbol{\beta}}$ and $\tau(\mathbf{X}^\intercal \boldsymbol{\beta})$. The link function for logistic regression has bounded derivative $|\dot{g}(t)| \leq 1/4$. The truncation at $2M$ never increases estimation error

$$
|\mathbf{X}^\intercal \bar{\boldsymbol{\beta}} - \tau(\mathbf{X}^\intercal \boldsymbol{\beta})| \begin{cases} = |\mathbf{X}^\intercal \bar{\boldsymbol{\beta}} - \mathbf{X}^\intercal \boldsymbol{\beta}|, & |\mathbf{X}^\intercal \boldsymbol{\beta}| \leq 2M \\ < |\mathbf{X}^\intercal \bar{\boldsymbol{\beta}} - \mathbf{X}^\intercal \boldsymbol{\beta}|, & |\mathbf{X}^\intercal \boldsymbol{\beta}| > 2M \end{cases}.
$$

Thus, we have

$$
\sqrt{\mathbb{E}[\{g(\mathbf{X}^\intercal \bar{\boldsymbol{\beta}}) - g_\tau(\mathbf{X}^\intercal \boldsymbol{\beta})\}^2]} \leq 1/4\sqrt{\mathbb{E}[\{\mathbf{X}^\intercal(\bar{\boldsymbol{\beta}} - \boldsymbol{\beta})\}^2]}.
$$

Applying the sub-Gaussian property of $\mathbf{X}$, we have

$$
\|\mathbf{X}^\intercal(\bar{\boldsymbol{\beta}} - \boldsymbol{\beta})\|_{\psi_2} \leq M\|\bar{\boldsymbol{\beta}} - \boldsymbol{\beta}\|_2.
$$

Using the bound of moments for sub-Gaussian random variables, we have

$$
\mathbb{E}[\{\mathbf{X}^\intercal(\bar{\boldsymbol{\beta}} - \boldsymbol{\beta})\}^2] \leq 2M^2\|\bar{\boldsymbol{\beta}} - \boldsymbol{\beta}\|_2^2.
$$

Putting everything together, we have the conclusion

$$
\sqrt{\mathbb{E}[\{g(\mathbf{X}^\intercal \bar{\boldsymbol{\beta}}) - g_\tau(\mathbf{X}^\intercal \boldsymbol{\beta})\}^2]} \leq \sqrt{2}/4M\|\bar{\boldsymbol{\beta}} - \boldsymbol{\beta}\|_2.
$$

■

D1 Lasso with Cross-fitted Parameters

We establish the estimation rates for a generic problems. There are two estimation rates in Lemma A21. In the general case, the asymptotic solution can not be identified by the population level first order condition due to a bias from the cross-fitted parameters. The general case applies to the Lasso of a mis-specified model with cross-fitted parameters. In the special case, the asymptotic solution can be identified by the population level first order condition. The special case applies to the Lasso with no cross-fitted parameters or the Lasso of a correctly specified model which might include cross-fitted parameters.

Consider the cross-fitted Lasso estimator with folds $\{1,\ldots,n\} = \sqcup_{k=1}^{K}\mathcal{I}_k$,

$$\widehat{\boldsymbol{\beta}} = \operatorname*{argmin}_{\boldsymbol{\beta}\in\mathbb{R}^p} \frac{1}{K}\sum_{k=1}^{K} \ell_k(\boldsymbol{\beta};\widehat{\boldsymbol{\gamma}}^{(\mathrm{k})}) + \lambda\|\boldsymbol{\beta}\|_1 \tag{A.78}$$

whose loss has derivatives with respect to $\boldsymbol{\beta}$ of the following forms:

$$\dot{\boldsymbol{\ell}}_k(\boldsymbol{\beta};\boldsymbol{\gamma}) = \frac{K}{n}\sum_{i\in\mathcal{I}_k} w_1(\boldsymbol{\gamma}^\top\mathbf{X}_i)w_2(\boldsymbol{\beta}^\top\mathbf{X}_i)\mathbf{X}_i\{Y_i - g(\boldsymbol{\beta}^\top\mathbf{X}_i)\},$$
$$\ddot{\mathbb{I}}_k(\boldsymbol{\beta};\boldsymbol{\gamma}) = \frac{K}{n}\sum_{i\in\mathcal{I}_k} w_1(\boldsymbol{\gamma}^\top\mathbf{X}_i)w_3(\boldsymbol{\beta}^\top\mathbf{X}_i)\mathbf{X}_i\mathbf{X}_i^\top \tag{A.79}$$

for nonnegative weights w_1, w_2 and w_3. The solution is identified by the population minimum at a specific $\bar{\boldsymbol{\gamma}}$,

$$\bar{\boldsymbol{\beta}} = \operatorname*{argmin}_{\boldsymbol{\beta}\in\mathbb{R}^p} \mathbb{E}\{\ell_k(\boldsymbol{\beta};\bar{\boldsymbol{\gamma}})\},\ \|\bar{\boldsymbol{\beta}}\|_0 = s. \tag{A.80}$$

We make the following generic assumptions:

Assumption 6 *(a) (Sub-Gaussian and bounded Covariates)* $\sup_{\|\mathbf{v}\|_2=1}\|\mathbf{v}^\top\mathbf{X}\|_{\psi_2} \le M$ *and* $\|\mathbf{X}\|_\infty \le M$ *almost surely;*

(b) (Bounded responses) $|Y| \le M$ *almost surely and* $\|g\|_\infty \le M$*;*

(c) (Identifiability) $\inf_{\|\mathbf{v}\|_2=1}\mathbf{v}^\top\mathbb{E}(\mathbf{X}\mathbf{X}^\top)\mathbf{v} \ge 1/M$*;*

(d) (Bounds for weights) $w_1(x)\in[1/M,M]\ \forall x\in\mathbb{R}$, $\|w_1'\|_\infty \le M$, $w_2(\bar{\boldsymbol{\beta}}^\top\mathbf{X})\in[1/M,M]$ *and* $w_3(\bar{\boldsymbol{\beta}}^\top\mathbf{X})\in[1/M,M]$ *almost surely;*

(e) (Restricted strong convexity) w_3 *is the derivative of some generalized linear model link satisfying* $\|w_3\|_\infty \le M$ *or* $\mathbb{E}\left[\sup_{|u|<1}\{w_3(\bar{\boldsymbol{\beta}}^\top\mathbf{X}+u)\}^\alpha\right] \le M$ *for some* $\alpha \ge 2$.

Assumption 6 covers all the estimators in (9)-(12) under Assumption 4. The truncations in (12) secure the requirement for w_1 in Assumption 6d. Two w_3 needed for (9)-(12) correspond to the link of logistic regression $g(x)$ and Poisson model e^x, both have been studied in Negahban et al. (2010).

Lemma A21 *Choose the penalty* $\lambda \asymp \sqrt{\log(p)/n}$ *such that*

$$\lambda \ge \frac{3}{K}\sum_{k=1}^{K}\left\|\dot{\boldsymbol{\ell}}_k(\bar{\boldsymbol{\beta}};\widehat{\boldsymbol{\gamma}}^{(\mathrm{k})}) - \mathbb{E}_{i\in\mathcal{I}_k}\{\dot{\boldsymbol{\ell}}_k(\bar{\boldsymbol{\beta}};\widehat{\boldsymbol{\gamma}}^{(\mathrm{k})}) \mid \mathscr{D}_k\}\right\|_\infty + 3\kappa_1\kappa_2/M\sqrt{\log(p)/n} \tag{A.81}$$

with large probability for a restricted strong convexity (Negahban et al., 2012) constants κ_1 *and* κ_2 *associated with the auxiliary loss*

$$\widetilde{\ell}_k(\boldsymbol{\beta}) = \frac{K}{n} \sum_{i \in \mathcal{I}_k} \widetilde{G}(\boldsymbol{\beta}^\intercal \mathbf{X}_i) - \widetilde{Y}_i \boldsymbol{\beta}^\intercal \mathbf{X}_i,\ \widetilde{G}'' = w_3.$$

Under Assumption 6, we have

$$\textit{In general: } \|\widehat{\boldsymbol{\beta}} - \bar{\boldsymbol{\beta}}\|_2 = O_p\left(\sqrt{s\log(p)/n}\right) + \sup_{k=1,\ldots,K} \|\widehat{\boldsymbol{\gamma}}^{(\mathsf{k})} - \bar{\boldsymbol{\gamma}}\|_2,$$

$$\textit{Special case: } \mathbb{E}_{i \in \mathcal{I}_k}\{\dot{\boldsymbol{\ell}}_k(\bar{\boldsymbol{\beta}}; \widehat{\boldsymbol{\gamma}}^{(\mathsf{k})}) \mid \mathscr{D}_k\} = \mathbf{0},\ k = 1, \ldots, K,\ \|\widehat{\boldsymbol{\beta}} - \bar{\boldsymbol{\beta}}\|_2 = O_p\left(\sqrt{s\log(p)/n}\right).$$

Proof [Proof of Lemma A21] We focus on the proof of the "in general" case. The proof of the "special case" is simpler and can be made by dropping the steps regarding $\mathbb{E}_{i \in \mathcal{I}_k}\{\dot{\boldsymbol{\ell}}^{(\mathsf{k})}(\bar{\boldsymbol{\beta}}; \widehat{\boldsymbol{\gamma}}^{(\mathsf{k})}) \mid \mathscr{D}_k\}$ from proof of the "in general" case. The proof of the "in general" case takes three steps. First, we justify that the oracle choice yields $\lambda \asymp \sqrt{\log(p)/n}$. Second, we obtain a preliminary bound for the estimation error $\|\widehat{\boldsymbol{\beta}} - \bar{\boldsymbol{\beta}}\|_2$ through the restricted strong convexity argument. Third and finally, we analyze the preliminary bound in two situations: 1) the error inherited from $\widehat{\boldsymbol{\gamma}}^{(\mathsf{k})}$ is dominant, which leads to an immediate bound for $\|\widehat{\boldsymbol{\gamma}}^{(\mathsf{k})} - \bar{\boldsymbol{\gamma}}\|_2$; 2) the error from Lasso is dominant, which leads to the typical cone property analysis for Lasso.

We first validate the rate for oracle λ. Under Assumptions 6a, 6b and 6d, the summands in $\dot{\boldsymbol{\ell}}_k(\bar{\boldsymbol{\beta}}; \widehat{\boldsymbol{\gamma}}^{(\mathsf{k})})$ have bounded infinity norm

$$\|w_1(\boldsymbol{\gamma}^\intercal \mathbf{X}_i) w_2(\boldsymbol{\beta}^\intercal \mathbf{X}_i) \mathbf{X}_i \{Y_i - g(\boldsymbol{\beta}^\intercal \mathbf{X}_i)\}\|_\infty \leq 2M^4.$$

By the union bound of the element wise Hoeffding inequality, we have

$$\left\|\dot{\boldsymbol{\ell}}_k(\bar{\boldsymbol{\beta}}; \widehat{\boldsymbol{\gamma}}^{(\mathsf{k})}) - \mathbb{E}_{i \in \mathcal{I}_k}\{\dot{\boldsymbol{\ell}}_k(\bar{\boldsymbol{\beta}}; \widehat{\boldsymbol{\gamma}}^{(\mathsf{k})}) \mid \mathscr{D}_k\}\right\|_\infty = O_p\left(\sqrt{\log(p)/n}\right).$$

Thus, we may choose $\lambda \asymp \sqrt{\log(p)/n}$ to satisfy (A.81) with large probability.

By the definition of $\widehat{\boldsymbol{\beta}}$, we have

$$\frac{1}{K} \sum_{k=1}^K \ell_k(\widehat{\boldsymbol{\beta}}; \widehat{\boldsymbol{\gamma}}^{(\mathsf{k})}) + \lambda \|\widehat{\boldsymbol{\beta}}\|_1 \leq \frac{1}{K} \sum_{k=1}^K \ell_k(\bar{\boldsymbol{\beta}}; \widehat{\boldsymbol{\gamma}}^{(\mathsf{k})}) + \lambda \|\bar{\boldsymbol{\beta}}\|_1. \tag{A.82}$$

Denote the standardized estimation error as $\boldsymbol{\delta} = (\widehat{\boldsymbol{\beta}} - \bar{\boldsymbol{\beta}})/\|\widehat{\boldsymbol{\beta}} - \bar{\boldsymbol{\beta}}\|_2$. The Hessian of the loss in (A.79) is positive semi-definite under Assumptions 6d,

$$\mathbf{v}^\intercal \frac{1}{K} \sum_{k=1}^K \ddot{\mathbb{l}}_k(\boldsymbol{\beta}; \widehat{\boldsymbol{\gamma}}^{(\mathsf{k})}) \mathbf{v} = \frac{1}{K} \sum_{k=1}^K w_1(\boldsymbol{\gamma}^\intercal \mathbf{X}_i) w_3(\boldsymbol{\beta}^\intercal \mathbf{X}_i) (\mathbf{v}^\intercal \mathbf{X}_i)^2 \geq 0, \tag{A.83}$$

indicating that the loss is convex. Using the convexity of the loss function, we have for the truncated L_2-estimation error $t = \min\{\|\widehat{\boldsymbol{\beta}} - \bar{\boldsymbol{\beta}}\|_2, 1\}$

$$\frac{1}{K} \sum_{k=1}^K \ell_k(\bar{\boldsymbol{\beta}} + t\boldsymbol{\delta}; \widehat{\boldsymbol{\gamma}}^{(\mathsf{k})}) + \lambda \|\bar{\boldsymbol{\beta}} + t\boldsymbol{\delta}\|_1 \leq \frac{1}{K} \sum_{k=1}^K \ell_k(\bar{\boldsymbol{\gamma}}; \widehat{\boldsymbol{\gamma}}^{(\mathsf{k})}) + \lambda \|\bar{\boldsymbol{\beta}}\|_1. \tag{A.84}$$

By the triangle inequality $\|\bar{\boldsymbol{\beta}}\|_1 - \|\bar{\boldsymbol{\beta}} + t\boldsymbol{\delta}\|_1 \leq t\|\boldsymbol{\delta}\|_1$, we have from (A.84)

$$\frac{1}{K}\sum_{k=1}^{K} \ell_k(\bar{\boldsymbol{\beta}} + t\boldsymbol{\delta}; \widehat{\boldsymbol{\gamma}}^{(\mathrm{k})}) - \ell_k(\bar{\boldsymbol{\beta}}; \widehat{\boldsymbol{\gamma}}^{(\mathrm{k})}) \leq t\lambda_\gamma \|\boldsymbol{\delta}\|_1 \tag{A.85}$$

Now, we establish the restricted strong convexity property for each $\ell_k(\cdot; \widehat{\boldsymbol{\gamma}}^{(\mathrm{k})})$. By Assumption 6e, we may a hypothetical generalized linear model loss for $\widetilde{Y}_i \sim \widetilde{G}'(\bar{\boldsymbol{\beta}}^\intercal \mathbf{X}_i)$

$$\widetilde{\ell}_k(\boldsymbol{\beta}) = \frac{K}{n}\sum_{i\in\mathcal{I}_k} \widetilde{G}(\boldsymbol{\beta}^\intercal \mathbf{X}_i) - \widetilde{Y}_i \boldsymbol{\beta}^\intercal \mathbf{X}_i,\ \widetilde{G}'' = w_3. \tag{A.86}$$

The restricted strong convexity of (A.86) is established by analyzing the lower bound for

$$\widetilde{\ell}_k(\bar{\boldsymbol{\beta}} + \boldsymbol{\Delta}) - \widetilde{\ell}_k(\bar{\boldsymbol{\beta}}) - \boldsymbol{\Delta}^\intercal \dot{\widetilde{\boldsymbol{\ell}}}(\bar{\boldsymbol{\beta}}) = \frac{K}{n}\sum_{i\in\mathcal{I}_k} w_3(\bar{\boldsymbol{\beta}}^\intercal \mathbf{X}_i + \nu \boldsymbol{\Delta}^\intercal \mathbf{X}_i)(\boldsymbol{\Delta}^\intercal \mathbf{X}_i)^2 \tag{A.87}$$

uniformly for $\|\boldsymbol{\Delta}\|_2 \leq 1$ and $\nu \in [0,1]$ (Negahban et al., 2010, Proof of Proposition 2). Under Assumptions 6a and 6e, the lower bound is given by

$$\frac{K}{n}\sum_{i\in\mathcal{I}_k} w_3(\bar{\boldsymbol{\beta}}^\intercal \mathbf{X}_i + \nu \boldsymbol{\Delta}^\intercal \mathbf{X}_i)(\boldsymbol{\Delta}^\intercal \mathbf{X}_i)^2 \geq \kappa_1 \|\boldsymbol{\Delta}\|_2 \left\{ \|\boldsymbol{\Delta}\|_2 - \kappa_2 \sqrt{\log(p)/n}\|\boldsymbol{\Delta}\|_1 \right\} \tag{A.88}$$

for all $\|\boldsymbol{\Delta}\|_2 \leq 1$ and $\nu \in [0,1]$ with absolute constants κ_1 and κ_2. Under Assumption 6d, the restricted strong convexity for $\ell_k(\cdot; \widehat{\boldsymbol{\gamma}}^{(\mathrm{k})})$ can be also established by analyzing the same quantity in (A.87)

$$\begin{aligned}\ell_k(\bar{\boldsymbol{\beta}} + \boldsymbol{\Delta}; \widehat{\boldsymbol{\gamma}}^{(\mathrm{k})}) - \ell_k(\bar{\boldsymbol{\beta}}; \widehat{\boldsymbol{\gamma}}^{(\mathrm{k})}) - \boldsymbol{\Delta}^\intercal \dot{\boldsymbol{\ell}}(\bar{\boldsymbol{\beta}}; \widehat{\boldsymbol{\gamma}}^{(\mathrm{k})}) =& \frac{K}{n}\sum_{i\in\mathcal{I}_k} w_1(\mathbf{X}_i^\intercal \widehat{\boldsymbol{\gamma}}^{(\mathrm{k})}) w_3(\bar{\boldsymbol{\beta}}^\intercal \mathbf{X}_i + \nu \boldsymbol{\Delta}^\intercal \mathbf{X}_i)(\boldsymbol{\Delta}^\intercal \mathbf{X}_i)^2 \\ \geq& \frac{K}{n}\sum_{i\in\mathcal{I}_k} M^{-1} w_3(\bar{\boldsymbol{\beta}}^\intercal \mathbf{X}_i + \nu \boldsymbol{\Delta}^\intercal \mathbf{X}_i)(\boldsymbol{\Delta}^\intercal \mathbf{X}_i)^2\end{aligned} \tag{A.89}$$

Applying the lower bound in (A.88) to (A.89) at $\boldsymbol{\Delta} = t\boldsymbol{\delta}$, we obtain

$$\ell_k(\bar{\boldsymbol{\beta}} + t\boldsymbol{\delta}; \widehat{\boldsymbol{\gamma}}^{(\mathrm{k})}) - \ell_k(\bar{\boldsymbol{\beta}}; \widehat{\boldsymbol{\gamma}}^{(\mathrm{k})}) - t\boldsymbol{\delta}^\intercal \dot{\boldsymbol{\ell}}(\bar{\boldsymbol{\beta}}; \widehat{\boldsymbol{\gamma}}^{(\mathrm{k})}) \geq t^2\kappa_1/M - t^2\kappa_1\kappa_2/M\sqrt{\log(p)/n}\|\boldsymbol{\delta}\|_1. \tag{A.90}$$

Combining (A.85) and (A.90), we obtain

$$t\kappa_1/M \leq \lambda\|\boldsymbol{\delta}\|_1 - \frac{1}{K}\sum_{k=1}^{K} \boldsymbol{\delta}^\intercal \dot{\boldsymbol{\ell}}_k(\bar{\boldsymbol{\beta}}; \widehat{\boldsymbol{\gamma}}^{(\mathrm{k})}) + t\kappa_1\kappa_2/M\sqrt{\log(p)/n}\|\boldsymbol{\delta}\|_1. \tag{A.91}$$

We decompose $\frac{1}{K}\sum_{k=1}^{K} \boldsymbol{\delta}^\intercal \dot{\boldsymbol{\ell}}_k(\bar{\boldsymbol{\beta}}; \widehat{\boldsymbol{\gamma}}^{(\mathrm{k})})$

$$\left|\frac{1}{K}\sum_{k=1}^{K} \boldsymbol{\delta}^\intercal \dot{\boldsymbol{\ell}}_k(\bar{\boldsymbol{\beta}}; \widehat{\boldsymbol{\gamma}}^{(\mathrm{k})})\right| = \left|\frac{\boldsymbol{\delta}^\intercal}{K}\sum_{k=1}^{K} \dot{\boldsymbol{\ell}}_k(\bar{\boldsymbol{\beta}}; \widehat{\boldsymbol{\gamma}}^{(\mathrm{k})}) - \mathbb{E}_{i\in\mathcal{I}_k}\left[\dot{\boldsymbol{\ell}}_k(\bar{\boldsymbol{\beta}}; \widehat{\boldsymbol{\gamma}}^{(\mathrm{k})}) \mid \mathscr{D}_k^c\right]\right.$$

$$
\begin{aligned}
&+\frac{\boldsymbol{\delta}^{\mathsf{T}}}{K}\sum_{k=1}^{K}\mathbb{E}_{i\in\mathcal{I}_k}\left[\dot{\boldsymbol{\ell}}_k(\bar{\boldsymbol{\beta}};\widehat{\boldsymbol{\gamma}}^{(\mathsf{k})})\mid\mathscr{D}_k^c\right]\Bigg|\\
\leq&\|\boldsymbol{\delta}\|_1\left\|\frac{K}{N}\sum_{i\in\mathcal{I}_k}\dot{\boldsymbol{\ell}}_k(\bar{\boldsymbol{\beta}};\widehat{\boldsymbol{\gamma}}^{(\mathsf{k})})-\mathbb{E}_{i\in\mathcal{I}_k}\left[\dot{\boldsymbol{\ell}}_k(\bar{\boldsymbol{\beta}};\widehat{\boldsymbol{\gamma}}^{(\mathsf{k})})\mid\mathscr{D}_k^c\right]\right\|_\infty\\
&+\sup_{k=1,\dots,K}\left\|\mathbb{E}_{i\in\mathcal{I}_k}\left[\dot{\boldsymbol{\ell}}_k(\bar{\boldsymbol{\beta}};\widehat{\boldsymbol{\gamma}}^{(\mathsf{k})})\mid\mathscr{D}_k^c\right]\right\|_2.
\end{aligned}\tag{A.92}
$$

For "special case", the second term in (A.92) is zero, so the proofs up to **Case 2** following (A.99) can be skipped. Using the first order condition of optimality for $\bar{\boldsymbol{\beta}}$, we have

$$
\mathbb{E}\left[\dot{\boldsymbol{\ell}}_k(\bar{\boldsymbol{\beta}};\bar{\boldsymbol{\gamma}})\right]=0,
$$

so we can bound the second term in (A.92) for "in general" by

$$
\begin{aligned}
&\left\|\mathbb{E}_{i\in\mathcal{I}_k}\left[\dot{\boldsymbol{\ell}}_k(\bar{\boldsymbol{\beta}};\widehat{\boldsymbol{\gamma}}^{(\mathsf{k})})\mid\mathscr{D}_k^c\right]\right\|_2\\
=&\left\|\mathbb{E}_{i\in\mathcal{I}_k}\left[\dot{\boldsymbol{\ell}}_k(\bar{\boldsymbol{\beta}};\widehat{\boldsymbol{\gamma}}^{(\mathsf{k})})-\dot{\boldsymbol{\ell}}_k(\bar{\boldsymbol{\beta}};\bar{\boldsymbol{\gamma}})\mid\mathscr{D}_k^c\right]\right\|_2\\
=&\left\|\mathbb{E}_{i\in\mathcal{I}_k}[\{w_1(\mathbf{X}_i^{\mathsf{T}}\widehat{\boldsymbol{\gamma}}^{(\mathsf{k})})-w_1(\mathbf{X}_i^{\mathsf{T}}\bar{\boldsymbol{\gamma}})\}w_2(\bar{\boldsymbol{\beta}}^{\mathsf{T}}\mathbf{X}_i)\{g(\bar{\boldsymbol{\beta}}^{\mathsf{T}}\mathbf{X}_i)-Y_i\}\mid\mathscr{D}_k^c]\right\|_2.
\end{aligned}\tag{A.93}
$$

Using the Lipschitz condition for w_1 from Assumption 6d and other bounds from Assumptions 6b and 6d, we may bound (A.93) by

$$
\begin{aligned}
&\left\|\mathbb{E}_{i\in\mathcal{I}_k}[\{w_1(\mathbf{X}_i^{\mathsf{T}}\widehat{\boldsymbol{\gamma}}^{(\mathsf{k})})-w_1(\mathbf{X}_i^{\mathsf{T}}\bar{\boldsymbol{\gamma}})\}w_2(\bar{\boldsymbol{\beta}}^{\mathsf{T}}\mathbf{X}_i)\{g(\bar{\boldsymbol{\beta}}^{\mathsf{T}}\mathbf{X}_i)-Y_i\}\mid\mathscr{D}_k^c]\right\|_2\\
\leq&2M^3\mathbb{E}_{i\in\mathcal{I}_k}\{|\mathbf{X}_i^{\mathsf{T}}(\widehat{\boldsymbol{\gamma}}^{(\mathsf{k})}-\bar{\boldsymbol{\gamma}})|\mid\mathscr{D}_k^c\}
\end{aligned}\tag{A.94}
$$

Applying the sub-Gaussian property in Assumption 6a, we have

$$
\mathbb{E}_{i\in\mathcal{I}_k}\{|\mathbf{X}_i^{\mathsf{T}}(\widehat{\boldsymbol{\gamma}}^{(\mathsf{k})}-\bar{\boldsymbol{\gamma}})|\mid\mathscr{D}_k^c\}\leq\sqrt{\pi}\|\mathbf{X}_i^{\mathsf{T}}(\widehat{\boldsymbol{\gamma}}^{(\mathsf{k})}-\bar{\boldsymbol{\gamma}})\|_{\psi_2}\leq\sqrt{\pi}M\|\widehat{\boldsymbol{\gamma}}^{(\mathsf{k})}-\bar{\boldsymbol{\gamma}}\|_2.\tag{A.95}
$$

Collecting (A.92)-(A.95), we obtain

$$
\begin{aligned}
\left|\frac{1}{K}\sum_{k=1}^{K}\boldsymbol{\delta}^{\mathsf{T}}\dot{\boldsymbol{\ell}}_k(\bar{\boldsymbol{\beta}};\widehat{\boldsymbol{\gamma}}^{(\mathsf{k})})\right|\leq&\|\boldsymbol{\delta}\|_1\left\|\frac{K}{N}\sum_{i\in\mathcal{I}_k}\dot{\boldsymbol{\ell}}_k(\bar{\boldsymbol{\beta}};\widehat{\boldsymbol{\gamma}}^{(\mathsf{k})})-\mathbb{E}_{i\in\mathcal{I}_k}\left[\dot{\boldsymbol{\ell}}_k(\bar{\boldsymbol{\beta}};\widehat{\boldsymbol{\gamma}}^{(\mathsf{k})})\mid\mathscr{D}_k^c\right]\right\|_\infty\\
&+2\sqrt{\pi}M^4\|\widehat{\boldsymbol{\gamma}}^{(\mathsf{k})}-\bar{\boldsymbol{\gamma}}\|_2.
\end{aligned}\tag{A.96}
$$

Applying (A.96) and the definition of λ to (A.91), we get the preliminary estimation bound

$$
t\kappa_1/M\leq 4/3\lambda\|\boldsymbol{\delta}\|_1+2\sqrt{\pi}M^4\sup_{k=1,\dots,K}\|\widehat{\boldsymbol{\gamma}}^{(\mathsf{k})}-\bar{\boldsymbol{\gamma}}\|_2.\tag{A.97}
$$

Then, we separately analyze two cases.

Case 1:

$$
2\sqrt{\pi}M^4\sup_{k=1,\dots,K}\|\widehat{\boldsymbol{\gamma}}^{(\mathsf{k})}-\bar{\boldsymbol{\gamma}}\|_2\geq\lambda\|\boldsymbol{\delta}\|_1/3.
$$

In this case, the estimation error is dominated by $\widehat{\boldsymbol{\gamma}}^{(\mathsf{k})} - \bar{\boldsymbol{\gamma}}$. We simply have from (A.97)

$$t\kappa_1/M \le 10\sqrt{\pi}M^4 \sup_{k=1,\dots,K} \|\widehat{\boldsymbol{\gamma}}^{(\mathsf{k})} - \bar{\boldsymbol{\gamma}}\|_2.$$

Thus, we have

$$\|\widehat{\boldsymbol{\beta}} - \bar{\boldsymbol{\beta}}\|_2 \le 10\sqrt{\pi}M^5/\kappa_1 \sup_{k=1,\dots,K} \|\widehat{\boldsymbol{\gamma}}^{(\mathsf{k})} - \bar{\boldsymbol{\gamma}}\|_2. \tag{A.98}$$

Case 2:

$$2\sqrt{\pi}M^4 \sup_{k=1,\dots,K} \|\widehat{\boldsymbol{\gamma}}^{(\mathsf{k})} - \bar{\boldsymbol{\gamma}}\|_2 \le \lambda\|\boldsymbol{\delta}\|_1/3. \tag{A.99}$$

In this case, the estimation error is comparable to the situation that we have the asymptotic weights $w_1(\bar{\boldsymbol{\gamma}}^\intercal \mathbf{X}_i)$. Thus, the sparsity of $\bar{\boldsymbol{\beta}}$ may affect the estimation error.

Following the typical approach to establish the cone condition for $\boldsymbol{\delta}$, we analyze the symmetrized Bregman's divergence,

$$(\widehat{\boldsymbol{\beta}} - \bar{\boldsymbol{\beta}})^\intercal \frac{1}{K}\sum_{k=1}^K \{\dot{\boldsymbol{\ell}}_k(\widehat{\boldsymbol{\beta}}; \widehat{\boldsymbol{\gamma}}^{(\mathsf{k})}) - \dot{\boldsymbol{\ell}}_k(\bar{\boldsymbol{\beta}}; \widehat{\boldsymbol{\gamma}}^{(\mathsf{k})})\} = \|\widehat{\boldsymbol{\beta}} - \bar{\boldsymbol{\beta}}\|_2 \boldsymbol{\delta}^\intercal \frac{1}{K}\sum_{k=1}^K \{\dot{\boldsymbol{\ell}}_k(\widehat{\boldsymbol{\beta}}; \widehat{\boldsymbol{\gamma}}^{(\mathsf{k})}) - \dot{\boldsymbol{\ell}}_k(\bar{\boldsymbol{\beta}}; \widehat{\boldsymbol{\gamma}}^{(\mathsf{k})})\}. \tag{A.100}$$

Due to the convexity of the quadratic loss $\ell(\boldsymbol{\gamma})$ from (A.83), the symmetrized Bregman's divergence (A.100) is nonnegative. Denote the indices set of nonzero coefficient in $\bar{\boldsymbol{\beta}}$ as $\mathcal{O} = \{j : \bar{\beta}_j \neq 0\}$. We denote the $\boldsymbol{\delta}_{\mathcal{O}}$ and $\boldsymbol{\delta}_{\mathcal{O}^c}$ as the sub-vectors for $\boldsymbol{\delta}$ at positions in $\mathcal{O}$ and at positions not in $\mathcal{O}$, respectively. The solution $\widehat{\boldsymbol{\beta}}$ satisfies the KKT condition

$$\left\| \frac{1}{K}\sum_{k=1}^K \dot{\boldsymbol{\ell}}_k(\widehat{\boldsymbol{\beta}}; \widehat{\boldsymbol{\gamma}}^{(\mathsf{k})}) \right\|_\infty \le \lambda,\ \frac{1}{K}\sum_{k=1}^K \dot{\ell}_k(\widehat{\boldsymbol{\beta}}; \widehat{\boldsymbol{\gamma}}^{(\mathsf{k})})_j = -\lambda\,\mathrm{sign}(\widehat{\beta}_j),\ j : \widehat{\beta}_j \neq 0.$$

From the KKT condition and the definitions of $\boldsymbol{\delta}$ and $\mathcal{O}$, we have

$$\delta_j \frac{1}{K}\sum_{k=1}^K \dot{\ell}_k(\widehat{\boldsymbol{\beta}}; \widehat{\boldsymbol{\gamma}}^{(\mathsf{k})})_j \le |\delta_j|\lambda,\ j \in \mathcal{O};\ \delta_j \frac{1}{K}\sum_{k=1}^K \dot{\ell}_k(\widehat{\boldsymbol{\beta}}; \widehat{\boldsymbol{\gamma}}^{(\mathsf{k})})_j = \frac{-\widehat{\beta}_j \lambda\,\mathrm{sign}(\widehat{\beta}_j)}{\|\widehat{\boldsymbol{\beta}} - \bar{\boldsymbol{\beta}}\|_2} = -\lambda|\delta_j|,\ j \in \mathcal{O}^c. \tag{A.101}$$

Applying the (A.101) to (A.100), we have the upper bound,

$$\begin{aligned}
&\boldsymbol{\delta}^\intercal \frac{1}{K}\sum_{k=1}^K \{\dot{\boldsymbol{\ell}}_k(\widehat{\boldsymbol{\beta}}; \widehat{\boldsymbol{\gamma}}^{(\mathsf{k})}) - \dot{\boldsymbol{\ell}}_k(\bar{\boldsymbol{\beta}}; \widehat{\boldsymbol{\gamma}}^{(\mathsf{k})})\} \\
=& \sum_{j\in\mathcal{O}} \delta_j \frac{1}{K}\sum_{k=1}^K \dot{\ell}_k(\widehat{\boldsymbol{\beta}}; \widehat{\boldsymbol{\gamma}}^{(\mathsf{k})})_j + \sum_{j\in\mathcal{O}^c} \delta_j \frac{1}{K}\sum_{k=1}^K \dot{\ell}_k(\widehat{\boldsymbol{\beta}}; \widehat{\boldsymbol{\gamma}}^{(\mathsf{k})})_j - \boldsymbol{\delta}^\intercal \sum_{k=1}^K \dot{\boldsymbol{\ell}}_k(\bar{\boldsymbol{\beta}}; \widehat{\boldsymbol{\gamma}}^{(\mathsf{k})}) \\
\le& \lambda \sum_{j\in\mathcal{O}} |\delta_j| - \lambda \sum_{j\in\mathcal{O}^c} |\delta_j| + \left| \boldsymbol{\delta}^\intercal \sum_{k=1}^K \dot{\boldsymbol{\ell}}_k(\bar{\boldsymbol{\beta}}; \widehat{\boldsymbol{\gamma}}^{(\mathsf{k})}) \right|.
\end{aligned}$$

Then, we apply (A.96), the definition of λ and (A.99),

$$0 \le \lambda\|\boldsymbol{\delta}_{\mathcal{O}}\|_1 - \lambda\|\boldsymbol{\delta}_{\mathcal{O}^c}\|_1 + \frac{2}{3}\lambda\|\boldsymbol{\delta}\|_1.$$

Therefore, we can bound the L_1 norm of $\boldsymbol{\delta}$ by the cone property,

$$\|\boldsymbol{\delta}\|_1 \le 6\lambda\|\boldsymbol{\delta}_{\mathcal{O}}\|_1 \le 6\sqrt{s}\|\boldsymbol{\delta}\|_2 = 6\sqrt{s}. \tag{A.102}$$

Applying (A.99) and (A.102) to (A.97), we have the other bound for the estimation error

$$t\kappa_1/M \le 5/3\lambda\|\boldsymbol{\delta}\|_1 \le 10M\sqrt{s}\lambda,\ \|\widehat{\boldsymbol{\beta}} - \bar{\boldsymbol{\beta}}\|_2 \le 10M^2/\kappa_1\sqrt{s}\lambda. \tag{A.103}$$

The "special case" estimation error is directly given by (A.103)

$$\|\widehat{\boldsymbol{\beta}} - \bar{\boldsymbol{\beta}}\|_2 = O_p\left(\sqrt{s\log(p)/n}\right).$$

For "in general", we combine the bounds from the two cases (A.98) and (A.103),

$$\begin{aligned}\|\widehat{\boldsymbol{\beta}} - \bar{\boldsymbol{\beta}}\|_2 \le& \max\left\{10\sqrt{\pi}M^5/\kappa_1 \sup_{k=1,\dots,K}\|\widehat{\boldsymbol{\gamma}}^{(\mathsf{k})} - \bar{\boldsymbol{\gamma}}\|_2, 10M^2/\kappa_1\sqrt{s}\lambda\right\} \\ =& O_p\left(\sqrt{s\log(p)/n} + \sup_{k=1,\dots,K}\|\widehat{\boldsymbol{\gamma}}^{(\mathsf{k})} - \bar{\boldsymbol{\gamma}}\|_2\right).\end{aligned}$$

■

Appendix E. Additional Technical Details

E1 Definitions

Definition A22 (Hölder class) *A function $f(\mathbf{x})$ defined over $[-M, M]^d$ is Hölder class s if*

$$\sup_{\mathbf{x}_1,\mathbf{x}_2\in[-M,M]^d} \sup_{\substack{a_1,\dots,a_d\in\mathbb{N} \\ a_1+\dots+a_d=[s]}} \left|\frac{\partial^{[s]}}{\partial x_1^{a_1}\dots x_d^{a_d}}\{f(\mathbf{x}_1) - f(\mathbf{x}_2)\}\right| \|\mathbf{x}_1 - \mathbf{x}_2\|_2^{[s-1]-s} < \infty.$$

We note the maximal Hölder class as $\mathcal{H}(f) = \sup\{s : f \text{ is Hölder class } s\}$.

We adopt the following definition of sub-Gaussian and sub-exponential random variables.

Definition A23 (Sub-Gaussian and Sub-Exponential Random Variables) *The sub-Gaussian parameter for a random variable V is defined as*

$$\|V\|_{\psi_2} = \inf\left\{\sigma > 0 : \mathbb{E}(e^{V^2/\sigma^2}) \le 2\right\}.$$

The random variable V is sub-Gaussian if $\|V\|_{\psi_2}$ is finite. The sub-Gaussian parameter for a random vector $\mathbf{U}$ is defined as

$$\|\mathbf{U}\|_{\psi_2} = \sup_{\|\mathbf{v}\|_2=1}\|\mathbf{v}^\mathsf{T}\mathbf{U}\|_{\psi_2}.$$

The sub-Gaussian parameter for a random variable V is defined as

$$\|V\|_{\psi_1} = \inf\left\{\nu > 0 : \mathbb{E}(e^{|V|/\nu}) \le 2\right\}.$$

The random variable V is sub-exponential if $\|V\|_{\psi_1}$ is finite.

E2 Geometry of model tangent space

The nonparametric model for observed data is thus

$$\begin{aligned}\mathcal{M}_{obs} =\Big\{ & f_{\mathbf{X},A,Y,\mathbf{S},R}(\mathbf{x},a,t,\mathbf{s},r) = f_{\mathbf{X}}(\mathbf{x})[\pi(a,\mathbf{x})^a f_{Y|A,\mathbf{X}}(y|a,\mathbf{x}) \\ & \times f_{\mathbf{S}|Y,A,\mathbf{X}}(s|y,a,\mathbf{x})m(\mathbf{x})]^r \big(f_{\mathbf{S}|\mathbf{X}}(\mathbf{s}|\mathbf{x})\{1-m(x)\}\big)^{1-r} : \\ & f_{\mathbf{X}}, \pi, f_{Y|A,\mathbf{X}}, f_{\mathbf{S}|Y,A,\mathbf{X}}, m \text{ are arbitrary pdfs/pmfs}\Big\}. \end{aligned} \tag{A.104}$$

We consider the parametric sub-model indexed by parameter $\boldsymbol{\gamma}$

$$\begin{aligned}\mathcal{M}_{par} =\Big\{ & f_{\mathbf{X},A,Y,\mathbf{S},R}(\mathbf{x},a,t,\mathbf{s},r;\boldsymbol{\gamma}) = f_{\mathbf{X}}(\mathbf{x};\boldsymbol{\gamma})[\pi(a,\mathbf{x};\boldsymbol{\gamma}) f_{Y|A,\mathbf{X}}(y|a,\mathbf{x};\boldsymbol{\gamma}) \\ & \times f_{\mathbf{S}|Y,A,\mathbf{X}}(s|y,a,\mathbf{x};\boldsymbol{\gamma})m^*(\mathbf{x})]^r \\ & \times \big[f_{\mathbf{S}|\mathbf{X}}(\mathbf{s}|\mathbf{x};\boldsymbol{\gamma})\{1-m^*(\mathbf{x})\}\big]^{1-r} : \boldsymbol{\gamma}\in\Gamma\Big\}, \end{aligned} \tag{A.105}$$

$$f_{\mathbf{S}|\mathbf{X}}(\mathbf{s}|\mathbf{x};\boldsymbol{\gamma}) = \sum_{a\in\mathbb{N}}\int_{y\in\mathbb{R}} \pi(a,\mathbf{x};\boldsymbol{\gamma}) f_{Y|A,\mathbf{X}}(y|a,\mathbf{x};\boldsymbol{\gamma}) f_{\mathbf{S}|Y,A,\mathbf{X}}(\mathbf{s}|y,a,\mathbf{x};\boldsymbol{\gamma})dy. \tag{A.106}$$

where $\boldsymbol{\gamma}=\boldsymbol{\gamma}^*$ indicates the true parameter.

Utilizing the identity

$$\begin{aligned}& \frac{\partial \log\{f_{\mathbf{S}|\mathbf{X}}(\mathbf{S}|\mathbf{X};\boldsymbol{\gamma})\}}{\partial\boldsymbol{\gamma}}\bigg|_{\boldsymbol{\gamma}=\boldsymbol{\gamma}^*} \\ =& \sum_{a\in\mathbb{N}}\int_{y\in\mathbb{R}} \frac{\partial}{\partial\boldsymbol{\gamma}}\pi(a,\mathbf{X};\boldsymbol{\gamma})\bigg|_{\boldsymbol{\gamma}=\boldsymbol{\gamma}^*} \frac{f_{Y|A,\mathbf{X}}(y|a,\mathbf{X};\boldsymbol{\gamma}^*) f_{\mathbf{S}|Y,A,\mathbf{X}}(\mathbf{S}|y,a,\mathbf{X};\boldsymbol{\gamma}^*)}{f_{\mathbf{S}|\mathbf{X}}(\mathbf{X}|\mathbf{X};\boldsymbol{\gamma}^*)}dy \\ & \sum_{a\in\mathbb{N}}\int_{y\in\mathbb{R}} \frac{\partial}{\partial\boldsymbol{\gamma}} f_{Y|A,\mathbf{X}}(y|a,\mathbf{X};\boldsymbol{\gamma})\bigg|_{\boldsymbol{\gamma}=\boldsymbol{\gamma}^*} \frac{\pi(a,\mathbf{X};\boldsymbol{\gamma}^*) f_{\mathbf{S}|Y,A,\mathbf{X}}(\mathbf{S}|y,a,\mathbf{X};\boldsymbol{\gamma}^*)}{f_{\mathbf{S}|\mathbf{X}}(\mathbf{X}|\mathbf{X};\boldsymbol{\gamma}^*)}dy \\ & +\sum_{a\in\mathbb{N}}\int_{y\in\mathbb{R}} \frac{\partial}{\partial\boldsymbol{\gamma}} f_{\mathbf{S}|Y,A,\mathbf{X}}(\mathbf{S}|y,a,\mathbf{X};\boldsymbol{\gamma})\bigg|_{\boldsymbol{\gamma}=\boldsymbol{\gamma}^*} \frac{\pi(a,\mathbf{X};\boldsymbol{\gamma}^*) f_{Y|A,\mathbf{X}}(y|a,\mathbf{X};\boldsymbol{\gamma}^*)}{f_{\mathbf{S}|\mathbf{X}}(\mathbf{X}|\mathbf{X};\boldsymbol{\gamma}^*)}dy \\ =& \sum_{a\in\mathbb{N}}\int_{y\in\mathbb{R}} \frac{\partial}{\partial\boldsymbol{\gamma}}[\log\{\pi(a,\mathbf{X};\boldsymbol{\gamma})\} + \log\{f_{Y|A,\mathbf{X}}(y|a,\mathbf{X};\boldsymbol{\gamma})\} + \log\{f_{\mathbf{S}|Y,A,\mathbf{X}}(\mathbf{S}|y,a,\mathbf{X};\boldsymbol{\gamma})\}]\bigg|_{\boldsymbol{\gamma}=\boldsymbol{\gamma}^*} \\ & \quad \times \frac{\pi(a,\mathbf{X};\boldsymbol{\gamma}^*) f_{Y|A,\mathbf{X}}(y|a,\mathbf{X};\boldsymbol{\gamma}^*) f_{\mathbf{S}|Y,A,\mathbf{X}}(\mathbf{S}|y,a,\mathbf{X};\boldsymbol{\gamma}^*)}{f_{\mathbf{S}|\mathbf{X}}(\mathbf{X}|\mathbf{X};\boldsymbol{\gamma}^*)}dy \\ =& \mathbb{E}\left[\frac{\partial}{\partial\boldsymbol{\gamma}}\log\{\pi(A,\mathbf{X};\boldsymbol{\gamma})\}\bigg|_{\boldsymbol{\gamma}=\boldsymbol{\gamma}^*} \mid \mathbf{S},\mathbf{X}\right] + \mathbb{E}\left[\frac{\partial}{\partial\boldsymbol{\gamma}}\log\{f_{Y|A,\mathbf{X}}(Y|A,\mathbf{X};\boldsymbol{\gamma})\}\bigg|_{\boldsymbol{\gamma}=\boldsymbol{\gamma}^*} \mid \mathbf{S},\mathbf{X}\right] \\ & + \mathbb{E}\left[\frac{\partial}{\partial\boldsymbol{\gamma}}\log\{f_{\mathbf{S}|Y,A,\mathbf{X}}(\mathbf{S}|Y,A,\mathbf{X};\boldsymbol{\gamma})\}\bigg|_{\boldsymbol{\gamma}=\boldsymbol{\gamma}^*} \mid \mathbf{S},\mathbf{X}\right], \end{aligned}$$

we express the score vector of the parametric sub-model as

$$\boldsymbol{\Psi}(\mathbf{X},A,\mathbf{S},Y,R) = \frac{\partial \log\{f_{\mathbf{X},A,Y,\mathbf{S},R}(\mathbf{X},A,Y,\mathbf{S},R;\boldsymbol{\gamma})\}}{\partial\boldsymbol{\gamma}}\bigg|_{\boldsymbol{\gamma}=\boldsymbol{\gamma}^*}$$

$$=\boldsymbol{\Psi}_{\mathbf{X}}(\mathbf{X};\boldsymbol{\gamma}^*)+\boldsymbol{\Psi}_{A}(R,\mathbf{X};\boldsymbol{\gamma}^*)+\boldsymbol{\Psi}_{Y}(R,A,\mathbf{X};\boldsymbol{\gamma}^*)+\boldsymbol{\Psi}_{\mathbf{S}}(R,Y,A,\mathbf{X};\boldsymbol{\gamma}^*) \tag{A.107}$$

where the components are

$$\begin{aligned}
\boldsymbol{\Psi}_{\mathbf{X}}(\mathbf{X};\boldsymbol{\gamma}^*) &= \frac{\partial \log\{f_{\mathbf{X}}(\mathbf{X};\boldsymbol{\gamma})\}}{\partial \boldsymbol{\gamma}}\bigg|_{\boldsymbol{\gamma}=\boldsymbol{\gamma}^*},\\
\boldsymbol{\Psi}_{A}(R,\mathbf{X};\boldsymbol{\gamma}^*) &= R\,\frac{\partial \log\{\pi(A|\mathbf{X};\boldsymbol{\gamma})\}}{\partial \boldsymbol{\gamma}}\bigg|_{\boldsymbol{\gamma}=\boldsymbol{\gamma}^*}+(1-R)\mathbb{E}\left[\frac{\partial \log\{\pi(A|\mathbf{X};\boldsymbol{\gamma})\}}{\partial \boldsymbol{\gamma}}\bigg|_{\boldsymbol{\gamma}=\boldsymbol{\gamma}^*}\mid \mathbf{S},\mathbf{X}\right],\\
\boldsymbol{\Psi}_{Y}(R,A,\mathbf{X};\boldsymbol{\gamma}^*) &= R\,\frac{\partial \log\{f_{Y|A,\mathbf{X}}(Y|A,\mathbf{X};\boldsymbol{\gamma})\}}{\partial \boldsymbol{\gamma}}\bigg|_{\boldsymbol{\gamma}=\boldsymbol{\gamma}^*}\\
&\quad+(1-R)\mathbb{E}\left[\frac{\partial \log\{f_{Y|A,\mathbf{X}}(Y|A,\mathbf{X};\boldsymbol{\gamma})\}}{\partial \boldsymbol{\gamma}}\bigg|_{\boldsymbol{\gamma}=\boldsymbol{\gamma}^*}\mid \mathbf{S},\mathbf{X}\right],\\
\boldsymbol{\Psi}_{\mathbf{S}}(R,\mathbf{S},A,\mathbf{X};\boldsymbol{\gamma}^*) &= R\,\frac{\partial \log\{f_{\mathbf{S}|Y,A,\mathbf{X}}(\mathbf{S}|Y,A,\mathbf{X};\boldsymbol{\gamma})\}}{\partial \boldsymbol{\gamma}}\bigg|_{\boldsymbol{\gamma}=\boldsymbol{\gamma}^*}\\
&\quad+(1-R)\mathbb{E}\left[\frac{\partial \log\{f_{\mathbf{S}|Y,A,\mathbf{X}}(\mathbf{S}|Y,A,\mathbf{X};\boldsymbol{\gamma})\}}{\partial \boldsymbol{\gamma}}\bigg|_{\boldsymbol{\gamma}=\boldsymbol{\gamma}^*}\mid \mathbf{S},\mathbf{X}\right].
\end{aligned} \tag{A.108}$$

Let $\mathscr{H}$ be the Hilbert space of mean zero finite variance random variables measurable to $\sigma\{\mathbf{X}, AR, \mathbf{S}, YR, R\}$. The nuisance parameter tangent space is spanned by $\boldsymbol{\Psi}(\mathbf{X}, A, \mathbf{S}, Y, R)$,

$$\Lambda = \{\mathbf{v}^{\intercal}\boldsymbol{\Psi}(\mathbf{X}, A, \mathbf{S}, Y, R) : \mathcal{M}_{par} \subset \mathcal{M}_{obs}\}. \tag{A.109}$$

According to the decomposition (A.107), we can decompose the nuisance parameter tangent space

$$\Lambda = \Lambda_{\mathbf{X}} + \Lambda_A + \Lambda_Y + \Lambda_{\mathbf{S}}.$$

We derive $\Lambda_{\mathbf{X}}$, Λ_A, Λ_Y and $\Lambda_{\mathbf{S}}$ as

$$\begin{aligned}
\Lambda_{\mathbf{X}} &= \{h(\mathbf{X}) \in \mathscr{H} : \mathbb{E}[h(\mathbf{X})] = 0\},\\
\Lambda_A &= \Big\{Rh(A,\mathbf{X}) + (1-R)\mathbb{E}[h(A,\mathbf{X}) \mid \mathbf{S},\mathbf{X}] \in \mathscr{H} : \mathbb{E}[h(A,\mathbf{X}) \mid \mathbf{X}] = 0\Big\},\\
\Lambda_Y &= \Big\{Rh(Y,A,\mathbf{X}) + (1-R)\mathbb{E}[h(Y,A,\mathbf{X}) \mid \mathbf{S},\mathbf{X}] \in \mathscr{H} : \mathbb{E}[h(Y,A,\mathbf{X}) \mid A,\mathbf{X}] = 0\Big\},\\
\Lambda_{\mathbf{S}} &= \Big\{Rh(\mathbf{S},Y,A,\mathbf{X}) + (1-R)\mathbb{E}[h(\mathbf{S},Y,A,\mathbf{X}) \mid \mathbf{S},\mathbf{X}] \in \mathscr{H} :\\
&\qquad \mathbb{E}[h(\mathbf{S},Y,A,\mathbf{X}) \mid Y,A,\mathbf{X}] = 0\Big\}.
\end{aligned} \tag{A.110}$$

Under the settings of Robins et al. (1994) and Kallus and Mao (2024), the scores for $f_{A|\mathbf{X}}$ and $f_{Y|A,\mathbf{X}}$ belong to two linear subspaces orthogonal to each other in $\mathscr{H}$, the Hilbert space of mean zero finite variance random variables. However, the two scores under $\mathcal{S}_{\mathsf{SSL}}$ belong to the linear subspaces Λ_A and Λ_Y which share a correlated component from the unlabeled data induced by the surrogates $\mathbf{S}$.

E3 B-spline Regression

Lemma A24 *Under the assumptions:*

1. $\mathbf{X} \in [0,1]^p$ *with density* $f_{\mathbf{X}}(\mathbf{x}) \in [1/M, M]$, $\forall \mathbf{x} \in [0,1]^p$;
2. $\mathbf{q}(\mathbf{x}) \in \mathbb{R}^b$ *is the vector of tensor product B-splines of order* κ *with knot spacing approximately proportional to the number of knots;*
3. $\inf_{\|\mathbf{v}\|_2=1} \mathbf{v}^\intercal \mathbb{E}\{\mathbf{q}(\mathbf{X})\mathbf{q}(\mathbf{X})^\intercal\}\mathbf{v} \geq 1/M$;
4. $\sup_{\mathbf{x}\in[0,1]^p} \|\mathbf{q}(\mathbf{x})\|_2 \leq M\sqrt{b}$;
5. $|Y| \leq M$;
6. $\mu_*(\mathbf{x}) = \mathbb{E}(Y \mid \mathbf{X} = \mathbf{x})$ *is Hölder of order* s.

Let $\bar{\mu}(\mathbf{x})$ *be the best linear approximation of* $\mu_*(\mathbf{x})$ *with basis* $\mathbf{q}(\mathbf{x})$

$$\bar{\mu}(\mathbf{x}) = \mathbf{q}(\mathbf{x})^\intercal \left[\mathbb{E}\{\mathbf{q}(\mathbf{X})\mathbf{q}(\mathbf{X})^\intercal\}\right]^{-1} \mathbb{E}\{\mathbf{q}(\mathbf{X})Y\},$$

and its estimator with n *samples*

$$\widehat{\mu}(\mathbf{x}) = \mathbf{q}(\mathbf{x})^\intercal \left\{\frac{1}{n}\sum_{i=1}^n \mathbf{q}(\mathbf{X}_i)\mathbf{q}(\mathbf{X}_i)^\intercal\right\}^{-1} \frac{1}{n}\sum_{i=1}^n \mathbf{q}(\mathbf{X}_i)Y_i$$

The approximation error is

$$\|\bar{\mu} - \mu_*\|_2 = O(b^{-\min\{1+\kappa, s\}/p}).$$

The estimation error with sample n *is*

$$\|\bar{\mu} - \widehat{\mu}\|_2 = O_p\left(\sqrt{b/n}\right).$$

See in Newey and Robins (2018) for example.

E4 Minimax Lower Bound

Lemma A25 *Denote the truncation at zero* $[\cdot]_+$ *and the normalizing constant* $C_{\mathbf{h}}$

$$\left[1 + \frac{\mathbf{h}_1^\intercal \mathbf{g}_1(\mathbf{z}_i, \mathbf{w}_i)}{\sqrt{n}} + \frac{\mathbf{h}_2^\intercal \mathbf{g}_2(\mathbf{w}_i)}{\sqrt{N}}\right]_+ = \max\left\{0, 1 + \frac{\mathbf{h}_1^\intercal \mathbf{g}_1(\mathbf{z}_i, \mathbf{w}_i)}{\sqrt{n}} + \frac{\mathbf{h}_2^\intercal \mathbf{g}_2(\mathbf{w}_i)}{\sqrt{N}}\right\},$$
$$C_{\mathbf{h}} = \mathbb{E}_*\left(\left[1 + \frac{\mathbf{h}_1^\intercal \mathbf{g}_1(\mathbf{Z}_i, \mathbf{W}_i)}{\sqrt{n}} + \frac{\mathbf{h}_2^\intercal \mathbf{g}_2(\mathbf{W}_i)}{\sqrt{N}}\right]_+\right)$$

and the two-way tilted density

$$f_{\mathbf{h}}(\mathbf{z}_i, \mathbf{w}_i) = f_*(\mathbf{z}_i, \mathbf{w}_i)\left[1 + \frac{\mathbf{h}_1^\intercal \mathbf{g}_1(\mathbf{z}_i, \mathbf{w}_i)}{\sqrt{n}} + \frac{\mathbf{h}_2^\intercal \mathbf{g}_2(\mathbf{w}_i)}{\sqrt{N}}\right]_+ / C_{\mathbf{h}}.$$

If $\mathbf{g}_1$ *and* $\mathbf{g}_2$ *has bounded variance under* f_**,*

$$\sup_{\|\mathbf{v}\|_2=1} \mathbb{E}_*[\{\mathbf{v}^\intercal \mathbf{g}_1(\mathbf{Z}_i, \mathbf{W}_i)\}^2] + \sup_{\|\mathbf{u}\|_2=1} \mathbb{E}_*[\{\mathbf{u}^\intercal \mathbf{g}_2(\mathbf{W}_i)\}^2] \le M,$$

the tilted density falls in the neighborhood in $\|\cdot\|_{\mathsf{TV}}$*,*

$$\|f_* - f_{\mathbf{h}}\|_{\mathsf{TV}} \le M\sqrt{\|\mathbf{h}_1\|_2^2/n + \|\mathbf{h}_2\|_2^2/N} + o(\|\mathbf{h}\|_2/\sqrt{n}). \tag{A.64}$$

Proof [Proof of Lemma A25] The proof extends Example 5 page 11 of Duchi (2021) to two-way tilted sub-models for characterizing semi-supervised learning setting. First we show that the normalizing constant approaches 1 at

$$C_{\mathbf{h}} = 1 + O(\|\mathbf{h}\|_2^2/n).$$

By definition of $C_{\mathbf{h}}$ and mean zero assumption for $\mathbf{g}_1$ and $\mathbf{g}_2$, we have the lower bound for $C_{\mathbf{h}}$

$$\begin{aligned}
C_{\mathbf{h}} =& \mathbb{E}_*\left(\left[1 + \frac{\mathbf{h}_1^\intercal \mathbf{g}_1(\mathbf{Z}_i, \mathbf{W}_i)}{\sqrt{n}} + \frac{\mathbf{h}_2^\intercal \mathbf{g}_2(\mathbf{W}_i)}{\sqrt{N}}\right]_+\right) \\
\ge& \mathbb{E}_*\left(1 + \frac{\mathbf{h}_1^\intercal \mathbf{g}_1(\mathbf{Z}_i, \mathbf{W}_i)}{\sqrt{n}} + \frac{\mathbf{h}_2^\intercal \mathbf{g}_2(\mathbf{W}_i)}{\sqrt{N}}\right) \\
=& 1 + \frac{\mathbf{h}_1^\intercal \mathbb{E}_*\{\mathbf{g}_1(\mathbf{Z}_i, \mathbf{W}_i)\}}{\sqrt{n}} + \frac{\mathbf{h}_2^\intercal \mathbb{E}_*\{\mathbf{g}_2(\mathbf{W}_i)\}}{\sqrt{N}} \\
=& 1.
\end{aligned} \tag{A.111}$$

Define the event of activated truncation

$$\Xi_i = \mathrm{I}\left\{1 + \frac{\mathbf{h}_1^\intercal \mathbf{g}_1(\mathbf{Z}_i, \mathbf{W}_i)}{\sqrt{n}} + \frac{\mathbf{h}_2^\intercal \mathbf{g}_2(\mathbf{W}_i)}{\sqrt{N}} < 0\right\}, \tag{A.112}$$

we may alternatively represent the tilt factor as

$$\begin{aligned}
&\left[1 + \frac{\mathbf{h}_1^\intercal \mathbf{g}_1(\mathbf{Z}_i, \mathbf{W}_i)}{\sqrt{n}} + \frac{\mathbf{h}_2^\intercal \mathbf{g}_2(\mathbf{W}_i)}{\sqrt{N}}\right]_+ \\
=& 1 + \frac{\mathbf{h}_1^\intercal \mathbf{g}_1(\mathbf{Z}_i, \mathbf{W}_i)}{\sqrt{n}} + \frac{\mathbf{h}_2^\intercal \mathbf{g}_2(\mathbf{W}_i)}{\sqrt{N}} - \Xi_i\left\{1 + \frac{\mathbf{h}_1^\intercal \mathbf{g}_1(\mathbf{Z}_i, \mathbf{W}_i)}{\sqrt{n}} + \frac{\mathbf{h}_2^\intercal \mathbf{g}_2(\mathbf{W}_i)}{\sqrt{N}}\right\}.
\end{aligned} \tag{A.113}$$

Using (A.113), we establish an upper bound of $C_{\mathbf{h}}$

$$\begin{aligned}
C_{\mathbf{h}} =& \mathbb{E}_*\left(1 + \frac{\mathbf{h}_1^\intercal \mathbf{g}_1(\mathbf{Z}_i, \mathbf{W}_i)}{\sqrt{n}} + \frac{\mathbf{h}_2^\intercal \mathbf{g}_2(\mathbf{W}_i)}{\sqrt{N}}\right) \\
&- \mathbb{E}_*\left(\Xi_i\left\{1 + \frac{\mathbf{h}_1^\intercal \mathbf{g}_1(\mathbf{Z}_i, \mathbf{W}_i)}{\sqrt{n}} + \frac{\mathbf{h}_2^\intercal \mathbf{g}_2(\mathbf{W}_i)}{\sqrt{N}}\right\}\right) \\
=& 1 + \mathbb{E}_*\left(\Xi_i\left|1 + \frac{\mathbf{h}_1^\intercal \mathbf{g}_1(\mathbf{Z}_i, \mathbf{W}_i)}{\sqrt{n}} + \frac{\mathbf{h}_2^\intercal \mathbf{g}_2(\mathbf{W}_i)}{\sqrt{N}}\right|\right) \\
\le& 1 + \mathbb{E}_*\left(\Xi_i\left|\frac{\mathbf{h}_1^\intercal \mathbf{g}_1(\mathbf{Z}_i, \mathbf{W}_i)}{\sqrt{n}} + \frac{\mathbf{h}_2^\intercal \mathbf{g}_2(\mathbf{W}_i)}{\sqrt{N}}\right|^2\right)
\end{aligned}$$

$$\leq 1 + 2\mathbb{E}_* \left(|\mathbf{h}_1^\intercal \mathbf{g}_1(\mathbf{Z}_i, \mathbf{W}_i)|^2/n + |\mathbf{h}_2^\intercal \mathbf{g}_2(\mathbf{W}_i)|^2/N \right). \tag{A.114}$$

Applying the bounded variance assumption for $\mathbf{g}_1$ and $\mathbf{g}_2$, we have the upper bound

$$\begin{aligned} C_{\mathbf{h}} \leq& 1 + 2\mathbb{E}_* \left(|\mathbf{h}_1^\intercal \mathbf{g}_1(\mathbf{Z}_i, \mathbf{W}_i)|^2/n + |\mathbf{h}_2^\intercal \mathbf{g}_2(\mathbf{W}_i)|^2/N \right) \\ \leq& 1 + 2\mathbb{E}_* \left(|\mathbf{h}_1^\intercal \mathbf{g}_1(\mathbf{Z}_i, \mathbf{W}_i)|^2 + |\mathbf{h}_2^\intercal \mathbf{g}_2(\mathbf{W}_i)|^2 \right)/n \\ \leq& 1 + 2M\|\mathbf{h}\|_2^2/n. \end{aligned} \tag{A.115}$$

Combining the lower bound (A.111) and upper bound (A.115) of C_h, we have shown

$$C_{\mathbf{h}} = 1 + O(\|\mathbf{h}\|_2^2/n) = 1 + o(\|\mathbf{h}\|_2/\sqrt{n}). \tag{A.116}$$

Then, we bound the distance in total variation

$$\begin{aligned} \|f_* - f_{\mathbf{h}}\|_{\text{TV}} =& \int f_*(\mathbf{z}, \mathbf{w}) \left| 1 - \left[1 + \frac{\mathbf{h}_1^\intercal \mathbf{g}_1(\mathbf{z}_i, \mathbf{w}_i)}{\sqrt{n}} + \frac{\mathbf{h}_2^\intercal \mathbf{g}_2(\mathbf{w}_i)}{\sqrt{N}} \right]_+ / C_{\mathbf{h}} \right| d\mathbf{z} d\mathbf{w} \\ =& \mathbb{E}_* \left\{ \left| 1 - \left[1 + \frac{\mathbf{h}_1^\intercal \mathbf{g}_1(\mathbf{z}_i, \mathbf{w}_i)}{\sqrt{n}} + \frac{\mathbf{h}_2^\intercal \mathbf{g}_2(\mathbf{w}_i)}{\sqrt{N}} \right]_+ / C_{\mathbf{h}} \right| \right\}. \end{aligned}$$

We decompose the tilted factor into 3 parts

$$\begin{aligned} & 1 - \left[1 + \frac{\mathbf{h}_1^\intercal \mathbf{g}_1(\mathbf{z}_i, \mathbf{w}_i)}{\sqrt{n}} + \frac{\mathbf{h}_2^\intercal \mathbf{g}_2(\mathbf{w}_i)}{\sqrt{N}} \right]_+ / C_{\mathbf{h}} \\ =& \underbrace{1 - 1/C_{\mathbf{h}}}_{T_1} + \underbrace{\left\{ \frac{\mathbf{h}_1^\intercal \mathbf{g}_1(\mathbf{z}_i, \mathbf{w}_i)}{\sqrt{n}} + \frac{\mathbf{h}_2^\intercal \mathbf{g}_2(\mathbf{w}_i)}{\sqrt{N}} \right\} / C_{\mathbf{h}}}_{T_2} \\ & + \underbrace{\left(1 + \frac{\mathbf{h}_1^\intercal \mathbf{g}_1(\mathbf{z}_i, \mathbf{w}_i)}{\sqrt{n}} + \frac{\mathbf{h}_2^\intercal \mathbf{g}_2(\mathbf{w}_i)}{\sqrt{N}} - \left[1 + \frac{\mathbf{h}_1^\intercal \mathbf{g}_1(\mathbf{z}_i, \mathbf{w}_i)}{\sqrt{n}} + \frac{\mathbf{h}_2^\intercal \mathbf{g}_2(\mathbf{w}_i)}{\sqrt{N}} \right]_+ \right) / C_{\mathbf{h}}}_{T_3} \end{aligned} \tag{A.117}$$

and evaluate their L_1-norm separately. Applying the order of $C_{\mathbf{h}}$ established in (A.116), we bound the L_1-norm of T_1

$$\mathbb{E}_*\{|T_1|\} = |1 - 1/C_{\mathbf{h}}| = 1 + o(\|\mathbf{h}\|_2/\sqrt{n}). \tag{A.118}$$

Applying the bounded variance assumption for $\mathbf{g}_1$ and $\mathbf{g}_2$ and the rate of $C_{\mathbf{h}}$, we bound the L_1-norm of T_2

$$\begin{aligned} \mathbb{E}_*\{|T_2|\} =& \mathbb{E}_* \left\{ \left| \frac{\mathbf{h}_1^\intercal \mathbf{g}_1(\mathbf{Z}_i, \mathbf{W}_i)}{\sqrt{n}} + \frac{\mathbf{h}_2^\intercal \mathbf{g}_2(\mathbf{W}_i)}{\sqrt{N}} \right| \right\} / C_{\mathbf{h}} \\ \leq& \sqrt{\mathbb{E}_* \left[\{\mathbf{h}_1^\intercal \mathbf{g}_1(\mathbf{Z}_i, \mathbf{W}_i)\}^2 / n \right]} / C_{\mathbf{h}} + \sqrt{\mathbb{E}_* \left[\{\mathbf{h}_2^\intercal \mathbf{g}_2(\mathbf{W}_i)\}^2 / N \right]} / C_{\mathbf{h}} \\ \leq& M\sqrt{\|\mathbf{h}_1\|_2^2/n + \|\mathbf{h}_2\|_2^2/N} / C_{\mathbf{h}} \\ =& M\sqrt{\|\mathbf{h}_1\|_2^2/n + \|\mathbf{h}_2\|_2^2/N} + o(\|\mathbf{h}\|_2/\sqrt{n}). \end{aligned} \tag{A.119}$$

For T_3, we repeat the analysis of upper bound for $C_{\mathbf{h}}$ (A.114) and (A.115) through alternative representation (A.113) with truncation indicator Ξ defined in (A.112),

$$\begin{aligned}
&\mathbb{E}_*\{|T_3|\}\\
=&\mathbb{E}_*\left(\left|1+\frac{\mathbf{h}_1^\intercal\mathbf{g}_1(\mathbf{z}_i,\mathbf{w}_i)}{\sqrt{n}}+\frac{\mathbf{h}_2^\intercal\mathbf{g}_2(\mathbf{w}_i)}{\sqrt{N}}-\left[1+\frac{\mathbf{h}_1^\intercal\mathbf{g}_1(\mathbf{z}_i,\mathbf{w}_i)}{\sqrt{n}}+\frac{\mathbf{h}_2^\intercal\mathbf{g}_2(\mathbf{w}_i)}{\sqrt{N}}\right]_+\right|\right)/C_{\mathbf{h}}\\
=&\mathbb{E}_*\left\{\Xi_i\left|1+\frac{\mathbf{h}_1^\intercal\mathbf{g}_1(\mathbf{z}_i,\mathbf{w}_i)}{\sqrt{n}}+\frac{\mathbf{h}_2^\intercal\mathbf{g}_2(\mathbf{w}_i)}{\sqrt{N}}\right|\right\}/C_{\mathbf{h}}\\
\leq&2M\|\mathbf{h}\|_2^2/n\\
=&o\left(\|\mathbf{h}\|_2/\sqrt{n}\right). \qquad \text{(A.120)}
\end{aligned}$$

Combining the rates (A.118)-(A.120) and the decomposition (A.117), we have shown

$$\|f_*-f_{\mathbf{h}}\|_{\mathsf{TV}}\leq\mathbb{E}_*(|T_1|)+\mathbb{E}_*(|T_2|)+\mathbb{E}_*(|T_3|)\leq M\sqrt{\|\mathbf{h}_1\|_2^2/n+\|\mathbf{h}_2\|_2^2/N}+o(\|\mathbf{h}\|_2/\sqrt{n}).$$

■

Lemma A26 *Consider the settings detailed in the proof of Theorem 13, i. e. the two-way tilted density for* $\mathbf{Z},\mathbf{W}\mid\mathbf{h}$

$$f_{\mathbf{h}}(\mathbf{z}_i,\mathbf{w}_i)=f_*(\mathbf{z}_i,\mathbf{w}_i)\left[1+\frac{\mathbf{h}_1^\intercal\mathbf{g}_1(\mathbf{z}_i,\mathbf{w}_i)}{\sqrt{n}}+\frac{\mathbf{h}_2^\intercal\mathbf{g}_2(\mathbf{w}_i)}{\sqrt{N}}\right]_+/C_{\mathbf{h}}$$

with the truncated Gaussian prior for $\mathbf{h}$*,*

$$(\mathbf{h}_1^\intercal,\mathbf{h}_2^\intercal)^\intercal\sim p(\mathbf{h};c,\mathbb{A})=\frac{\phi(\mathbf{h},\mathbf{0},\mathbb{A})I(\|\mathbf{h}\|_2\leq c)}{\int_{\|\mathbf{h}\|_2\leq c}\phi(\mathbf{h},\mathbf{0},\mathbb{A})d\mathbf{h}},\ \phi(\mathbf{v},\boldsymbol{\mu},\Sigma)=\frac{\exp\left(-(\mathbf{v}-\boldsymbol{\mu})^\intercal\Sigma^{-1}(\mathbf{v}-\boldsymbol{\mu})/2\right)}{(2\pi)^{-q}\det(\Sigma)^{-1/2}}.$$

Define the marginal distribution of i.i.d. data $\mathscr{D}_N=\{(R_i,R_i\mathbf{Z}_i,\mathbf{W}_i):i=1,\ldots,N\}$ *as*

$$\mathscr{P}(\mathscr{D}_N)=\int_{\mathbf{h}}\prod_{i=1}^N\{\rho f_{\mathbf{h}}(\mathbf{Z}_i,\mathbf{W}_i)\}^{R_i}\left\{(1-\rho)\int_{\mathbf{v}}f_{\mathbf{h}}(\mathbf{v},\mathbf{W}_i)d\mathbf{v}\right\}^{1-R_i}p(\mathbf{h};c,\mathbb{A})d\mathbf{h}$$

and posterior $\mathbf{h}\mid\mathscr{D}_N$ *as*

$$\mathscr{Q}(\mathbf{h}\mid\mathscr{D}_N)=\frac{\prod_{i=1}^N\{\rho f_{\mathbf{h}}(\mathbf{Z}_i,\mathbf{W}_i)\}^{R_i}\left\{(1-\rho)\int_{\mathbf{v}}f_{\mathbf{h}}(\mathbf{v},\mathbf{W}_i)d\mathbf{v}\right\}^{1-R_i}p(\mathbf{h};c,\mathbb{A})}{\mathscr{P}(\mathscr{D}_N)}.$$

With finite variances of $\mathbf{g}_1(\mathbf{Z},\mathbf{W})$ *and* $\mathbf{g}_2(\mathbf{W})$*, the posterior* $\mathscr{Q}(\mathbf{h}\mid\mathscr{D}_N)$ *is approximated by the Gaussian posterior* $\phi(\mathbf{h},\widetilde{\boldsymbol{\mu}},\widetilde{\mathcal{V}})$*,*

$$\lim_{c,N\to\infty}\int\left\|\mathscr{Q}(\mathbf{h}\mid\mathscr{D}_N)-\phi(\mathbf{h},\widetilde{\boldsymbol{\mu}},\widetilde{\mathcal{V}})\right\|_{\mathsf{TV}}\mathscr{P}(\mathscr{D}_N)d\mathscr{D}_N=0.$$

Proof [Proof of Lemma A26] The proof extends Theorem 2 page 15 of Duchi (2021) to two-way tilted sub-models for characterizing semi-supervised learning setting. it suffices to analyze the difference in conditional densities. In the proof of Theorem 13, we defined the empirical processes

$$\mathbf{V} = (\mathbf{V}_1^\intercal, \mathbf{V}_2^\intercal)^\intercal,\ \mathbf{V}_1 = -\sum_{i=1}^N R_i \mathbf{g}_1(\mathbf{Z}_i, \mathbf{W}_i)/\sqrt{n},\ \mathbf{V}_2 = -\sum_{i=1}^N \mathbf{g}_2(\mathbf{W}_i)/\sqrt{N} \to \mathbf{V}_2$$

characterizing the local asymptotic normality (LAN) property of the two-way tilted sub-model. Consider the event indicator

$$\mathscr{E}_{N,b} = \mathrm{I}\left\{ \left\| \mathcal{V}_\psi^{-1} \mathbf{V} \right\|_2 \le b \right\}. \tag{A.121}$$

With finite variances of $\mathbf{g}_1(\mathbf{Z}, \mathbf{W})$ and $\mathbf{g}_2(\mathbf{W})$, we apply Le Cam and Yang (2000) Chapter 6.3 Proposition 2 to obtain

a) Event $\mathscr{E}_{N,b}$ occurs with large probability: there exists sufficiently large $b_{c,\varepsilon}$ and $N_{c,\varepsilon}$ such that

$$\mathbb{E}_{\mathbf{h}}(\mathscr{E}_{N,b}) \ge 1 - \varepsilon,\ \forall \|\mathbf{h}\|_2 \le c, N \ge N_{c,\varepsilon}, b \ge b_{c,\varepsilon}; \tag{A.122}$$

b) Approximation of tilted model

$$d\mathscr{M}_{\mathbf{h}}(\mathscr{D}_N) = \prod_{i=1}^N \{\rho f_{\mathbf{h}}(\mathbf{Z}_i, \mathbf{W}_i)\}^{R_i} \left\{ (1-\rho) \int_{\mathbf{v}} f_{\mathbf{h}}(\mathbf{v}, \mathbf{W}_i) d\mathbf{v} \right\}^{1-R_i} d\mathscr{D}_N$$

by Gaussian model

$$d\mathscr{G}_{\mathbf{h}}(\mathscr{D}_N) = \exp\left\{ -\frac{1}{2} (\mathbf{h} - \mathcal{V}_\psi^{-1}\mathbf{V})^\intercal \mathcal{V}_\psi (\mathbf{h} - \mathcal{V}_\psi^{-1}\mathbf{V}) \right\} d\mathscr{M}_{\mathbf{0}}(\mathscr{D}_N)$$

$$\lim_{N\to\infty} \sup_{\|\mathbf{h}\|\le c} \int \mathscr{E}_{N,b} \left| d\mathscr{M}_{\mathbf{h}}(\mathscr{D}_N) - d\mathscr{G}_{\mathbf{h}}(\mathscr{D}_N) \right|. \tag{A.123}$$

In the following, we define a series models to link the exact posterior and its Gaussian approximation. First, we define the model restricted to the "good set" on which $\mathscr{E} = 1$ in the model,

$$\begin{aligned} d\mathscr{M}_{\mathbf{h}}^{\mathscr{E}}(\mathscr{D}_N) &= \mathscr{E} d\mathscr{M}_{\mathbf{h}}(\mathscr{D}_N), \\ d\mathscr{G}_{\mathbf{h}}^{\mathscr{E}}(\mathscr{D}_N) &= \exp\left\{ -\frac{1}{2} (\mathbf{h} - \mathcal{V}_\psi^{-1}\mathbf{V})^\intercal \mathcal{V}_\psi (\mathbf{h} - \mathcal{V}_\psi^{-1}\mathbf{V}) \right\} d\mathscr{M}_{\mathbf{0}}^{\mathscr{E}}(\mathscr{D}_N). \end{aligned} \tag{A.124}$$

Using the newly defined notations in (A.123), we have

$$\lim_{N\to\infty} \sup_{\|\mathbf{h}\|\le c} \|\mathscr{M}_{\mathbf{h}}^{\mathscr{E}} - \mathscr{G}_{\mathbf{h}}^{\mathscr{E}}\|_{\mathsf{TV}} = 0. \tag{A.125}$$

Next, we define the exact, approximate Gaussian and $\mathscr{E}$-restricted joint distributions with truncated prior and another approximate Gaussian joint distributions with (untruncated) Gaussian prior

$$d\mathscr{J}(\mathscr{D}_N, \mathbf{h}) = d\mathscr{M}_{\mathbf{h}}(\mathscr{D}_N) p(\mathbf{h}; c, \mathbb{A}) d\mathbf{h},$$

$$
\begin{aligned}
d\mathscr{J}^{\mathscr{G}}(\mathscr{D}_N,\mathbf{h}) &= d\mathscr{G}^{\mathscr{E}}_{\mathbf{h}}(\mathscr{D}_N)p(\mathbf{h};c,\mathbb{A})d\mathbf{h},\\
d\mathscr{J}^{\mathscr{E}}(\mathscr{D}_N,\mathbf{h}) &= d\mathscr{M}^{\mathscr{E}}_{\mathbf{h}}(\mathscr{D}_N)p(\mathbf{h};c,\mathbb{A})d\mathbf{h},\\
d\mathscr{J}^{\mathscr{P}}(\mathscr{D}_N,\mathbf{h}) &= d\mathscr{G}^{\mathscr{E}}_{\mathbf{h}}(\mathscr{D}_N)\phi(\mathbf{h},\mathbf{0},\mathbb{A})d\mathbf{h}.
\end{aligned}
\tag{A.126}
$$

Since the truncated prior $p(\mathbf{h};c,\mathbb{A})$ restricts $\mathbf{h}$ to $\|\mathbf{h}\|_2 \le c$, we may bound the difference between $d\mathscr{J}^{\mathscr{G}}$ and $d\mathscr{J}^{\mathscr{E}}$ by (A.125),

$$
\lim_{N\to\infty}\|\mathscr{J}^{\mathscr{G}}_{\mathbf{h}} - \mathscr{J}^{\mathscr{E}}_{\mathbf{h}}\|_{\mathsf{TV}} \le \lim_{N\to\infty}\int \|\mathscr{G}^{\mathscr{E}}_{\mathbf{h}} - \mathscr{M}^{\mathscr{E}}_{\mathbf{h}}\|_{\mathsf{TV}} p(\mathbf{h};c,\mathbb{A})d\mathbf{h} = 0. \tag{A.127}
$$

Since $\mathbb{E}_{\mathbf{h}}\{\mathscr{E}\} \ge 1-\varepsilon$ uniformly in $\|\mathbf{h}\|_2 \le c$, we can control the error from restricting measure in $\{\mathscr{E}=1\}$ for sufficiently large N,

$$
\|\mathscr{J} - \mathscr{J}^{\mathscr{E}}\|_{\mathsf{TV}} \le \int \|\mathscr{M}^{\mathscr{E}}_{\mathbf{h}} - \mathscr{M}^{\mathscr{E}}_{\mathbf{h}}\|_{\mathsf{TV}} p(\mathbf{h};c,\mathbb{A})d\mathbf{h} = \int (1-\mathscr{E})p(\mathbf{h};c,\mathbb{A})d\mathbf{h} \le \varepsilon. \tag{A.128}
$$

As the final link, we control the error from truncation in prior

$$
\begin{aligned}
\|\mathscr{J}^{\mathscr{G}} - \mathscr{J}^{\mathscr{P}}\|_{\mathsf{TV}} &= \int |d\mathscr{G}^{\mathscr{E}}_{\mathbf{h}}(\mathscr{D}_N)p(\mathbf{h};c,\mathbb{A})d\mathbf{h} - d\mathscr{G}^{\mathscr{E}}_{\mathbf{h}}(\mathscr{D}_N)\phi(\mathbf{h},\mathbf{0},\mathbb{A})d\mathbf{h}|\\
&\le \int \sup_{\mathbf{h}} d\mathscr{G}^{\mathscr{E}}_{\mathbf{h}}(\mathscr{D}_N)\|\phi(\mathbf{h},\mathbf{0},\mathbb{A}) - p(\mathbf{h};c,\mathbb{A})\|_{\mathsf{TV}}\\
&\le \int \exp\left(\frac{1}{2}\mathbf{V}^{\intercal}\mathcal{V}_{\psi}^{-1}\mathbf{V}\right) d\mathscr{M}^{\mathscr{E}}_{\mathbf{0}}(\mathscr{D}_N)\|\phi(\mathbf{h},\mathbf{0},\mathbb{A}) - p(\mathbf{h};c,\mathbb{A})\|_{\mathsf{TV}}.
\end{aligned}
\tag{A.129}
$$

Over the restricted measure $\mathscr{M}^{\mathscr{E}}_{\mathbf{0}}$, $\|\mathcal{V}_{\psi}^{-1}\mathbf{V}\|_2 \le b$ is bounded, so

$$
\mathscr{E} = 1 : \exp\left(\frac{1}{2}\mathbf{V}^{\intercal}\mathcal{V}_{\psi}^{-1}\mathbf{V}\right) \le \exp(\|\mathcal{V}_{\psi}\|_2 b^2/2) = O(1).
$$

By choosing sufficiently large c such that

$$
\int_{\|\mathbf{h}\|_2 > c} \phi(\mathbf{h},\mathbf{0},\mathbb{A})d\mathbf{h} \le \varepsilon/\{2\exp(\|\mathcal{V}_{\psi}\|_2 b^2/2)\},
$$

we can control the truncation error in prior

$$
\|\phi(\mathbf{h},\mathbf{0},\mathbb{A}) - p(\mathbf{h};c,\mathbb{A})\|_{\mathsf{TV}} \le \varepsilon/\exp(\|\mathcal{V}_{\psi}\|_2 b^2/2).
$$

Apply the two bounds above to (A.129), we obtain

$$
\|\mathscr{J}^{\mathscr{G}} - \mathscr{J}^{\mathscr{P}}\|_{\mathsf{TV}} \le \varepsilon. \tag{A.130}
$$

Through (A.127), (A.128), (A.130) and (A.130), we have established the approximation among joint measures defined in (A.126) and in particular

$$
\limsup_{N\to\infty}\|\mathscr{J} - \mathscr{J}^{\mathscr{P}}\|_{\mathsf{TV}} \le 2\varepsilon. \tag{A.131}
$$

To derive the approximation in posterior distribution from the approximation of joint distribution, we invoke the following lemma,

Lemma A27 (Le Cam and Yang (2000) Chapter 6.4 Lemma 2 page 136) *Let*

$$\mathscr{M}_j(d\mathscr{D}, d\boldsymbol{\theta}) = \nu_j(d\mathscr{D})\mathscr{M}_j(d\boldsymbol{\theta} \mid \mathscr{D}),\ j = 1, 2,$$

be two joint measure for $(\mathscr{D}, \boldsymbol{\theta})$*. Then, the difference of conditional distributions in total variation is controlled by the difference of joint distributions in total variation*

$$\int \|\mathscr{M}_1(d\boldsymbol{\theta} \mid \mathscr{D}) - \mathscr{M}_2(d\boldsymbol{\theta} \mid \mathscr{D})\|_{\mathsf{TV}} |\nu_1(d\mathscr{D}) + \nu_2(d\mathscr{D})| \leq 4\|\mathscr{M}_1(d\mathscr{D}, d\boldsymbol{\theta}) - \mathscr{M}_2(d\mathscr{D}, d\boldsymbol{\theta})\|_{\mathsf{TV}}.$$

Notice that the joint measure $\mathscr{J}^{\mathscr{P}}$ has (untruncated) Gaussian prior and approximated Gaussian model, we can explicitly derive its posterior

$$d\mathscr{J}^{\mathscr{P}} = \phi(\mathbf{h}, \widetilde{\boldsymbol{\mu}}, \widetilde{\mathcal{V}})d\mathbf{h}\phi(\mathbf{V}, \mathbf{0}, \mathcal{V}_\varepsilon + \mathbb{A})d\mathscr{M}_{\mathbf{0}}(\mathscr{D}_N).$$

Applying Lemma A27 with (A.131), we have proven

$$\begin{aligned}
&\lim_{N\to\infty} \int \left\|\mathscr{Q}(\mathbf{h} \mid \mathscr{D}_N) - \phi(\mathbf{h}, \widetilde{\boldsymbol{\mu}}, \widetilde{\mathcal{V}})\right\|_{\mathsf{TV}} \mathscr{P}(\mathscr{D}_N)d\mathscr{D}_N \\
\leq &\lim_{N\to\infty} \int \left\|\mathscr{Q}(\mathbf{h} \mid \mathscr{D}_N) - \phi(\mathbf{h}, \widetilde{\boldsymbol{\mu}}, \widetilde{\mathcal{V}})\right\|_{\mathsf{TV}} |\mathscr{P}(\mathscr{D}_N)d\mathscr{D}_N + \phi(\mathbf{V}, \mathbf{0}, \mathcal{V}_\varepsilon + \mathbb{A})d\mathscr{M}_{\mathbf{0}}(\mathscr{D}_N)| \\
\leq &\lim_{N\to\infty} 4\|\mathscr{J} - \mathscr{J}^{\mathscr{P}}\|_{\mathsf{TV}} \\
\leq &2\varepsilon.
\end{aligned}$$

Setting $c \to \infty$ thus $\varepsilon \to 0$ yields

$$\lim_{c\to\infty} \lim_{N\to\infty} \int \left\|\mathscr{Q}(\mathbf{h} \mid \mathscr{D}_N) - \phi(\mathbf{h}, \widetilde{\boldsymbol{\mu}}, \widetilde{\mathcal{V}})\right\|_{\mathsf{TV}} \mathscr{P}(\mathscr{D}_N)d\mathscr{D}_N = 0.$$

We have proven that the asymptotic posterior follows the Gaussian distribution $N(\widetilde{\boldsymbol{\mu}}, \widetilde{\mathcal{V}})$.
■
